# Supplementary material for: Career intentions of medical students in the UK: a national, cross-sectional study (AIMS study)
Source: BMJ Open. 2023 Sep 12;13(9):e075598. doi: 10.1136/bmjopen-2023-075598 (PMC10496670; doi:10.1136/bmjopen-2023-075598)
Supplement: Supplementary data [file bmjopen-2023-075598supp012.pdf]

Eligible Medical Schools and Approved Programmes

A combination of the universities of Dundee and St. Andrews (ScotGEM)
A combination of the University of Brighton and the University of Sussex
A combination of the University of Hull and the University of York
Anglia Ruskin School of Medicine
Aston Medical School
Brunel University London Medical School
Cardiff University
Edge Hill University Medical School
Imperial College London
Keele University
Kent and Medway Medical School
King's College London
Lancaster University
Queen Mary University of London
St George's University of London
Swansea University
The Queen's University of Belfast
The University of Aberdeen
The University of Birmingham
The University of Bristol
The University of Buckingham
The University of Cambridge
The University of Central Lancashire
The University of Dundee
The University of Dundee
The University of East Anglia
The University of Edinburgh
The University of Exeter
The University of Glasgow
The University of Leeds
The University of Leicester
The University of Liverpool
The University of Manchester
The University of Newcastle
The University of Nottingham
The University of Oxford
The University of Plymouth
The University of Sheffield
The University of Southampton
The University of St Andrew's
The University of Warwick
Ulster University School of Medicine
University College London
University of Sunderland School of Medicine

Excluded for lack of cohort at time of recruitment:

- University of Chester Medical School
- Three Counties Medical School
