# Supplementary figures and images for: Career intentions of medical students in the UK: a national, cross-sectional study (AIMS study)
Source: BMJ Open. 2023 Sep 12;13(9):e075598. doi: 10.1136/bmjopen-2023-075598 (PMC10496670; doi:10.1136/bmjopen-2023-075598)

# Total responses by medical school

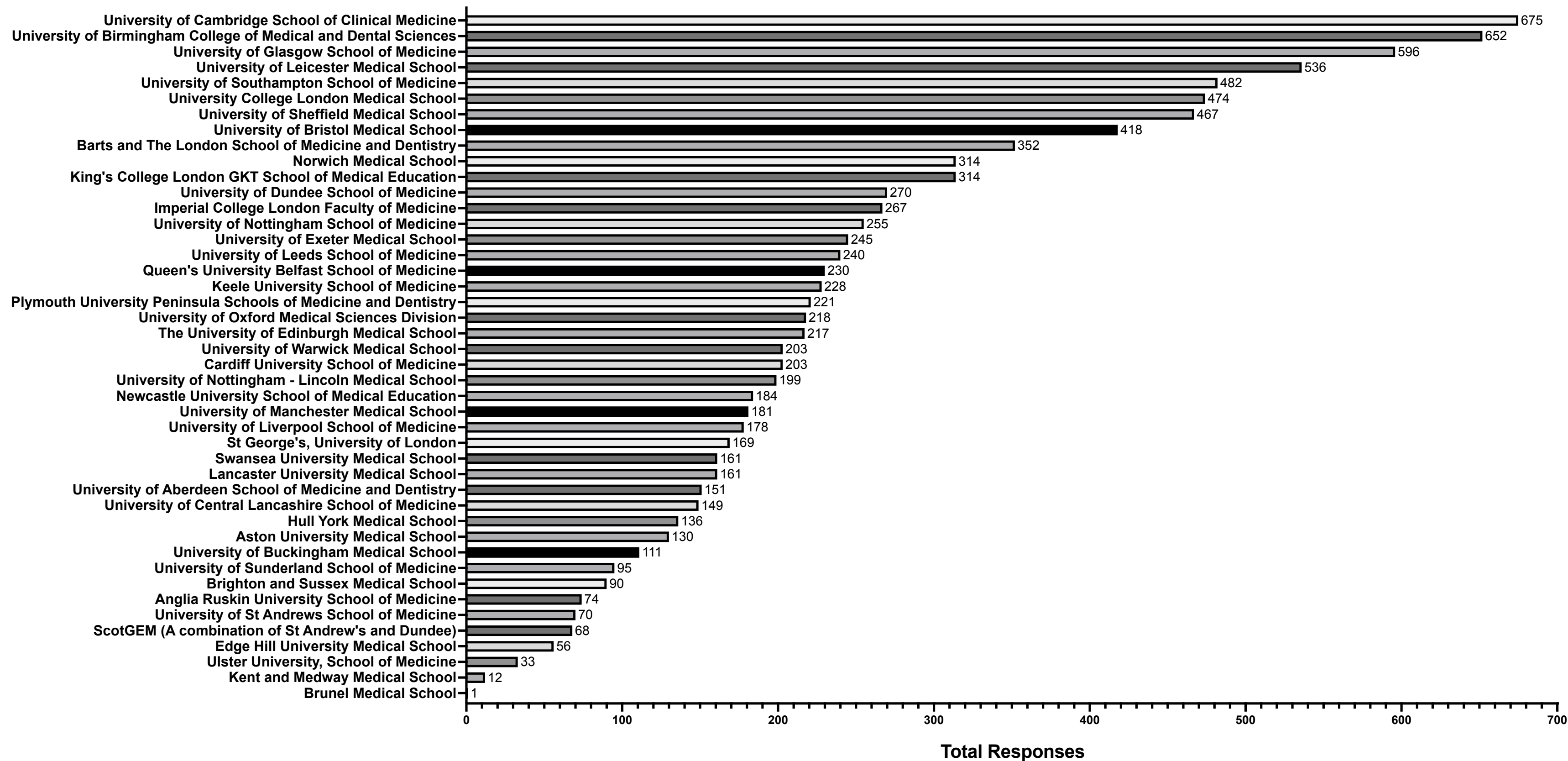

Supplement: Supplementary data [file bmjopen-2023-075598supp001.pdf]

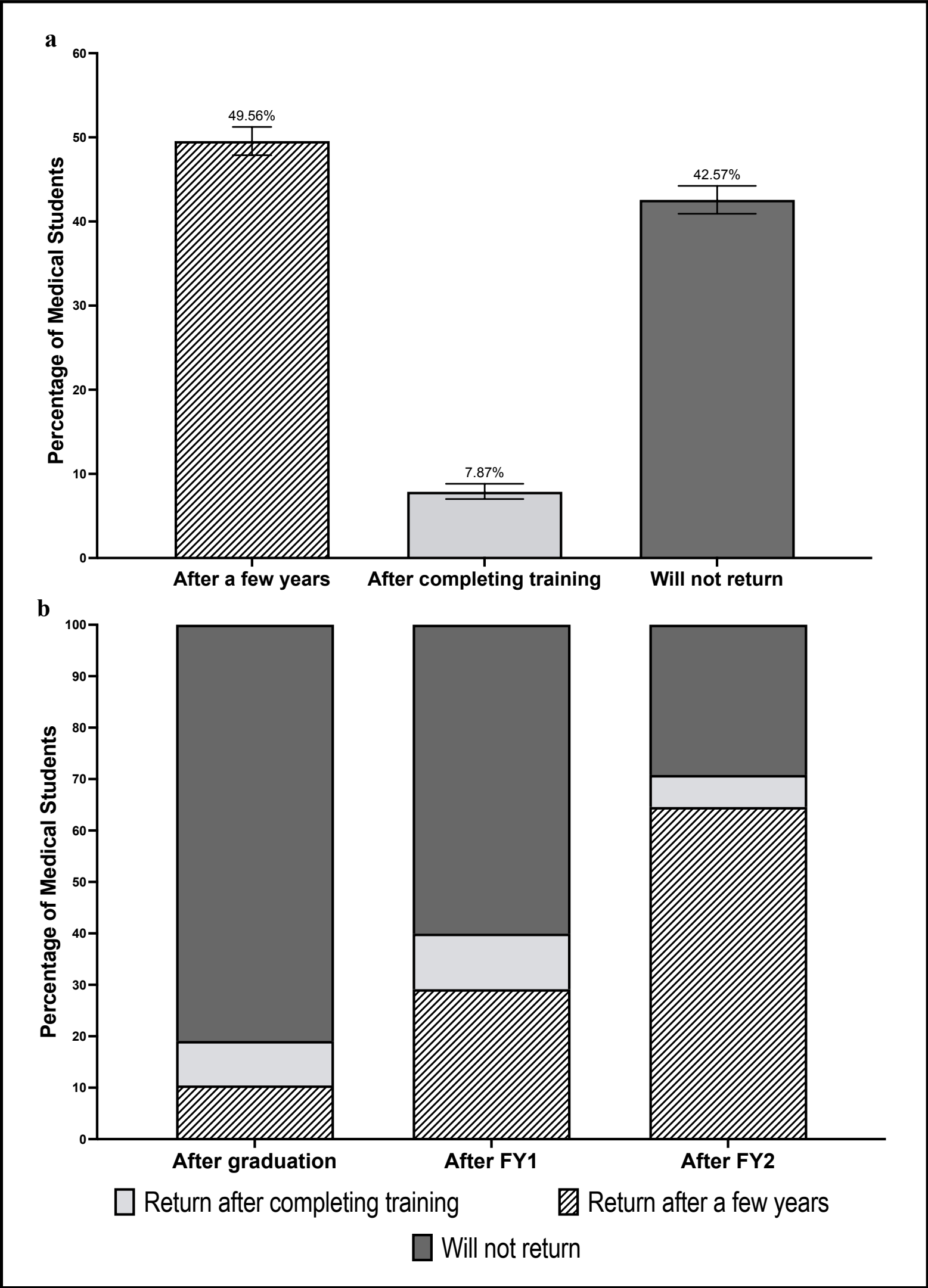

Supplement: Supplementary data [file bmjopen-2023-075598supp002.pdf]

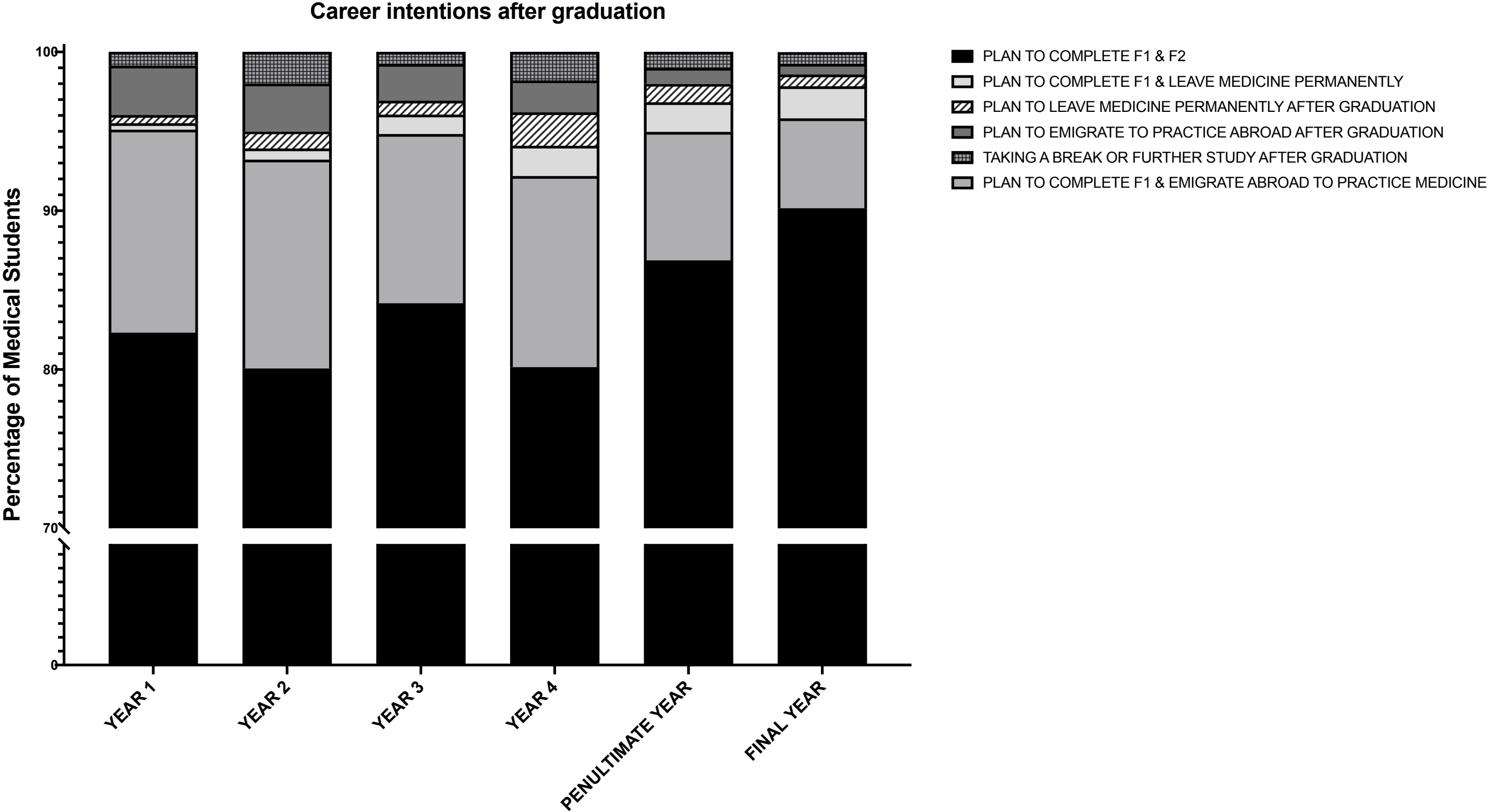

Supplement: Supplementary data [file bmjopen-2023-075598supp003.pdf]

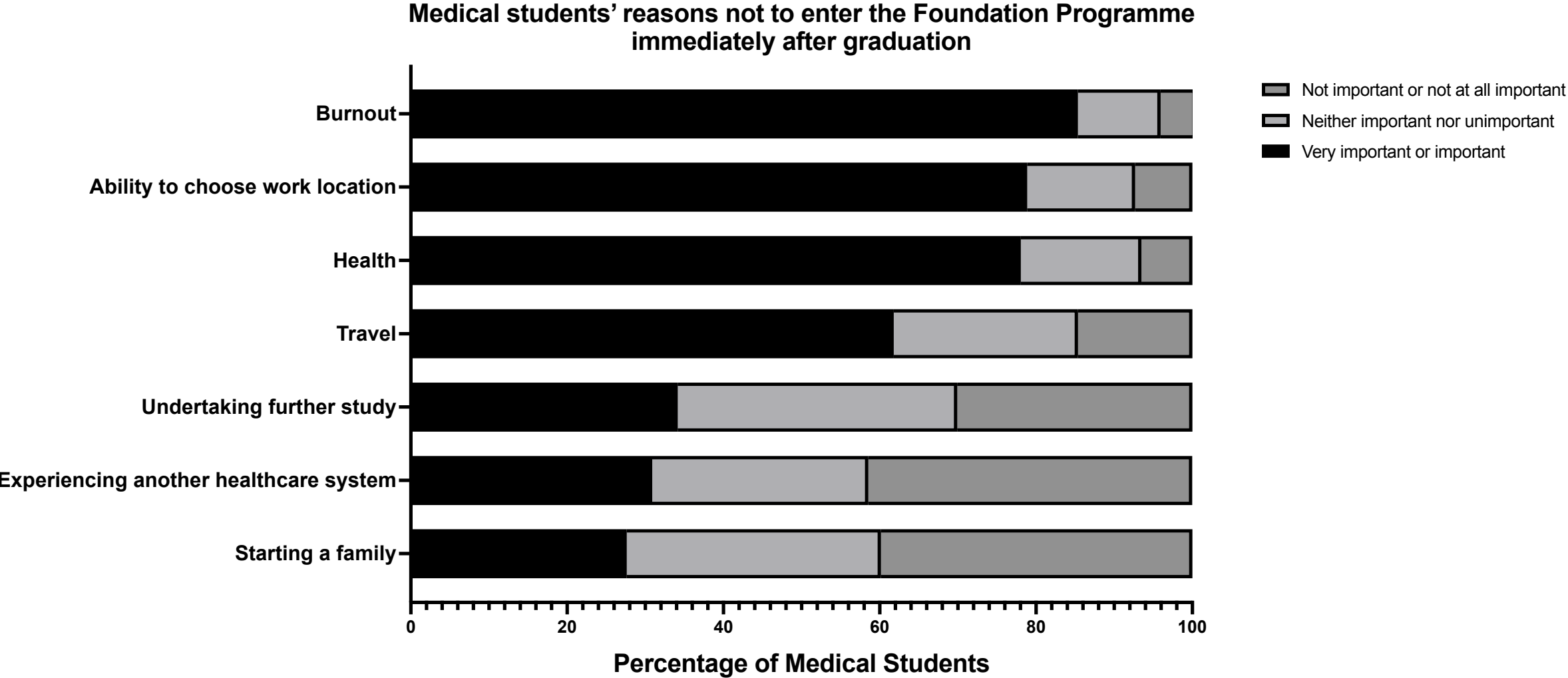

Supplement: Supplementary data [file bmjopen-2023-075598supp004.pdf]

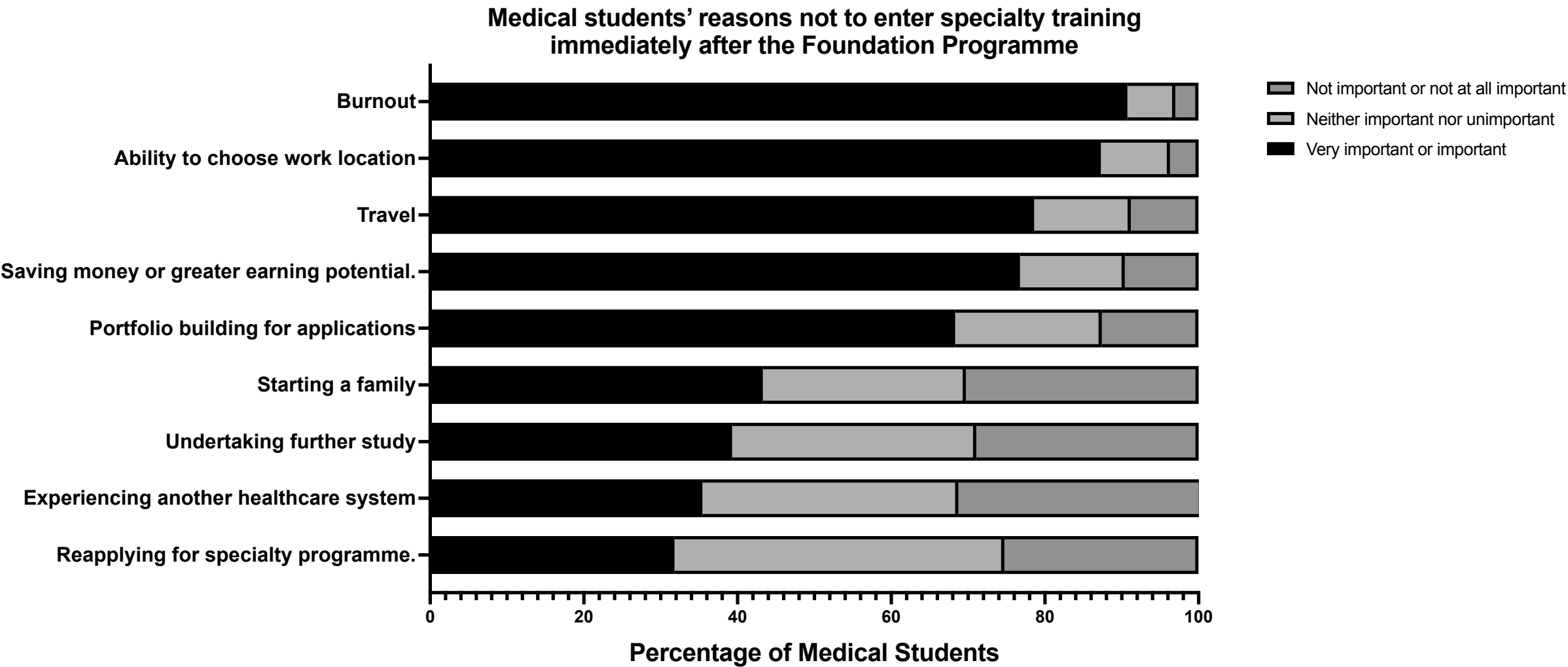

Supplement: Supplementary data [file bmjopen-2023-075598supp005.pdf]
