# Supplementary material for: Career intentions of medical students in the UK: a national, cross-sectional study (AIMS study)
Source: BMJ Open. 2023 Sep 12;13(9):e075598. doi: 10.1136/bmjopen-2023-075598 (PMC10496670; doi:10.1136/bmjopen-2023-075598)
Supplement: Supplementary data [file bmjopen-2023-075598supp006.pdf]

Students' intention after graduation	Number (%)	Confidence Interval
Complete both FY1 and FY2	8,806 (83.98)	[83.26, 84.67]
Complete FY1 and emigrate to practice medicine	1,101 (10.50)	[9.93,11.10]
Complete FY1 and leave medicine permanently	132 (1.26)	[1.06, 1.49]
Leave medicine permanently	104 (0.99)	[0.82, 1.20]
Emigrate to practice medicine	220 (2.10)	[1.84, 2.39]
Take a break or undertake further study	123 (1.17)	[0.98, 1.40]
