# Supplementary material for: Career intentions of medical students in the UK: a national, cross-sectional study (AIMS study)
Source: BMJ Open. 2023 Sep 12;13(9):e075598. doi: 10.1136/bmjopen-2023-075598 (PMC10496670; doi:10.1136/bmjopen-2023-075598)
Supplement: Supplementary data [file bmjopen-2023-075598supp007.pdf]

Students' intention after the Foundation Programme	Number (%)	Confidence Interval
Enter specialty training in the UK	4,294 (48.76)	[47.72, 49.81]
Assume a non-training clinical job in the UK	1,859 (21.11)	[20.27, 21.98]
Emigrate to practice medicine abroad (including temporarily)	2,071 (23.52)	[22.64, 24.42]
Take a break or undertake further study	515 (5.85)	[5.38, 6.36]
Leave medicine permanently	67 (0.76)	[0.60, 0.97]
