# Supplementary material for: Career intentions of medical students in the UK: a national, cross-sectional study (AIMS study)
Source: BMJ Open. 2023 Sep 12;13(9):e075598. doi: 10.1136/bmjopen-2023-075598 (PMC10496670; doi:10.1136/bmjopen-2023-075598)
Supplement: Supplementary data [file bmjopen-2023-075598supp010.pdf]

Demographic subgroup	Return prospects		
	After a few years	After completing training	Will not return
Ethnicity			
White	1,133 (58.46)	131 (6.76)	674 (34.78)
Asian or Asian British	334 (36.66)	78 (8.56)	499 (54.77)
Black, Black British, Caribbean or African	79 (44.89)	15 (8.52)	82 (46.59)
Mixed or multiple ethnic groups	88 (46.07)	23 (12.04)	80 (41.88)
Other	40 (28.37)	18 (12.77)	83 (58.87)
Prefer not to say	7 (20.00)	2 (5.71)	26 (74.29)
Gender			
Female	1,165 (53.37)	175 (8.02)	843 (38.62)
Male	512 (42.99)	92 (7.72)	587 (49.29)
Non-binary	3 (25.00)	0 (0)	9 (75.00)
Prefer not to say	1 (16.67)	0 (0)	5 (83.33)
Level of education			
Postgraduate	311 (46.49)	51 (7.62)	307 (45.89)
Undergraduate	1,370 (50.31)	216 (7.93)	1137 (41.76)
Previous schooling			
Private education	578 (44.91)	113 (8.78)	596 (46.31)
State education	1,072 (52.96)	143 (7.07)	809 (39.97)

Prefer not to say	31 (38.27)	11 (13.58)	39 (48.15)
Fee status			
Home	1,572 (56.67)	221 (7.97)	981 (35.36)
EU	45 (20.74)	17 (7.83)	155 (71.43)
International (Non-EU)	64 (15.96)	29 (7.23)	308 (76.81)
Current year of study			
Year 1	297 (46.05)	65 (10.08)	283 (43.88)
Year 2	346 (48.53)	55 (7.71)	312 (43.76)
Year 3	281 (47.15)	55 (9.23)	260 (43.62)
Year 4 (not penultimate year)	163 (50.00)	25 (7.67)	138 (42.33)
Penultimate year	313 (50.81)	47 (7.63)	256 (41.56)
Final year	281 (56.65)	20 (4.03)	195 (39.31)
Total	1681 (49.56)	267 (7.87)	1444 (42.57)
