# Supplementary material for: Career intentions of medical students in the UK: a national, cross-sectional study (AIMS study)
Source: BMJ Open. 2023 Sep 12;13(9):e075598. doi: 10.1136/bmjopen-2023-075598 (PMC10496670; doi:10.1136/bmjopen-2023-075598)
Supplement: Supplementary data [file bmjopen-2023-075598supp008.pdf]

**Students' intention after graduation**	**Year 1**	**Year 2**	**Year 3**	**Year 4 (not penultimate year)**	**Penultimate Year**	**Final Year**
Complete both FY1 and FY2	1616 (82.32)	1723 (80.07)	1643 (84.17)	759 (80.15)	1728 (86.88)	1337 (90.16)
Complete FY1 and emigrate to practice medicine	251 (12.79)	283 (13.15)	208 (10.66)	114 (12.04)	161 (8.09)	84 (5.66)
Complete FY1 and leave medicine permanently	8 (0.41)	15 (0.70)	24 (1.23)	18 (1.90)	37 (1.86)	30 (2.02)
Leave medicine permanently	10 (0.51)	23 (1.07)	17 (0.87)	20 (2.11)	23 (1.16)	11 (0.74)
Emigrate to practice medicine	61 (3.11)	65 (3.02)	45 (2.31)	19 (2.01)	20 (1.01)	10 (0.67)
Take a break or undertake further study	17 (0.87)	43 (2.00)	15 (0.77)	17 (1.80)	20 (1.01)	11 (0.74)
