# Supplementary material for: Career intentions of medical students in the UK: a national, cross-sectional study (AIMS study)
Source: BMJ Open. 2023 Sep 12;13(9):e075598. doi: 10.1136/bmjopen-2023-075598 (PMC10496670; doi:10.1136/bmjopen-2023-075598)
Supplement: Supplementary data [file bmjopen-2023-075598supp009.pdf]

**Students' intention after the Foundation Programme**	**Year 1**	**Year 2**	**Year 3**	**Year 4 (not penultimate year)**	**Penultimate Year**	**Final Year**
Enter specialty training in the UK	1093 (67.64)	1071 (62.16)	866 (52.71)	298 (39.26)	621 (35.94)	345 (25.80)
Assume a non-training clinical job in the UK	109 (6.75)	197 (11.43)	326 (19.84)	192 (25.30)	554 (32.06)	481 (35.98)
Emigrate to practice medicine abroad (including temporarily)	333 (20.61)	365 (21.18)	343 (20.88)	193 (25.43)	435 (25.17)	402 (30.07)
Take a break or undertake further study	78 (4.83)	86 (4.99)	96 (5.84)	68 (8.96)	103 (5.96)	84 (6.28)
Leave medicine permanently	3 (0.19)	4 (0.23)	12 (0.73)	8 (1.05)	15 (0.87)	25 (1.87)
