# Supplementary material for: Career intentions of medical students in the UK: a national, cross-sectional study (AIMS study)
Source: BMJ Open. 2023 Sep 12;13(9):e075598. doi: 10.1136/bmjopen-2023-075598 (PMC10496670; doi:10.1136/bmjopen-2023-075598)
Supplement: Author's manuscript [file bmjopen-2023-075598.draft_revisions.pdf]

BMJ Open is committed to open peer review. As part of this commitment we make the peer review history of every article we publish publicly available.

When an article is published we post the peer reviewers' comments and the authors' responses online. We also post the versions of the paper that were used during peer review. These are the versions that the peer review comments apply to.

The versions of the paper that follow are the versions that were submitted during the peer review process. They are not the versions of record or the final published versions. They should not be cited or distributed as the published version of this manuscript.

BMJ Open is an open access journal and the full, final, typeset and author-corrected version of record of the manuscript is available on our site with no access controls, subscription charges or pay-per-view fees (http://bmjopen.bmj.com).

If you have any questions on BMJ Open's open peer review process please email info.bmjopen@bmj.com

BMJ Open

Career intentions of medical students in the United Kingdom: a national, cross-sectional study (AIMS Study)

Journal:	*BMJ Open*
Manuscript ID	bmjopen-2023-075598
Article Type:	Original research
Date Submitted by the Author:	15-May-2023
Complete List of Authors:	Ferreira, Tomas; University of Cambridge School of Clinical Medicine<br>Collins, Alexander; Imperial College London, School of Public Health, Faculty of Medicine<br>Feng, Oliver; University of Cambridge, Statistical Laboratory, Centre for Mathematical Sciences<br>Samworth, Richard; University of Cambridge, Statistical Laboratory<br>Horvath, Rita; University of Cambridge School of Clinical Medicine<br>., the AIMS Collaborative; University of Cambridge School of Clinical Medicine
Keywords:	Health policy < HEALTH SERVICES ADMINISTRATION & MANAGEMENT, QUALITATIVE RESEARCH, Health Education, Organisation of health services < HEALTH SERVICES ADMINISTRATION & MANAGEMENT, MEDICAL EDUCATION & TRAINING, Health economics < HEALTH SERVICES ADMINISTRATION & MANAGEMENT

SCHOLARONE™
Manuscripts

I, the Submitting Author has the right to grant and does grant on behalf of all authors of the Work (as defined in the below author licence), an exclusive licence and/or a non-exclusive licence for contributions from authors who are: i) UK Crown employees; ii) where BMJ has agreed a CC-BY licence shall apply, and/or iii) in accordance with the terms applicable for US Federal Government officers or employees acting as part of their official duties; on a worldwide, perpetual, irrevocable, royalty-free basis to BMJ Publishing Group Ltd ("BMJ") its licensees and where the relevant Journal is co-owned by BMJ to the co-owners of the Journal, to publish the Work in this journal and any other BMJ products and to exploit all rights, as set out in our licence.

The Submitting Author accepts and understands that any supply made under these terms is made by BMJ to the Submitting Author unless you are acting as an employee on behalf of your employer or a postgraduate student of an affiliated institution which is paying any applicable article publishing charge ("APC") for Open Access articles. Where the Submitting Author wishes to make the Work available on an Open Access basis (and intends to pay the relevant APC), the terms of reuse of such Open Access shall be governed by a Creative Commons licence – details of these licences and which Creative Commons licence will apply to this Work are set out in our licence referred to above.

Other than as permitted in any relevant BMJ Author's Self Archiving Policies, I confirm this Work has not been accepted for publication elsewhere, is not being considered for publication elsewhere and does not duplicate material already published. I confirm all authors consent to publication of this Work and authorise the granting of this licence.

For peer review only

Career intentions of medical students in the United Kingdom: a national, cross-sectional study (AIMS Study)

Tomas Ferreira[1], Alexander M. Collins[2], Oliver Feng[3], Richard J. Samworth[3], Rita Horvath[1], and the AIMS collaborative

Affiliations

[1] School of Clinical Medicine, University of Cambridge, Cambridge, United Kingdom

[2] School of Public Health, Faculty of Medicine, Imperial College London, London, United Kingdom

[3] Statistical Laboratory, Centre for Mathematical Sciences, University of Cambridge, Cambridge, United Kingdom

Postal address of corresponding author:

Tomas Ferreira
Cambridge Centre for Brain Repair, Ed Adrian Building,
Cambridge,
CB2 0PY
tf385@cam.ac.uk

For peer review only

Abstract

Introduction: Understanding medical students' intended career trajectories post-graduation is an important factor in effective workforce planning and retention.

Methods: Cross-sectional, mixed-methods survey of UK medical students distributed through a recruited network of approximately 200 collaborators.

Results: 10,486 responses were collected from all 44 medical schools in the UK. To the best of our knowledge, this is the largest ever study of UK medical students. The majority of students (8,806/10,486, 83.98%, CI: 83.26%, 84.67%) planned to complete both years of the Foundation Programme (FP) after graduation, with less than half of these students (4,294/8,806, 48.76%, CI: 47.72%, 49.81%) intending to pursue specialty training thereafter. A subanalysis of career intentions after the FP by year of study revealed a significant decrease in students' intentions to enter specialty training as they advanced through medical school. Approximately a third of students (3,392/10,486, 32.35%), with intended to emigrate to practice medicine, with 42.57% (CI: 40.92%, 44.24%) of those students not intending to return to the UK. 2.89% of students intended to leave medicine altogether. Remuneration, work-life balance, and working conditions were important factors in students' decision-making regarding emigration and leaving the profession. Subgroup analyses based on gender, type of schooling, fee type, and educational background were performed. Qualitative thematic analysis revealed that the most commonly cited issues included improvements to remuneration, flexibility and work-life balance, general working conditions, staffing levels, and greater autonomy in the location of work.

Conclusions: The AIMS study highlights UK students' views and career intentions, revealing a concerning proportion considering alternative careers or emigration. Addressing factors such as remuneration, work-life balance, and working conditions may improve retention of doctors. The study offers valuable insights and a

For peer review only

roadmap for improving prospects of working within the NHS, as these findings have implications for the future of the medical profession in the UK.

Trial registration details: N/A

Word count: 5,445

For peer review only

Introduction

Training doctors is a costly investment, and measuring the extent of attrition from the health service in the country of training is crucial to ensure optimal value. Understanding medical students' career plans and trajectories post-graduation is an important factor in effective workforce planning and retention.

There are several factors behind doctors' motivations to emigrate to practise medicine abroad or leave the profession entirely. Commonly cited themes include pay erosion and low pay compared to alternative

destinations, working conditions within the National Health Service (NHS), wellbeing, work-life balance, and better training opportunities abroad (1, 2).

The United Kingdom (UK) has 3.2 doctors for every 1,000 people, ranking 25th amongst the Organisation for Economic Co-operation and Development (OECD) countries. It has the lowest number of doctors per capita among European countries in the OECD (3). The British government has responded to this issue of an insufficient number of doctors by opening new medical schools and expanding the student capacity of existing ones (4, 5). Recently, there have been proposals to double the number of medical school places as a solution to address the shortage of doctors in the NHS (6). However, without addressing the issue of doctors leaving the NHS, increasing the number of medical students is unlikely to provide a sustainable long-term solution. Recruitment efforts may be ineffective if the retention of doctors is not simultaneously addressed. This highlights the pressing need for a multifaceted approach that considers both recruitment and retention strategies to effectively address the workforce challenges in the NHS.

To the best of our knowledge, this is the largest study of UK medical students to date. This mixed-methods study investigated current medical students' career intentions after graduation and upon completing the Foundation Programme, and the motivations behind these intentions. Secondary outcomes included determining which demographic factors alter the propensity to pursue different career paths available to a medical graduate, determining which specialties medical students plan to pursue and understanding current views on the prospect of working in the NHS. This data provides important answers to the current workforce challenges within the NHS and could help address some of the concerns of those making up the future of the profession.

Methods

Study Design

AIMS was a national, multi-centre, cross-sectional study of medical students conducted in accordance with its protocol (7). A novel, self-administered, 71-item questionnaire was developed. The survey was hosted on the Qualtrics survey platform (Provo, Utah, USA), a GDPR-compliant online platform that supports both mobile and desktop devices. Prior to completing the survey, all participants provided informed consent. All participants were asked to complete the first section of the survey (Questions 1 to 11). Subsequent question visibility was dependent on participants' answers to previous questions. The fewest number of items available to any one participant was 30, and the largest was 43. Questions were structured using a combination of Likert scale matrices, multiple-choice options, and free-text entry to broaden the capture of sentiment nuance and improve precision in the data. A copy of the questionnaire and the Participant Information Sheet can be found in the Supplemental Materials.

For peer review only

A network of approximately 200 collaborators across 42 medical schools was recruited prior to the study launch. All medical students in all year groups were eligible to apply, and positions were advertised via medical student societies, social media, and internal medical school newsletters. They were responsible for maximising the response numbers within their year group at their medical schools. Collaborators were instructed to use a range of distribution methods, including social media, internal bulletins/newsletters, and email communication. In order to qualify for collaborative authorship, students were required to achieve a minimum of 35 responses, or 15% of their year group (whichever number was lowest). The survey was disseminated between January 16, 2023, and March 27, 2023.

Eligible participants

To be eligible for participation, individuals must have been actively enrolled in a UK medical school acknowledged by the General Medical Council (GMC) and listed by the Medical School Council (MSC) (Supplemental Materials). Certain new medical schools have received approval from the GMC but have yet to admit their inaugural cohort of students. These schools were therefore excluded from our study since they had no medical students at the time of data collection.

Data Collection

The survey consisted of five parts. Part 1 involved a background and demographics section, which all participants were required to answer. In Part 2, participants were asked to indicate their intended career paths immediately after graduation and after foundation training (if applicable). Part 3 explored the factors influencing their decision-making. Part 4 surveyed their current specialty preferences. The final part featured a free-entry text box inviting participants to articulate how the prospect of working in the NHS could be improved. Consent for follow-up studies was also obtained in this section.

Data processing and storage

Each response was restricted to a single institutional email address to mitigate the risk of data duplication. Any replicated email entries were removed prior to data analysis. In cases where identical entries contained distinct responses, the most recent entry was retained. Entries where respondents did not provide a valid institutional email address were removed prior to data analysis to preserve the integrity of the study.

Quantitative data analysis

Descriptive analysis was carried out with Microsoft Excel (v16.71) (Arlington, Virginia, USA), and statistical inference was performed using RStudio (v4.2.1) (Boston, Massachusetts, USA). Tables and graphs were generated using GraphPad Prism (v9.5.0) (San Diego, California, USA). Odds ratios (OR), confidence

For peer review only

intervals (CI) and p-values were computed by fitting single-variable logistic regression models to explore the effect of various demographic characteristics on students' career intentions. We used $p<0.05$ to determine the statistical significance for all tests.

The findings of this study were reported in accordance with the STROBE (Strengthening the Reporting of Observational Studies in Epidemiology) guidelines (8).

Qualitative data analysis

Participants were asked to share which steps, if any, could be taken to improve the prospect of working in the NHS. The resulting qualitative data underwent inductive thematic analysis as per Braun and Clarke's method, with attention paid to reflexivity (9). Upon familiarisation with the data, responses were imported into Microsoft Excel and individually labelled with an initial set of codes. These codes and the patterns identified were later used to generate themes, which were then arranged into larger overarching categories. To account for the inherent subjectivity in the interpretation of free-text data, members of the authorship team discussed points of uncertainty until a consensus was reached.

Results

Demographics

In total, 10,486 students across all 44 medical schools in the UK participated in the survey. This represents approximately 21.50% of the medical student population in the UK. The mean response number per medical school was 244, and the median was 203 (IQR 135-281). A breakdown of the response numbers per medical school can be found in the Supplemental Materials. The median age for participants was 22 (IQR 20-23). Although responses were obtained from all year groups, there were relatively fewer responses from students in the 'Year 4 (not penultimate year)' category, due to a smaller number of students in intercalating courses or schools with six-year medical programmes, rather than the conventional five-year curriculum. Among the participants, 66.5% were female (n=6,977), 32.7% were male (n=3,429), 0.6% were non-binary (n=64), and 16 individuals preferred not to disclose their gender (*Table 1*).

For peer review only

Characteristic	Number (%)
Ethnicity	
White	5,838 (55.67)
Asian or Asian British	3,027 (28.87)
Black, Black British, Caribbean or African	529 (5.04)
Mixed or multiple ethnic groups	555 (5.29)
Other	410 (3.91)
Prefer not to say	127 (1.21)
Gender	
Female	6,977 (66.54)
Male	3,429 (32.70)
Non-binary	64 (0.61)
Prefer not to say	16 (0.15)
Level of education	
Postgraduate	1,873 (17.86)
Undergraduate	8,613 (82.14)
Previous schooling	
Private education	3,605 (34.38)
State education	6,609 (63.03)
Prefer not to say	272 (2.59)
Fee status	
Home	9,207 (87.80)
EU	419 (4.00)
International (Non-EU)	860 (8.20)
Current year of study	
Year 1	1,963 (18.72)
Year 2	2,152 (20.52)
Year 3	1,952 (18.62)
Year 4 (not penultimate year)	947 (9.03)
Penultimate Year	1,989 (18.97)
Final Year	1,483 (14.14)
Age	
Median (range)	22 (17-48)
Total	10,486 (100.00)

Table 1 – Demographic characteristics of participants

Career intentions

All participants were asked their current career intention for immediately after graduation, as shown in *Supplemental Table 1.* The majority of students (8,806/10,486, 83.98% (CI: 83.26%, 84.67%)) planned to complete both years of the UK's foundation training, Foundation Years 1 (FY1) and 2 (FY2); 10.50% (CI: 9.93%, 11.10%) intended to complete FY1 and then emigrate to practise medicine (n=1,101); 1.26% (CI: 1.06%, 1.49%) of students planned to complete FY1 and then permanently leave medicine (n=132); 0.99% (CI: 0.82%, 1.20%) of students intended to leave medicine permanently immediately after graduation (n=104); 2.10% (CI: 1.84%, 2.39%) of students planned to emigrate to practise medicine abroad immediately after

graduation (n=220); and 1.17% (CI: 0.98%, 1.40%) of students intended to take a break or undertake further study post-graduation (n=123).

Participants intending to complete both years of the Foundation Programme were then asked their intentions thereafter; the results can be seen in *Supplemental Table 2*. Of these 8,806 respondents, 48.76% (n=4,294, CI: 47.72%, 49.81%) planned to enter specialty training in the UK immediately after the Foundation Programme; 21.11% (n=1,859, CI: 20.27%, 21.98%) intended to enter a non-training clinical job in the UK (a common form of 'F3' year, including posts such as junior clinical fellowship (JCF) or clinical teaching fellowship (CTF), or working as a locum doctor). A further 23.52% of students (n=2,071, CI: 22.64%, 24.42%) intended to emigrate to practise medicine abroad, whilst 5.85% (n=515, CI: 5.38%, 6.36%) planned to take a break or undertake further study. 67 students (0.76%, CI: 0.60%, 0.97%) planned to leave medicine permanently.

Thirty-two per cent of medical students (n=3,392/10,486, 32.35%) intended to emigrate to practise medicine, either immediately after graduation (n=220/3,292, 6.49%), after completion of FY1 (n=1,101/3,292 32.46%) or after FY2 (n=2,071/3,292, 61.06%). These students were asked their likelihood of returning to UK medicine (return prospects); 49.56% (n=1,681, CI: 47.88%, 51.24%) stated they intended to return after a few years, whilst 7.87% (n=267, CI: 7.01%, 8.83%) intended to return after completion of their medical training abroad. The remaining 42.57% (n=1,444, CI: 40.92%, 44.24%) of students emigrating indicated no intentions to return (Supplemental Figure 2a). Of those intending to emigrate immediately after graduation, 80.91% did not intend to return to the UK (n=178/220). This number decreased to 60.03% (n=661/1101) in those emigrating after completing FY1, and 29.21% (n=605/2071) in those emigrating after completing FY2, as demonstrated in Supplemental Figure 2b.

All students intending to emigrate to practise medicine were asked the countries to which they were considering emigrating via a free-entry text box. Students were able to list multiple locations or say they were undecided. A total of 4,115 responses were received from 3,392 students. 25.03% of students (n=849) did not express a preference for any particular destination (Figure 1c). The remaining 2,543 medical students listed 3,266 destination preferences. Australia was the most commonly mentioned destination (42.35%), followed by New Zealand (18.03%), the United States (10.38%) and Canada (10.29%).

A total of 303/10,486 (2.89%) of medical students planned to leave the profession entirely, either immediately after graduating (n=104/303, 34.32%), after completion of FY1 (n=132/303, 43.56%), or after completion of FY2 (n=67/303, 22.11%). Students intending to leave the profession were asked the alternative industries they were considering for their future careers (Figure 1d). 21.12% (n=64/303) of those planning to leave the profession did not yet have an industry in mind. Of the remaining 78.88%, the most frequently mentioned career destinations included consulting, technology, financial services and law.

For peer review only - http://bmjopen.bmj.com/site/about/guidelines.xhtml

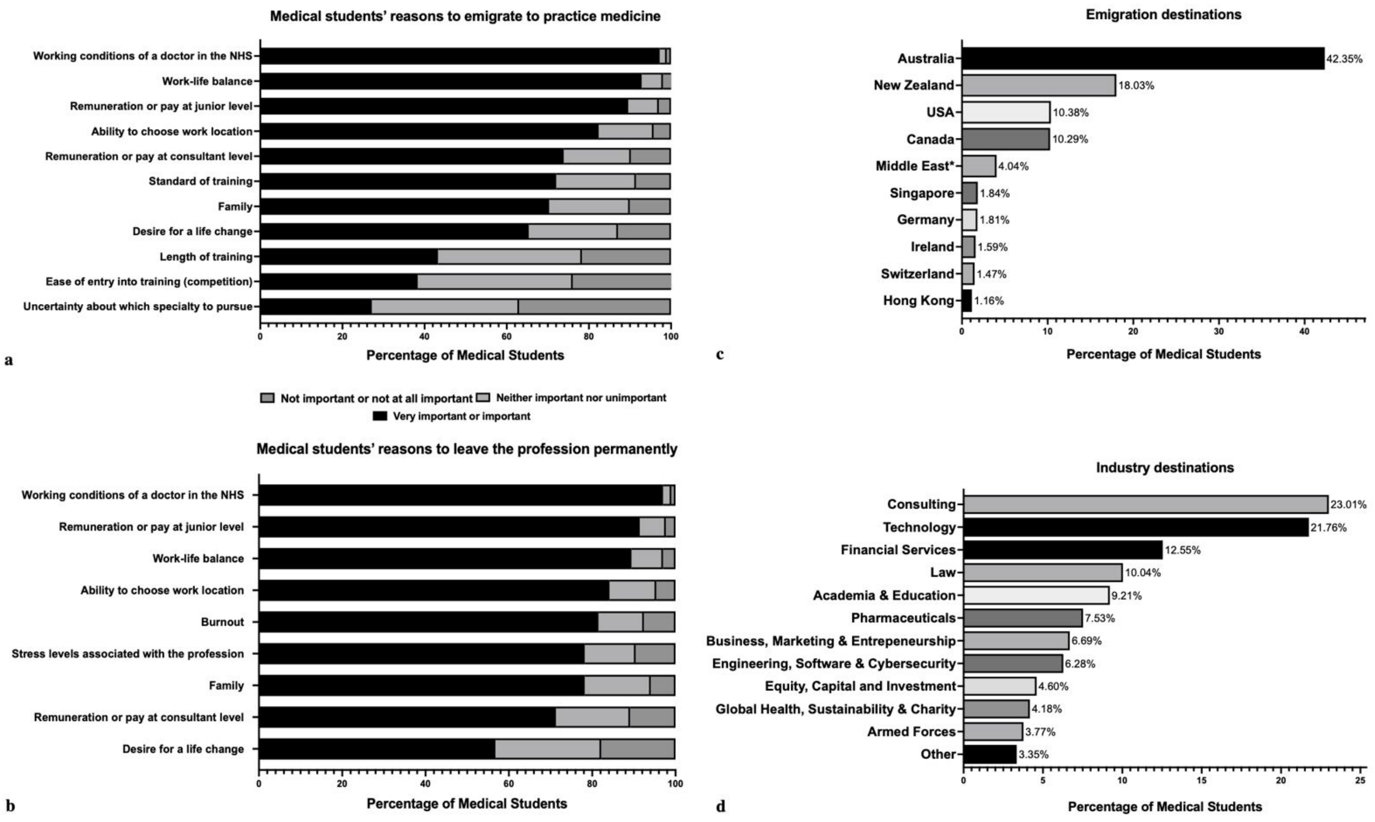

Figures 1.a-d - **a)** importance of factors influencing medical students' intention to emigrate and practise medicine; **b)** importance of factors influencing medical students' intention to leave the medical profession entirely and seek an alternative career; **c)** locations cited as potential destinations by students who intend to emigrate to practice medicine.; **d)** preferred industries to work in by those intending to leave medicine. *Several respondents cited the Middle East or Gulf region rather than specifying which country; these responses were grouped with individual destinations in the region

Career Intention Subanalyses

Subanalysis of career intentions after graduation by year of study revealed an overall increase in the proportion of students intending to complete the Foundation Programme as they progressed in their medical studies (*Supplemental Figure 3*). *Supplemental Tables 3 and 4* highlight students' career intentions after graduation and foundation programme, respectively, by year group.

For peer review only

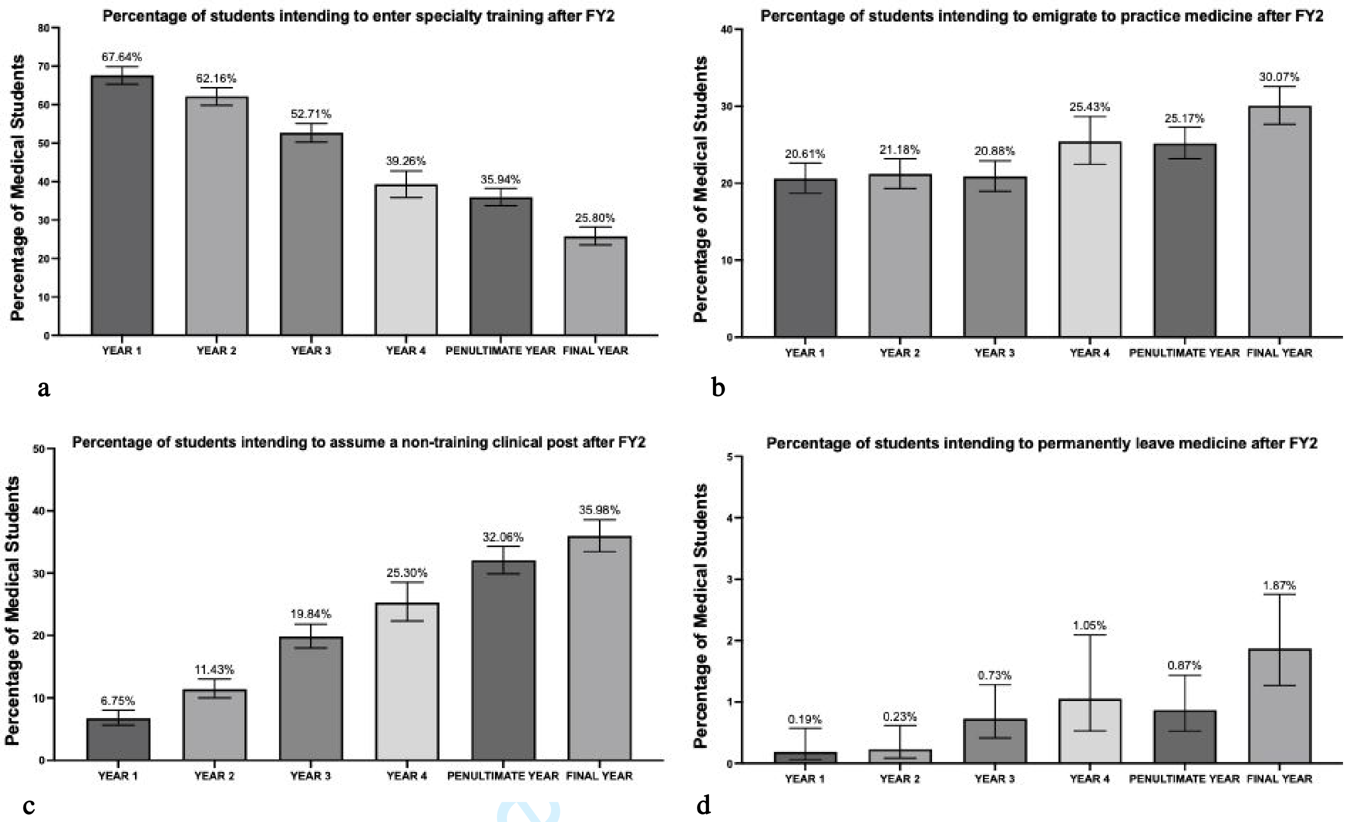

Figures 2.a-d - Proportions of students by year of study (with 95% confidence intervals) intending to **a)** directly enter specialty training after FY2; **b)** emigrate to practise medicine after FY2; **c)** enter a non-training clinical post after FY2, for example as a locum doctor or clinical fellow; **d)** leave medicine permanently after FY2 to pursue an alternative career. "Year 4" represents students in their fourth year of study, but not their penultimate year. Percentages in figures reflect the proportion of students in each year group for each intention.

Subanalysis of career intentions after completion of FY2 by current year of study revealed a significant decrease in the proportion of students intending to enter specialty training as they progressed in their medical studies (*Supplemental Table 4*). Student intentions to emigrate, permanently leave the profession and assume non-training clinical positions also increased as students advanced through medical school (*Figures 2, a-d*).

Subanalysis of the subgroup intending to leave medicine (n=303, 2.89%) revealed a significant difference in the proportion of students taking this decision by various demographic characteristics, as highlighted in *Table 2*. Specifically, males were significantly more likely to leave medicine than females (OR 2.61, $p<0.00001$), and state-educated students had a higher likelihood of leaving medicine compared to privately educated students (OR 1.28, $p<0.05$). Similarly, home students were more likely to leave medicine than non-home students, including international and non-EU students (OR 1.26, $p<0.00001$). However, we did not find a statistically significant difference between undergraduates and postgraduates in their likelihood of leaving medicine (OR 1.29, $p=0.124$).

We subanalysed the group of students intending to emigrate to practise by ethnicity, gender, stage of training, educational background, and previous schooling (*Table 2)*. Males were significantly more likely to emigrate to practise medicine than females (OR 1.17, $p<0.001$). Postgraduate students were significantly more likely

For peer review only

to emigrate to practise medicine than undergraduate students (OR 1.20, p<0.001). Privately educated students were significantly more likely to emigrate to practise medicine than their state educated peers (OR 1.26, p<0.00001). Non-home students (international and non-EU fees) were considerably more likely to emigrate to practise medicine than home students (OR 2.33, p<0.00001).

Demographic subgroup	Number intenting to leave medicine (%)	Number intenting to emigrate (%)
Ethnicity		
White	147 (2.52)	1,938 (33.20)
Asian or Asian British	99 (3.27)	911 (30.10)
Black, Black British, Caribbean or African	15 (2.84)	176 (33.27)
Mixed or multiple ethnic groups	24 (4.32)	191 (34.41)
Other	10 (2.44)	141 (34.39)
Prefer not to say	8 (6.30)	35 (27.56)
Gender		
Female	134 (1.92)	2,183 (31.29)
Male	167 (4.87)	1,191 (34.73)
Non-binary	1 (1.56)	12 (18.75)
Prefer not to say	1 (6.25)	6 (37.50)
Level of education		
Postgraduate	44 (2.35)	669 (35.72)
Undergraduate	259 (3.01)	2,723 (31.62)
Previous schooling		
Private education	118 (3.27)	1,287 (35.70)
State education	170 (2.57)	2,024 (30.62)
Prefer not to say	15 (5.51)	81 (29.78)
Fee status		
Home	276 (3.00)	2,774 (30.13)
EU	15 (3.58)	217 (51.79)
International (non-EU)	12 (1.40)	401 (46.63)
Current year of study		
Year 1	21 (1.07)	645 (32.86)
Year 2	42 (1.95)	713 (33.13)
Year 3	53 (2.72)	596 (30.53)
Year 4 (not penultimate year)	46 (4.86)	326 (34.42)
Penultimate year	75 (3.77)	616 (30.97)
Final year	66 (4.45)	396 (33.45)
Total	303 (100.00)	3,392 (100.00)

Table 2 – Demographic subanalysis of students intending to leave the medical profession and of students intending to emigrate to practise medicine.

We also performed demographic subanalysis on students' likelihood to return to the UK if emigrating abroad (*Supplemental Table 5*). Males were significantly less likely to return to the UK after emigrating to practise medicine than females (p<0.00001, OR 0.65). Postgraduates were significantly less likely to return to the UK after emigrating to practise medicine than undergraduates (p<0.00001, OR 0.85). Privately educated students were significantly less likely to return to the UK after emigrating to practise medicine than state educated

students (p<0.001, OR 0.77). Non-home students (international and EU fees) were significantly less likely to return to the UK after emigrating to practise medicine than home students (p<0.00001, OR 0.18).

Reasons for students' decisions and overall view of aspects of working in the NHS.

Once students had indicated their intended career option, they were asked the importance behind each of the factors below in their decision to do so. A series of Likert scale matrices were used, with options varying from 'Very important' to 'Not at all important'. The elements used in the matrices were compiled by the authors through a review of the literature, social media, and input from other clinicians. Students' reasons for leaving the NHS, either by emigrating or leaving the profession entirely, can be found in *Figures 1a and 1b*. For those not entering either the Foundation Programme or specialty training, immediately after completion of medical school or foundation training, burnout, and the ability to choose their working location were the most important factors in this decision. The full results can be found in the *Supplemental Figures 4 and 5*.

Remuneration at junior level, work-life balance, autonomy over choice of location and the working conditions of doctors in the NHS were identified as the most important factors for students intending to emigrate to practise medicine (Figure 2a). Similarly, this was also the case for those leaving medicine, with the addition of nearly 82% of medical students listing burnout as an important or very important reason to abandon the profession (*Figure 2b*).

To better ascertain the medical student population's overview of working in the NHS, students were asked to share their degree of satisfaction with several aspects of working in the NHS. Likert scale matrices were again used in a similar fashion, with options ranging from 'Very satisfied' to 'Not at all satisfied'. *Supplemental Figure 6* highlights this. Less than 6% of the medical student population reported feeling satisfied or very satisfied with remuneration at junior level, work-life balance, working conditions of a doctor in the NHS, and costs associated with training (such as charges for memberships and examinations). A sizeable proportion of participants responded with a neutral rating, neither satisfied nor unsatisfied, when asked about several aspects of their prospective medical training. Specifically, these aspects included pension tax rules as a consultant, theatre time during the Foundation Programme, and exposure to their desired specialty during the foundation programme. In cases where participants may not have held strong opinions on a particular aspect, they tended to select the neutral option. Only 17.26% of students were satisfied or very satisfied with the overall prospect of working in the NHS.

Thematic analysis

Improving the prospect of working in the NHS

For peer review only

In total, 10,486 survey responses were collected, of which 5,294 students provided qualitative data by answering the optional question, resulting in a response rate of 50.47%. The qualitative data revealed a wide range of responses in terms of both length and content, which were subjected to thematic analysis. The majority of responses could be grouped into at least one of six distinct categories. Most of the responses alluded to concepts present in multiple categories and so were counted in all which applied. Notably, a small proportion of responses (2.08%, n=110) were deemed too broad, too vague, unintelligible, or otherwise impossible to categorise and were grouped into a separate category labelled as "Other".

"Financial considerations" were the most commonly cited area for improvement (n=4,284, 80.92%), encompassing a desire for greater remuneration, changes to pension policy, and a reduction in mandatory fees incurred by working as a doctor. A substantial proportion of those advocating for enhanced remuneration made specific reference to the concept of "pay restoration": a reversal of any real terms decline in pay faced by doctors. Concepts relating to "Working in the NHS" were also extremely prevalent (n=4,102, 77.48%), generally focusing on improved work-life balance and working conditions in the health service.

Responses to which the label of "Training and practice" was attributed (n=1,745, 32.96%) chiefly focused on autonomy of working location, with many opposing the current system of rotational training, in which doctors' working location is changed on a semi-regular basis throughout their training. This may involve regularly moving between hospital departments or to different hospitals. The issues of accessibility, quality, and streamlining – generally, shortening – of postgraduate training programmes also arose quite often. Some were dissatisfied with the presence of tasks seen to constitute "service provision" during training programmes, such as performing phlebotomy and writing patient discharge summaries, in the place of dedicated teaching or training opportunities. A number of respondents proposed changes to the current processes of applying for, and being allocated to foundation and specialty training programmes. Recurring ideas were a reduced emphasis on portfolio-building and the phasing-out of centralised, national applicant ranking methods.

In the "NHS and society" category (n=1,672, 31.58%), perceived insufficient staffing levels and a desire for increased NHS funding were the most noteworthy topics of discussion. Some described frustration with current NHS management structures and systemic inefficiencies, at times advocating for a thinning of middle-management along with a greater role for doctors and other clinicians in the system's operation. A minority suggested changes in government leadership and current political circumstances might enhance the prospect of working in the NHS. Interestingly, there were some conflicting views among our respondents, with one group backing the reversal of perceived NHS privatisation and a guarantee that it will continue to exist in its current form. The opposing, and similar sized, group promoted increasing levels of privatisation and, occasionally, introducing a fee-for-service model.

Slightly less common were responses which were grouped into the "Culture and support" category (n=994, 18.78%), in which perceptions of insufficient support, and inadequate rights, respect, and treatment of doctors

For peer review only - http://bmjopen.bmj.com/site/about/guidelines.xhtml

and allied health professionals led the argument. Those who considered the latter alluded to a sense of these roles and the individuals who fill them not being valued or shown the respect they deserve. Finally, "Medical school and education" was the category alluded to least often by our respondents (n=194, 3.66%), with primary concerns relating to university degree funding and debt, and a lack of early career guidance.

Table 3 presents the categories and their corresponding themes in full, along with the number of responses that corresponded to each theme.

Key themes generated	Number of mentions	Percentage of students
Financial considerations	*4,284*	*80.92%*
Remuneration	4,080	*77.07%*
Fees incurred by medical practice, e.g. examinations, courses	155	*2.93%*
Pension	49	*0.93%*
Working in the NHS	*4,102*	*77.48%*
Work-life balance, rotas and flexibility	*1,749*	*33.04%*
Working conditions	*1,389*	*26.24%*
Levels of stress, responsibility and pressure	*337*	*6.37%*
Breaks, leave and non-clinical opportunities	*214*	*4.04%*
Resources, equipment, technology and facilities	*205*	*3.87%*
Incentives, benefits and perks, e.g. parking, accommodation, etc	*203*	*3.83%*
Visa status and citizenship	5	*0.09%*
Training and practice	*1,745*	*32.96%*
Autonomy of working location and reduction in rotational training	*525*	*9.92%*
Levels of competition for foundation posts, specialty training posts, and consultant posts	*446*	*8.42%*
Quality of training and teaching	*282*	*5.33%*
Streamlining foundation and specialty training	*174*	*3.29%*
"Service provision", non-clinical responsibilities, and bureaucracy	*126*	*2.38%*
Postgraduate training application and allocation processes	124	*2.34%*
Regulation of AHPs* and prioritisation of doctors' training and tasks	46	*0.87%*
Variety and degree of specialty exposure before training application	22	*0.42%*
The NHS and society	*1,672*	*31.58%*
Staffing levels	850	*16.06%*
Funding of the NHS, social care, and other health services	258	*4.87%*
Operational reform, efficiency and reduction in non-clinical middle management	174	*3.29%*
Bed availability, waiting lists, appointment duration and patient experience	125	*2.36%*
Prioritising mental well-being of NHS staff	105	*1.98%*
Changes in government leadership	64	*1.21%*
Addressing discrimination in the NHS	36	*0.68%*
Reversal of privatisation of the NHS and assurance of NHS' longevity	32	*0.60%*
Increased privatision of the NHS	28	*0.53%*
Culture and support	*994*	*18.78%*
Treatment and respect for doctors and AHPs	371	*7.01%*
Support for doctors and AHPs	306	*5.78%*
Workplace culture	224	*4.23%*
Staff morale	63	*1.19%*
Autonomy of practice, litigation, and the GMC	30	*0.57%*
Medical school and education	*194*	*3.66%*
Career, portfolio and specialty application guidance	78	*1.47%*
University degree funding and debt	71	*1.34%*
Quality, design and conditions of medical school education	24	*0.45%*
Medical school places and changes to entry requirements	15	*0.28%*
Degree length and flexibility	6	*0.11%*
Other	*110*	*2.08%*
Vague, uninterpretable, or otherwise uncategorisable	*110*	*2.08%*
Total	*5,294*	*100.00%*

Table 3 – Thematic analysis of students' suggestions for improving the prospect of working in the NHS. *AHPs = allied health professionals, e.g., physician's associate and advanced clinical practitioners.

Discussion

Principal findings

Our findings highlight that a high proportion of medical students intend to either leave the profession or permanently emigrate to practise medicine. There are no previous studies to which to compare these results, so it is hard to gauge how these figures may have changed over time. We have observed that with each successive year of medical school, students become less inclined to enter specialty training in the UK without a break, or at all. Specifically, less than a quarter of final-year medical students intend to enter specialty training immediately after the Foundation Programme. 35.23% of medical students plan to leave the NHS within two years of graduating, either to practise abroad or to pursue other careers. Approximately 60% of the medical student population in the UK is either not satisfied or not at all satisfied with the prospect of working in the NHS.

Implications

The NHS is facing a critical workforce shortage, with approximately 10,000 doctors relinquishing their license to practise in 2021, representing a loss of nearly one-tenth of the doctor workforce (5, 10). A British Medical Association (BMA) survey of 8,000 senior doctors determined that 44% of NHS consultants in England plan to leave or take a break from working in the NHS over the next year (11). Similarly, a recent survey of 4,553 junior doctors in the NHS reported that 4 in 10 plan to leave the NHS, with 33% of these wanting to emigrate to another country to work (12). The combination of these previous surveys of the doctor workforce, and the findings of our medical student survey suggest this trend is presently unlikely to improve. The GMC has recognised the problem and called for immediate action to mitigate the exodus of doctors from the NHS to more attractive employers (13).

In the UK, after medical school, medical graduates enter the Foundation Programme, a two-year programme consisting of a series of 4-month or 6-month rotations through various specialties and clinical settings. The successful completion of the programme's first year (FY1) will provide doctors with full GMC registration, recognised internationally. In many cases, individuals who leave the NHS after FY1 rather than immediately following graduation may do so because of the opportunities available with the full registration provided by completion of FY1. Completion of the second year of the programme (FY2), allows applicants to apply for specialist training pathways, such as those in psychiatry, neurosurgery, and general practice (14, 15).

Countries within the anglosphere, namely Australia, New Zealand, the United States and Canada, were the most widely cited destinations for students intending to emigrate. This is perhaps unsurprising given the higher salaries, reports of improved work-life balance, and the fact that these countries' primary language is English (16). Our study's findings align with previous literature highlighting doctors' reasons for emigration (1, 17).

Our findings report that a disconcerting proportion of students, 32.35% (CI: 31.46%, 33.25%), intend to emigrate to practise medicine, with nearly half of these students intending not to return. This represents a large proportion of the medical student cohort. Despite these figures, there remains great uncertainty in this area. It

For peer review only

is important to note that a considerable number of students who initially express an intention to emigrate temporarily may ultimately choose to stay abroad permanently (17). Similarly, some students who do not intend to return to the UK may change their minds in the future. Students paying EU or international fees reported significantly higher intentions to emigrate permanently. The stage at which students intend to emigrate appears to be related to the likelihood of return. Notably, our study's findings suggest that the proportion of students who intend to leave the NHS may be underestimated, as more students express a desire to leave as they progress through medical school. Moreover, once students enter the Foundation Program, a proportion may decide to leave the NHS, even if they had not previously intended to do so.

In addition to the 35.24% of medical students intending to quit the NHS within two years of graduating, a considerable proportion of students (21.11%, CI: 20.27%, 21.98%) intend to assume a non-training clinical position in the UK after completing the Foundation Programme. Participants reported motivations for working in a non-training job in keeping with existing literature surrounding the 'F3' year, with burnout, the ability to choose work location, travel and a greater earning potential being the most compelling reasons to do so. Furthermore, in this aspect, we report a decrease in intention to take up specialty posts immediately after the Foundation Programme, with an increase from 6.75% (CI: 5.62%, 8.08%) of first-year students to 35.98% (CI: 33.45%, 38.59%) of final year students. A contributing factor to this scenario could be a significant increase in competition ratios for specialty training posts, partly due to increasing medical student places and no corresponding increase in the number of training posts available (for example, neurosurgery ST1 competition ratio was 3.9 in 2013 vs 15.94 in 2022) (18). Without corresponding increases to specialist training posts, increases in medical school places may be ineffective in doctor retention.

Historically, the vast majority of medical graduates pursued specialty training immediately after completing their Foundation Programme; for instance, in 2010, 83.1% of doctors entered specialty training after completing FY2. However, after steadily decreasing year-on-year, this percentage was only 34.9% of doctors in 2019 (14). The UK Foundation Programme Office has not repeated the survey since then, so surmising how these statistics may have changed in the interim is difficult. Our findings report that under half of medical students intend to enter specialty training after the Foundation Programme, with a negative correlation between medical student seniority and intention to enter specialty training with no break, or at all. Only 25.80% of final-year students intend to do so. In the UKFPO survey, those doctors had experienced both the positive and negative aspects of the profession. As such, it is concerning to observe this decline in interest among medical students, who have yet to formally begin their career in medicine.

Our findings suggest that the recent calls for dramatic increases in medical school places are unlikely to resolve the NHS staffing shortages. The MSC has responded to the original call to increase places by 5,000 students by stating multiple barriers, including cost, clinical placement capacity and the lack of a strategic approach to growth. It is estimated that to increase medical schools' capacity by just 5,000 places, approximately £1 billion per annum would be required (19). Additionally, the training of medical students heavily relies on clinical

For peer review only

exposure, which in turn is dependent on availability of clinical teaching staff, facilities for training and opportunities (6). Without a corresponding increase in clinical placement capacity, an increase in medical student places may result in a decrease in the standard of medical education. Our results indicate that increases in medical student places via expansion of existing medical schools or the establishment of new medical schools may not result in proportionate increases in doctors wishing to remain in the NHS. Any attempts to reverse the NHS workforce challenge may benefit from prioritising doctor retention. Here we have highlighted the reasons driving medical students to plan for careers outside of the NHS; addressing these problems is likely to result in improved retention rates.

Whilst there have been studies which i) explore which specialties junior doctors or medical students intend on pursuing, and exploring factors attracting them to said specialties (20-42); ii) focus on reasons why doctors are leaving the UK(43, 44); iii) exploring how medical students and junior doctors feel about specific aspects of working within the NHS (45-48), and iv) studies investigating the desire for a career break post-FY2 (49-51), there have been no recent, high-powered studies explicitly aimed at medical students, irrespective of current career ambitions or seniority, investigating overall career intentions and correlating it with demographic factors and medical student seniority. Any statistically significant differences in career intentions between demographic subgroups should be considered carefully and discussed in the correct context. Further studies are required to fully elucidate the reasons behind these discrepancies.

Limitations

When interpreting this study's results, there are important limitations to consider. Firstly, the study's cross-sectional nature means we are unable to gauge how students' career intentions may have changed or will change. To address this, we have asked all participants for consent to participate in an anticipated follow-up study which will enable validation of responses and measurement of change over time; to this, we obtained a 71.29% consent rate. We cannot exclude the possibility of selection bias, despite being the largest UK medical student survey of all time, only 21.50% of medical students participated. It may be that this survey appealed to those already intending to leave the NHS or are interested in this topic. In the context of the UK's medical student population, females were overrepresented in our study. While efforts were made to reduce bias in the interpretation of the study's qualitative data, inherent subjectivity remains a limitation.

Additionally, the questions in our survey instruct students to be definitive even when they might not yet have an idea of their career plans, particularly for those in the younger years of medical school. Finally, the majority of respondents to the survey were medical students who may have limited knowledge of the realities of working in the NHS. Their current reported perceptions may change once they begin their career in the NHS.

Conclusion

For peer review only

This study highlights that an alarming proportion of medical students intend to leave the profession or emigrate to practise medicine. The proportion of students who plan to leave the NHS within two years of graduating is considerable, representing a potential loss of valuable medical talent. Alarmingly, the majority of medical students are either not at all satisfied or not satisfied with the prospect of working in the NHS. Additionally, an increasing proportion of students intend to take up non-training clinical positions, which could reduce the availability of highly skilled doctors in the NHS. The findings of this study underscore the urgency of addressing the factors that are driving the exodus of doctors from the NHS and suggest that increased recruitment of medical students may not provide an adequate solution to the staffing challenges. The causes of the problem are complex, and finding a solution will require a multifaceted approach. Steps could include improving work-life balance, increasing salaries, addressing the growing competition for training posts, and promoting greater flexibility in career pathways. Undoubtedly, the continued loss of skilled professionals from the NHS represents a significant concern, so it is critical to consider means of reversing this trend.

What is already known on this topic

There are several factors behind doctors' motivations to emigrate to practise medicine or leave the profession entirely. Commonly cited themes include pay erosion and low pay compared to alternative destinations, working conditions within the NHS, well-being, work-life balance, and better training opportunities abroad. Although studies have explored the reasons why doctors consider careers outside of the NHS, research is yet to be carried out on medical students' career intentions, irrespective of their current specialty of choice, and correlating those with seniority or demographic factors.

What this study adds

To the best of our knowledge, this is the largest ever UK study of medical students, having received responses from students in all UK medical schools recognised by the MSC. The mixed-methods nature of the study and high engagement with all aspects of the survey facilitates a richer understanding of the issue. We have characterised the medical student population, the proportion of students intending to pursue specific career paths and their reasons for doing so. It has been revealed that a concerning number of medical students intend to leave the NHS within two years of graduating, representing a potential loss of valuable medical talent. Furthermore, a notable proportion of medical students intend to assume non-training clinical roles, potentially resulting in a scarcity of highly skilled doctors in the NHS.

For peer review only

Notes

Authors' contributions

TF responsible for conceptualisation. TF responsible for obtaining funding and ethical approval. TF responsible for collaborator recruitment and management. TF responsible for project administration. TF and AMC responsible for designing the survey. TF responsible for writing the manuscript. AMC responsible for qualitative analysis. OF and RJS responsible for statistical quantitative analysis. All authors responsible for editing and revising the manuscript. RH responsible for supervision. TF is the guarantor. All authors have read and approved the manuscript.

Collaborators

Mario K Teo, Crispin C Wigfield, Dania Al-Hashimi, Maeve K Mulchrone, Alisha Pervaiz, Heather A Lewis, Anson Wong, Buzz Gilks, Charlotte Casteleyn, Sara Kidher, Erin Fitzsimons-West, Tanzil Rujeedawa, Meghna Sreekumar, Eliza Wade, Juel Choppy-Madeleine, Yasemin Durmus, Olivia King, Yu Ning Ooi, Malvi Shah, Tan Jih Yih, Samantha Burley, Basma R Khan, Emma Slack, Rishik S Pilla, Jenny Yang, Vaishvi Dalal, Brennan L Gibson, Emma Westwood, Brandon S H Low, Sara R Sabur , Wentin Chen, Maryam A Malik, Safa Razzaq, Amardeep Sidki, Giulia Cianci, Felicity Greenfield, Sajad Hussain, Alexandra Thomas, Annie Harrison, Hugo Bernie, Luke Dcaccia, Linnuel J Pregil, Olivia Rowe, Ananya Jain, Gregory K Anyaegbunam, Syed Z Jafri, Arthur Handscomb, Sudhanvita Arun, Alfaiya Hashmi, Ankith Pandian, Joseph R Nicholson, Hannah Layton-Joyce, Kouther Mohsin, Matilda Gardener, Eunice C Y Kwan, Emily R Finbow, Sakshi Roy, Zoe M Constantinou, Mackenzie Garlick, Clare L Carney, Samantha Gold, Bilal Qureshi, Daniel Magee, Grace Annetts, Khyatee Shah, Kholood T Munir, Timothy Neill, Gurpreet K Atwal, Anesu Kusosa, Anthony Vijayanathan, Mia Mäntylä, Momina Iqbal, Sara Raja, Tushar Rakhecha, Muhammad H Shah, Pranjil Pokharel, Ashna Anil, Kate Stenning, Katie Appleton, Keerthana Uthayakumar, Rajan Panacer, Yasmin Owadally, Dilaxiha Rajendran, Harsh S Modalavalasa, Marta M Komosa, Morea Turjaka, Sruthi Saravanan, Amelia Dickson, Jack M Read, Georgina Cooper, Wing Chi Do, Chiamaka Anthony-Okeke, Daria M Bageac, David C

For peer review only

W Loh, Rida Khan, Ruth Omenyo, Aidan Baker, Imogen Milner, Kavyesh Vivek, Manon Everard, Wajiha Rahman, Denis Chen, Michael E. Bryan, Shama Maliha, Vera Onongaya, Amber Dhoot, Catherine L Otoibhi, Harry Donkin-Everton, Mia K Whelan, Claudia S F Hobson, Anthony Haynes, Joshua Bayes-Green, Mariam S Malik, Subanki Srisakthivel, Sophie Kidd, Alan Saji, Govind Dhillon, Muhammed Asif, Riya Patel, Jessica L Marshall, Nain T Raja, Tawfique Rizwan, Aleksandra Dunin-Borkowska, James Brawn, Karthig Thillaivasan, Zainah Sindhoo, Ayeza Akhtar, Emma Hitchcock, Kelly Fletcher, Lok Pong Cheng, Medha Pillaai, Sakshi Garg, Wajahat Khan, Ben Sweeney, Ria Bhatt, Madison Slight, Adan M I Chew, Cameron Thurlow, Kriti Yadav, Niranjan Rajesh, Nathan-Dhruv Mistry, Alyssa Weissman, Juan F E Jaramillo, William Thompson, Gregor W Abercromby, Emily Gaskin, Chloe Milton, Matthew Kokkat, Momina Hussain, Nana A. Ohene-Darkoh, Syeda T Islam, Anushruti Yadav, Eve Richings, Samuel Foxcroft, Sukhdev Singh, Vivek Sivadev, Guilherme Movio, Ellena Leigh, Harriet Charlton, James A Cairn, Julia Shaaban , Leah Njenje, Mark J Bishop, Humairaa Ismail, Sarah L Henderson, Daniel C Chalk, Daniel J Mckenna, Fizah Hasan, Kanishka Saxena , Iona E Gibson & Saad Dosani.

Transparency declaration

TF, the lead author (the manuscript's guarantor), affirms that the manuscript is an honest, accurate, and transparent account of the study being reported; that no important aspects of the study have been omitted; and that any discrepancies from the study have been explained.

Ethics approval and consent to participate.

Ethical approval was granted by the University of Cambridge Research Ethics Committee (reference PRE.2022.124) on the 5th of January 2023.

Consent for publication

The Corresponding Author has the right to grant on behalf of all authors and does grant on behalf of all authors, an exclusive licence (or non-exclusive for government employees) on a worldwide basis to the BMJ Publishing Group Ltd to permit this article (if accepted) to be published in BMJ editions and any other BMJPGL products and sublicences such use and exploit all subsidiary rights, as set out in our licence.

Competing interests

All authors have completed the Unified Competing Interest form (available on request from the corresponding author) and declare: no support from any organisation for the submitted work; no financial relationships with any organisations that might have an interest in the submitted work in the previous three years, no other relationships or activities that could appear to have influenced the submitted work.

Funding

Queens' College, University of Cambridge. The institution has had no role in the design of the study, nor collection, analysis, and interpretation of data and in writing the manuscript.

For peer review only

Patient and public involvement

No members of the public were directly involved in the design or analysis of the reported data.

Acknowledgements

We would like to thank all students that participated in this study. We would also like to thank Mr Mario K Teo and Mr Crispin C Wigfield for their advice in the earlier stages of the study.

Authors' information (optional)

Not applicable.

Data availability

No additional data available.

For peer review only

Supplemental Materials

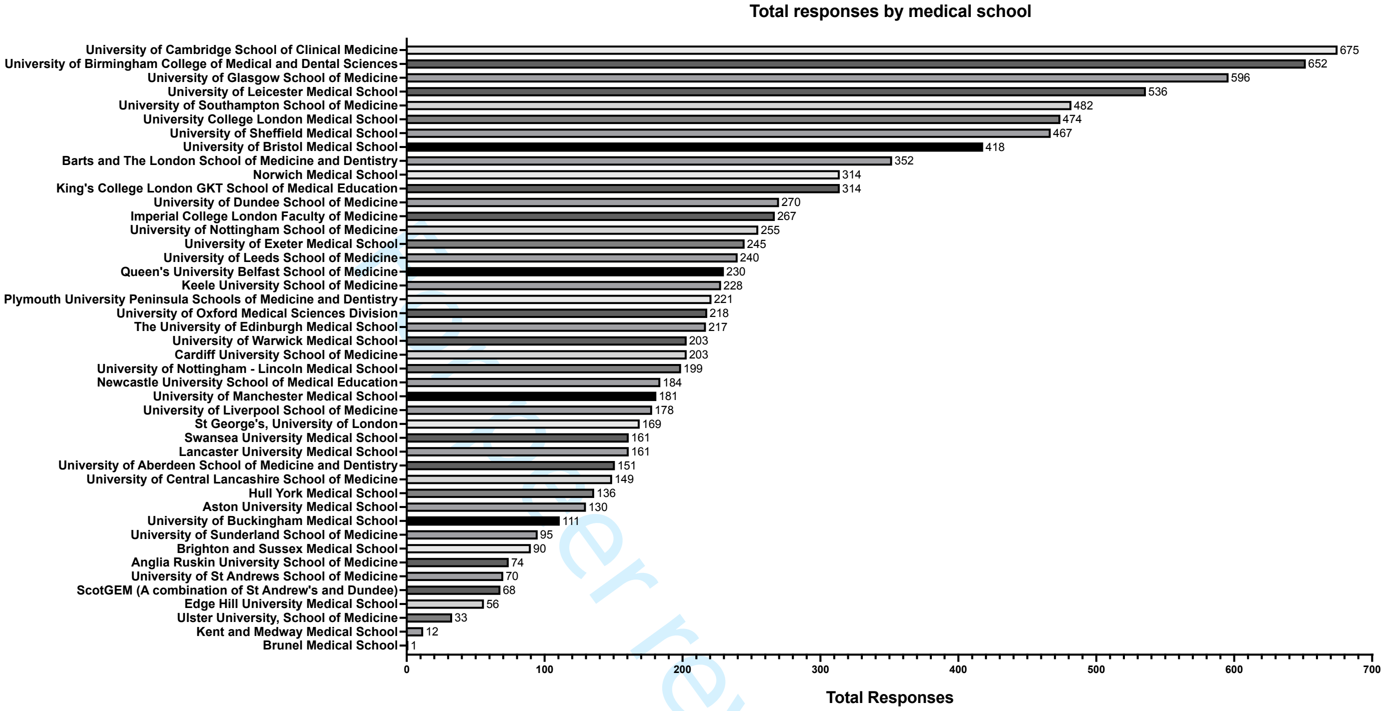

Supplemental Figure 1 - Number of eligible survey responses obtained from medical students at each of the UK's 44 medical schools recognised by the General Medical Council.

Students' intention after graduation	Number (%)	Confidence Interval
Complete both FY1 and FY2	8,806 (83.98)	[83.26, 84.67]
Complete FY1 and emigrate to practice medicine	1,101 (10.50)	[9.93,11.10]
Complete FY1 and leave medicine permanently	132 (1.26)	[1.06, 1.49]
Leave medicine permanently	104 (0.99)	[0.82, 1.20]
Emigrate to practice medicine	220 (2.10)	[1.84, 2.39]
Take a break or undertake further study	123 (1.17)	[0.98, 1.40]

Supplemental Table 1 – Career intentions of participants after graduation

Students' intention after the Foundation Programme	Number (%)	Confidence Interval
Enter specialty training in the UK	4,294 (48.76)	[47.72, 49.81]
Assume a non-training clinical job in the UK	1,859 (21.11)	[20.27, 21.98]
Emigrate to practice medicine abroad (including temporarily)	2,071 (23.52)	[22.64, 24.42]
Take a break or undertake further study	515 (5.85)	[5.38, 6.36]
Leave medicine permanently	67 (0.76)	[0.60, 0.97]

Supplemental Table 2 – Career intentions of participants after the Foundation Programme.

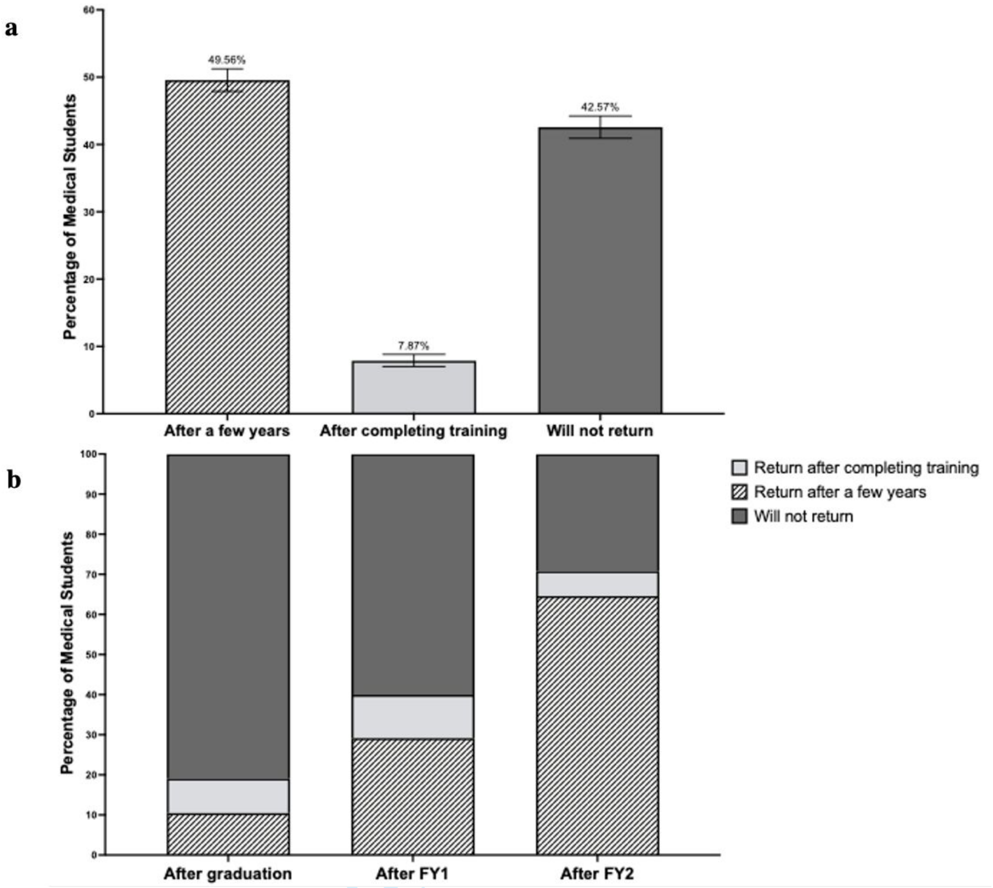

Supplemental Figures 2a, b - a) Return prospects of students intending to emigrate (with 95% confidence intervals) **b)** return prospects of students intending to emigrate by stage at which they intend to emigrate.

Students' intention after graduation	Year 1	Year 2	Year 3	Year 4 (not penultimate year)	Penultimate Year	Final Year
Complete both FY1 and FY2	1616 (82.32)	1723 (80.07)	1643 (84.17)	759 (80.15)	1728 (86.88)	1337 (90.16)
Complete FY1 and emigrate to practice medicine	251 (12.79)	283 (13.15)	208 (10.66)	114 (12.04)	161 (8.09)	84 (5.66)
Complete FY1 and leave medicine permanently	8 (0.41)	15 (0.70)	24 (1.23)	18 (1.90)	37 (1.86)	30 (2.02)
Leave medicine permanently	10 (0.51)	23 (1.07)	17 (0.87)	20 (2.11)	23 (1.16)	11 (0.74)
Emigrate to practice medicine	61 (3.11)	65 (3.02)	45 (2.31)	19 (2.01)	20 (1.01)	10 (0.67)
Take a break or undertake further study	17 (0.87)	43 (2.00)	15 (0.77)	17 (1.80)	20 (1.01)	11 (0.74)

Supplemental Table 3 – Career intentions after graduation, by year group.

Students' intention after the Foundation Programme	Year 1	Year 2	Year 3	Year 4 (not penultimate year)	Penultimate Year	Final Year
Enter specialty training in the UK	1093 (67.64)	1071 (62.16)	866 (52.71)	298 (39.26)	621 (35.94)	345 (25.80)
Assume a non-training clinical job in the UK	109 (6.75)	197 (11.43)	326 (19.84)	192 (25.30)	554 (32.06)	481 (35.98)
Emigrate to practice medicine abroad (including temporarily)	333 (20.61)	365 (21.18)	343 (20.88)	193 (25.43)	435 (25.17)	402 (30.07)
Take a break or undertake further study	78 (4.83)	86 (4.99)	96 (5.84)	68 (8.96)	103 (5.96)	84 (6.28)
Leave medicine permanently	3 (0.19)	4 (0.23)	12 (0.73)	8 (1.05)	15 (0.87)	25 (1.87)

Supplemental Table 4 – Career intentions after the Foundation Programme, by year group. This figure highlights the career intentions of students *after* the Foundation Programme as a percentage of those in each year group intending to enter the Foundation Programme.

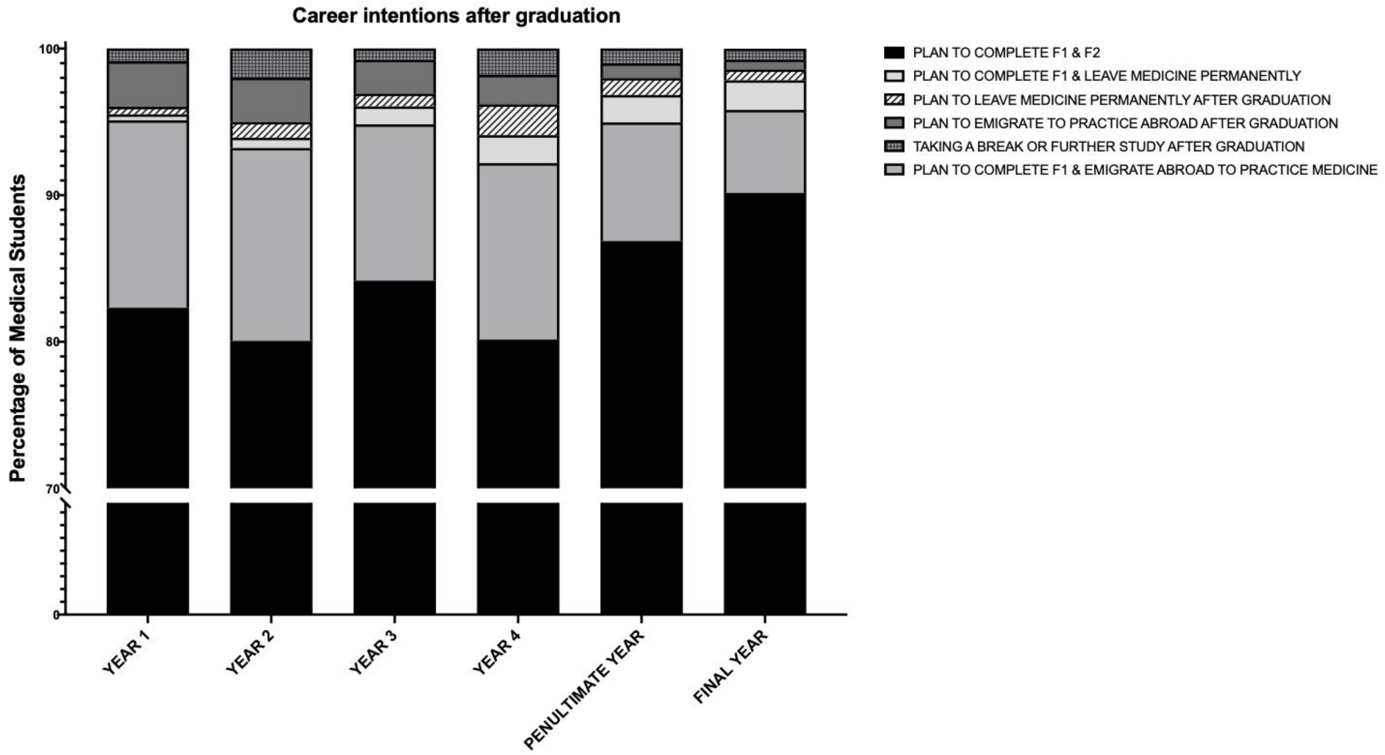

Supplemental Figure 3 – Career intentions of medical students immediately after graduation from medical school, by year of study. “Year 4” represents students in their fourth year of study, but not their penultimate year.

For peer review only

Demographic subgroup	Return prospects		
	After a few years	After completing training	Will not return
Ethnicity			
White	1,133 (58.46)	131 (6.76)	674 (34.78)
Asian or Asian British	334 (36.66)	78 (8.56)	499 (54.77)
Black, Black British, Caribbean or African	79 (44.89)	15 (8.52)	82 (46.59)
Mixed or multiple ethnic groups	88 (46.07)	23 (12.04)	80 (41.88)
Other	40 (28.37)	18 (12.77)	83 (58.87)
Prefer not to say	7 (20.00)	2 (5.71)	26 (74.29)
Gender			
Female	1,165 (53.37)	175 (8.02)	843 (38.62)
Male	512 (42.99)	92 (7.72)	587 (49.29)
Non-binary	3 (25.00)	0 (0)	9 (75.00)
Prefer not to say	1 (16.67)	0 (0)	5 (83.33)
Level of education			
Postgraduate	311 (46.49)	51 (7.62)	307 (45.89)
Undergraduate	1,370 (50.31)	216 (7.93)	1137 (41.76)
Previous schooling			
Private education	578 (44.91)	113 (8.78)	596 (46.31)
State education	1,072 (52.96)	143 (7.07)	809 (39.97)
Prefer not to say	31 (38.27)	11 (13.58)	39 (48.15)
Fee status			
Home	1,572 (56.67)	221 (7.97)	981 (35.36)
EU	45 (20.74)	17 (7.83)	155 (71.43)
International (Non-EU)	64 (15.96)	29 (7.23)	308 (76.81)
Current year of study			
Year 1	297 (46.05)	65 (10.08)	283 (43.88)
Year 2	346 (48.53)	55 (7.71)	312 (43.76)
Year 3	281 (47.15)	55 (9.23)	260 (43.62)
Year 4 (not penultimate year)	163 (50.00)	25 (7.67)	138 (42.33)
Penultimate year	313 (50.81)	47 (7.63)	256 (41.56)
Final year	281 (56.65)	20 (4.03)	195 (39.31)
Total	1681 (49.56)	267 (7.87)	1444 (42.57)

Supplemental Table 5 – Demographic subanalysis of the return prospects of students intending to emigrate to practise medicine. Data in this table includes both the absolute number of students from each subgroup, and the relative proportion of their subgroup return prospects.

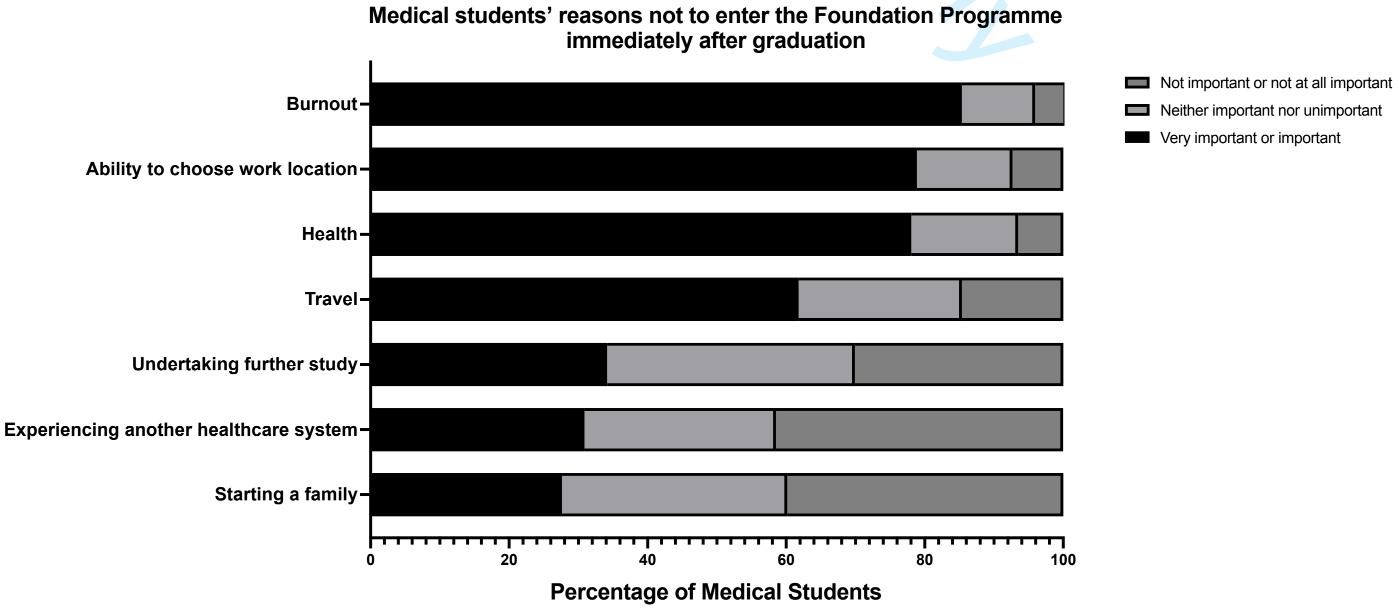

Supplemental Figure 4 - Importance of factors influencing medical students' intention not to enter the Foundation Programme immediately after their graduation.

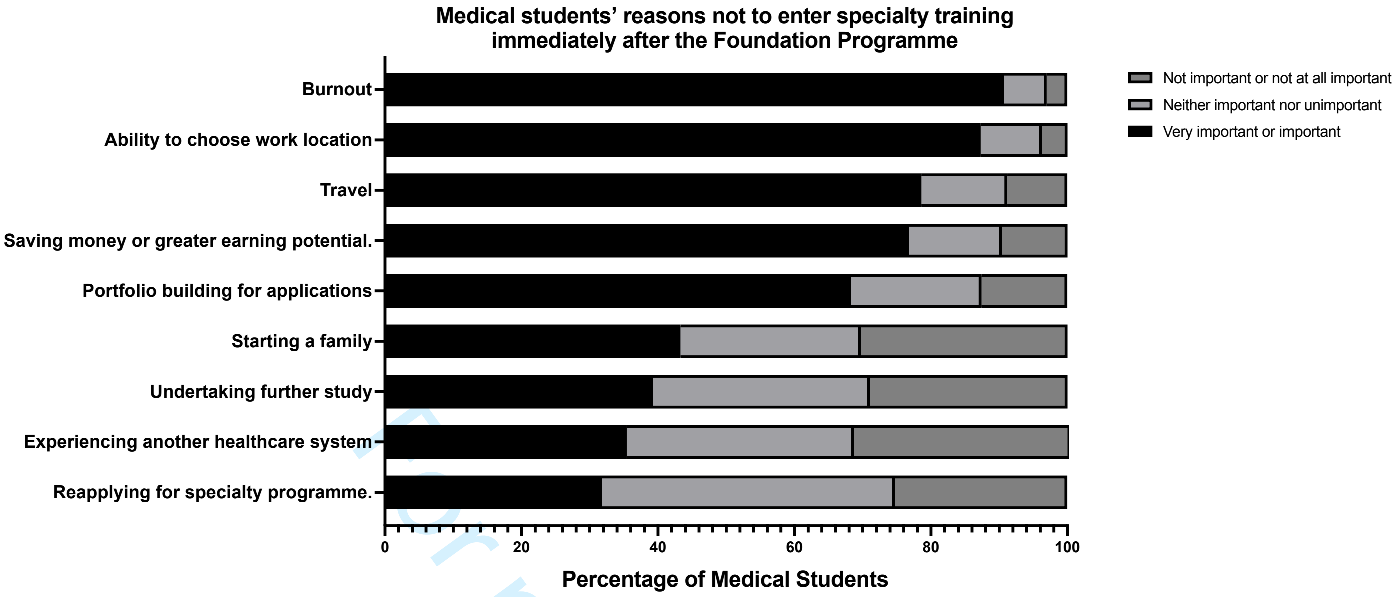

Supplemental Figure 5 - Importance of factors influencing medical students' intention not to enter specialty training immediately after their planned completion of the Foundation Programme.

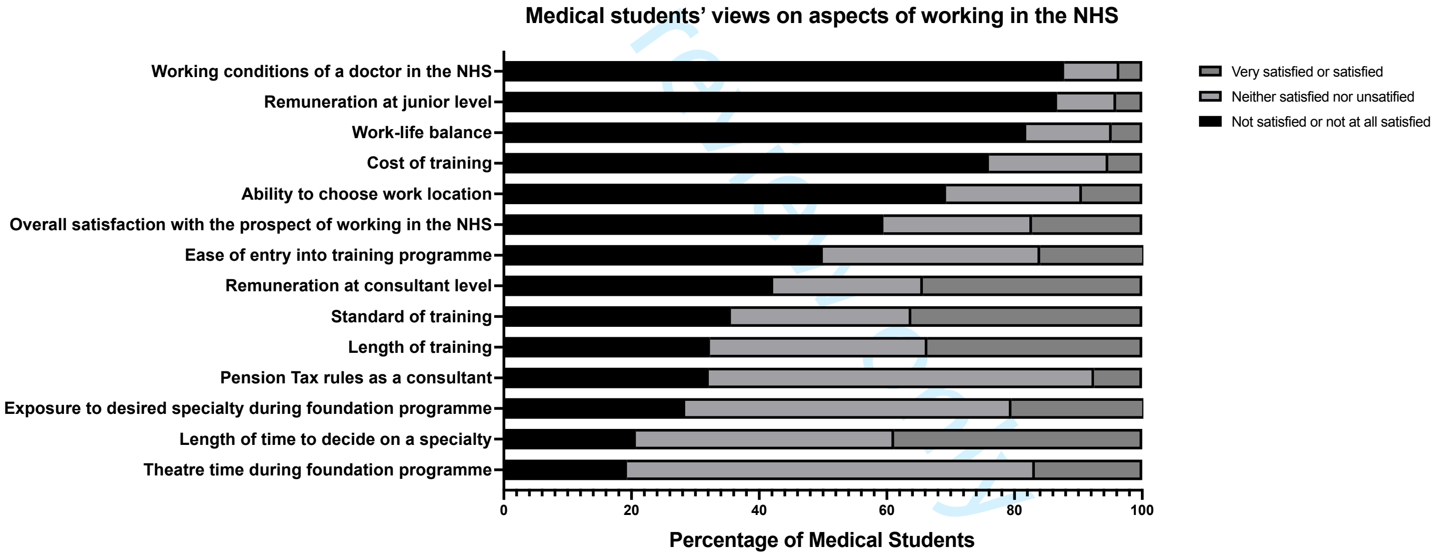

Supplemental Figure 6 – Medical students' satisfaction levels regarding aspects of working as a doctor in the NHS

Supplemental Material 1 – PDF Version of Survey

Supplemental Material 2 – List of approved MSC schools

Supplemental Material 3 – Participant Information Sheet

Bibliography

1. Lambert TW, Smith F, Goldacre MJ. Why doctors consider leaving UK medicine: qualitative analysis of comments from questionnaire surveys three years after graduation. Journal of the Royal Society of Medicine. 2018;111(1):18-30.
2. Surman G, Goldacre MJ, Lambert TW. UK-trained junior doctors' intentions to work in UK medicine: questionnaire surveys, three years after graduation. Journal of the Royal Society of Medicine. 2017;110(12):493-500.
3. OECD (2023), Doctors (indicator). doi: 10.1787/4355e1ec-en (Accessed on 15 April 2023), Available from: https://data.oecd.org/healthres/doctors.htm
4. Rimmer A. Five medical schools are created in England in bid to increase home grown doctors. BMJ: British Medical Journal (Online). 2018;360.
5. General Medical Council. The state of medical education and practice in the UK. The workforce report 2022.
6. Phillips S MI. A roadmap to double medical school places. Dec 2022.
7. Ferreira T, Collins AM, Horvath R. Study protocol - Ascertaining the career Intentions of UK Medical Students (AIMS) post-graduation: a cross-sectional survey. medRxiv. 2023:2023.01.16.23284605.
8. Elm Ev. The Strengthening the Reporting of Observational Studies in Epidemiology (STROBE) statement: guidelines for reporting observational studies. Ann Inter Med. 2007;147:573-7.
9. Braun V, Clarke V. Using thematic analysis in psychology. Qualitative research in psychology. 2006;3(2):77-101.
10. NHS Digital Health. NHS Workforce Statistics, December 2021 Doctors by Grade and Specialty. 2022.
11. BMA. Catastrophic crisis facing NHS as nearly half of hospital consultants plan to leave in next year, warns BMA2022. Available from: https://www.bma.org.uk/bma-media-centre/catastrophic-crisis-facing-nhs-as-nearly-half-of-hospital-consultants-plan-to-leave-in-next-year-warns-bma.
12. Waters A. A third of junior doctors plan to leave NHS to work abroad in next 12 months. British Medical Journal Publishing Group; 2022.
13. General Medical Council. Migration and the medical workforce 2022 [Available from: https://www.gmc-uk.org/news/news-archive/migration-and-the-medical-workforce.
14. UKFPO. UK Foundation Programme 2019 F2 Career UKFPO 2019 F2 Career Destinations Survey. 2019.
15. De Souza B. Choosing your specialty Foundation training. BMJ. 2007;334(7601):s172-s3.
16. Grant P. Physician job satisfaction in New Zealand versus the United Kingdom. The New Zealand Medical Journal (Online). 2004;117(1204).
17. Sharma A, Lambert TW, Goldacre MJ. Why UK-trained doctors leave the UK: cross-sectional survey of doctors in New Zealand. Journal of the royal society of medicine. 2012;105(1):25-34.
18. NHS Health Education England. Competition Ratios [Available from: https://medical.hee.nhs.uk/medical-training-recruitment/medical-specialty-training/competition-ratios.
19. Medical Schools Council. The expansion of medical student numbers in the United Kingdom. 2021.
20. Singh A, Alberti H. Why UK medical students change career preferences: an interview study. Perspectives on medical education. 2021;10(1):41-9.

21. Misky AT, Shah RJ, Fung CY, Sam AH, Meeran K, Kingsbury M, et al. Understanding concepts of generalism and specialism amongst medical students at a research-intensive London medical school. BMC Medical Education. 2022;22(1):1-11.
22. Lambert TW, Smith F, Goldacre MJ. Career specialty choices of UK medical graduates of 2015 compared with earlier cohorts: questionnaire surveys. Postgraduate Medical Journal. 2018;94(1110):191-7.
23. Surman G, Lambert TW, Goldacre MJ. Trends in junior doctors' certainty about their career choice of eventual clinical specialty: UK surveys. Postgraduate medical journal. 2013;89(1057):632-7.
24. Svirko E, Goldacre MJ, Lambert T. Career choices of the United Kingdom medical graduates of 2005, 2008 and 2009: questionnaire surveys. Medical teacher. 2013;35(5):365-75.
25. Ibrahim M, Fanshawe A, Patel V, Goswami K, Chilvers G, Ting M, et al. What factors influence British medical students' career intentions? Medical teacher. 2014;36(12):1064-72.
26. Reid K, Alberti H. Medical students' perceptions of general practice as a career; a phenomenological study using socialisation theory. Education for Primary Care. 2018;29(4):208-14.
27. Rehman U, Sarwar MS, Brennan PA. Attitude of clinical medical students to Oral and Maxillofacial Surgery as a career: a perspective from two English Medical Schools. British Journal of Oral and Maxillofacial Surgery. 2022;60(4):448-53.
28. Barber S, Brettell R, Perera-Salazar R, Greenhalgh T, Harrington R. UK medical students' attitudes towards their future careers and general practice: a cross-sectional survey and qualitative analysis of an Oxford cohort. BMC medical education. 2018;18(1):1-9.
29. Oliver H, Hudson B, Oliver C, Oliver M. UK undergraduate aspirations and attitudes survey: do we have a perception problem in clinical radiology? Clinical Radiology. 2020;75(2):158. e15-. e24.
30. Emmanouil B, Goldacre MJ, Lambert TW. Aspirations to become an anaesthetist: longitudinal study of historical trends and trajectories of UK-qualified doctors' early career choices and of factors that have influenced their choices. BMC anesthesiology. 2017;17(1):1-9.
31. Barat A, Goldacre MJ, Lambert TW. Junior doctors' early career choices do not predict career destination in neurology: 40 years of surveys of UK medical graduates. BMC medical education. 2019;19(1):1-9.
32. Robinson T, Lefroy J. How do medical students' experiences inform their opinions of general practice and its potential as a future career choice? Education for Primary Care. 2022:1-9.
33. Tambyraja AL, McCrea CA, Parks RW, Garden OJ. Attitudes of medical students toward careers in general surgery. World journal of surgery. 2008;32(6):960-3.
34. Thomas A. What about forensic psychiatry as a career? Undergraduate and early post-graduate medical perspectives. Criminal Behaviour and Mental Health. 2012;22(4):247-51.
35. Smith F, Lambert TW, Pitcher A, Goldacre MJ. Career choices for cardiology: cohort studies of UK medical graduates. BMC medical education. 2013;13(1):1-8.
36. Maisonneuve JJ, Pulford C, Lambert TW, Goldacre MJ. Career choices for geriatric medicine: national surveys of graduates of 1974–2009 from all UK medical schools. Age and ageing. 2014;43(4):535-41.
37. Goodson AM, Payne KF, Tahim A, Cabot L, Fan K. Awareness of oral and maxillofacial surgery as a specialty and potential career pathway amongst UK medical undergraduates. The Surgeon. 2013;11(2):92-5.
38. Halder N, Hadjidemetriou C, Pearson R, Farooq K, Lydall GJ, Malik A, et al. Student career choice in psychiatry: findings from 18 UK medical schools. International Review of Psychiatry. 2013;25(4):438-44.
39. Goldacre MJ, Fazel S, Smith F, Lambert T. Choice and rejection of psychiatry as a career: surveys of UK medical graduates from 1974 to 2009. The British Journal of Psychiatry. 2013;202(3):228-34.
40. Pakpoor J, Handel AE, Disanto G, Davenport RJ, Giovannoni G, Ramagopalan SV. National survey of UK medical students on the perception of neurology. BMC medical education. 2014;14(1):1-5.
41. Sutton PA, Mason J, Vimalachandran D, McNally S. Attitudes, motivators, and barriers to a career in surgery: a national study of UK undergraduate medical students. Journal of surgical education. 2014;71(5):662-7.
42. Moore J, McDiarmid A, Johnston P, Cleland J. Identifying and exploring factors influencing career choice, recruitment and retention of anaesthesia trainees in the UK. Postgraduate medical journal. 2017;93(1096):61-6.
43. Wilson HC, Abrams S, Simpkin Begin A. Drexit: Understanding why junior doctors leave their training programs to train overseas: An observational study of UK physicians. Health Science Reports. 2021;4(4):e419.
44. Milner A, Nielsen R, Verdery AM. Brexit and the European National Health Service England Workforce: A Quantitative Analysis of Doctors' Perceived Professional Impact and Intentions to Leave the United Kingdom. Annals of global health. 2021;87(1).
45. Scanlan GM, Cleland J, Johnston P, Walker K, Krucien N, Skåtun D. What factors are critical to attracting NHS foundation doctors into specialty or core training? A discrete choice experiment. BMJ open. 2018;8(3):e019911.

For peer review only

46. Ryan C, Ward E, Jones M. Recruitment and retention of trainee physicians: a retrospective analysis of the motivations and influences on career choice of trainee physicians. QJM: An International Journal of Medicine. 2018;111(5):313-8.
47. Lachish S, Goldacre MJ, Lambert T. Associations between perceived institutional support, job enjoyment, and intentions to work in the United Kingdom: national questionnaire survey of first year doctors. BMC medical education. 2016;16(1):1-8.
48. Cleland JA, Johnston P, Watson V, Krucien N, Skåtun D. What do UK medical students value most in their careers? A discrete choice experiment. Medical Education. 2017;51(8):839-51.
49. Church HR, Agius SJ. The F3 phenomenon: Early-career training breaks in medical training. A scoping review. Medical Education. 2021;55(9):1033-46.
50. Hollis AC, Streeter J, Van Hamel C, Milburn L, Alberti H. The new cultural norm: reasons why UK foundation doctors are choosing not to go straight into speciality training. BMC Medical Education. 2020;20(1):1-9.
51. Agius SJ, Tack G, Murphy P, Holmes S, Hayden J. Why do medical trainees take time out of their specialty training programmes? British Journal of Hospital Medicine. 2014;75(10):584-9.

For peer review only

**Characteristic**	**Number (%)**
Ethnicity	
White	5,838 (55.67)
Asian or Asian British	3,027 (28.87)
Black, Black British, Caribbean or African	529 (5.04)
Mixed or multiple ethnic groups	555 (5.29)
Other	410 (3.91)
Prefer not to say	127 (1.21)
Gender	
Female	6,977 (66.54)
Male	3,429 (32.70)
Non-binary	64 (0.61)
Prefer not to say	16 (0.15)
Level of education	
Postgraduate	1,873 (17.86)
Undergraduate	8,613 (82.14)
Previous schooling	
Private education	3,605 (34.38)
State education	6,609 (63.03)
Prefer not to say	272 (2.59)
Fee status	
Home	9,207 (87.80)
EU	419 (4.00)
International (Non-EU)	860 (8.20)
Current year of study	
Year 1	1,963 (18.72)
Year 2	2,152 (20.52)
Year 3	1,952 (18.62)
Year 4 (not penultimate year)	947 (9.03)
Penultimate Year	1,989 (18.97)
Final Year	1,483 (14.14)
Age	
Median (range)	22 (17-48)
Total	10,486 (100.00)

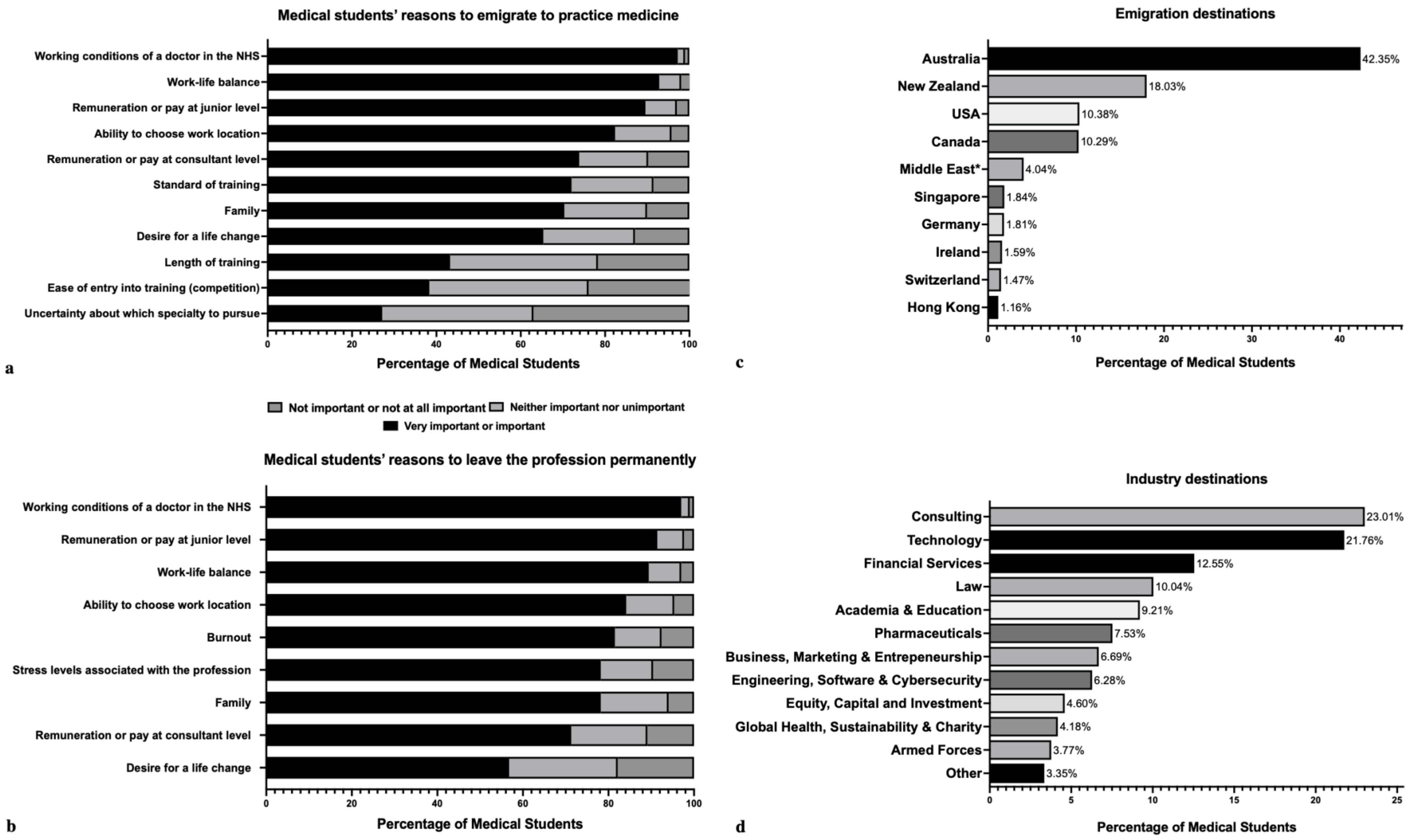
Medical students' reasons to emigrate to practice medicine
Working conditions of a doctor in the NHS
Work-life balance
Remuneration or pay at junior level
Ability to choose work location
Remuneration or pay at consultant level
Standard of training
Family
Desire for a life change
Length of training
Ease of entry into training (competition)
Uncertainty about which specialty to pursue
0
20
40
60
80
100
Percentage of Medical Students
a
Not important or not at all important
Neither important nor unimportant
Very important or important
Medical students' reasons to leave the profession permanently
Working conditions of a doctor in the NHS
Remuneration or pay at junior level
Work-life balance
Ability to choose work location
Burnout
Stress levels associated with the profession
Family
Remuneration or pay at consultant level
Desire for a life change
0
20
40
60
80
100
Percentage of Medical Students
b
Emigration destinations
Australia 42.35%
New Zealand 18.03%
USA 10.38%
Canada 10.29%
Middle East* 4.04%
Singapore 1.84%
Germany 1.81%
Ireland 1.59%
Switzerland 1.47%
Hong Kong 1.16%
0
10
20
30
40
Percentage of Medical Students
c
Industry destinations
Consulting 23.01%
Technology 21.76%
Financial Services 12.55%
Law 10.04%
Academia & Education 9.21%
Pharmaceuticals 7.53%
Business, Marketing & Entrepeneurship 6.69%
Engineering, Software & Cybersecurity 6.28%
Equity, Capital and Investment 4.60%
Global Health, Sustainability & Charity 4.18%
Armed Forces 3.77%
Other 3.35%
0
5
10
15
20
25
Percentage of Medical Students
d

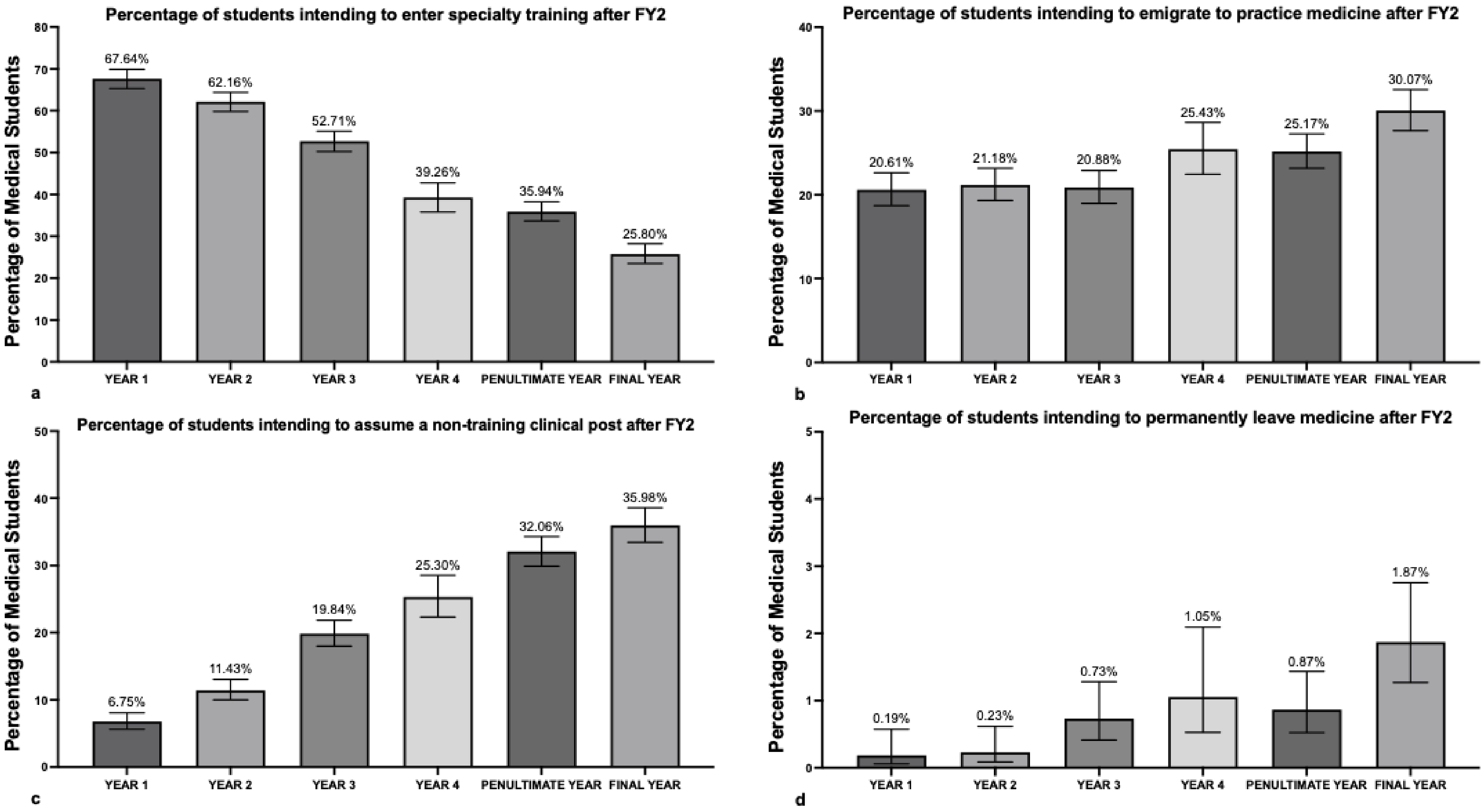
Percentage of students intending to enter specialty training after FY2
Percentage of Medical Students
67.64%
62.16%
52.71%
39.26%
35.94%
25.80%
YEAR 1
YEAR 2
YEAR 3
YEAR 4
PENULTIMATE YEAR
FINAL YEAR
a
Percentage of students intending to emigrate to practice medicine after FY2
Percentage of Medical Students
20.61%
21.18%
20.88%
25.43%
25.17%
30.07%
YEAR 1
YEAR 2
YEAR 3
YEAR 4
PENULTIMATE YEAR
FINAL YEAR
b
Percentage of students intending to assume a non-training clinical post after FY2
Percentage of Medical Students
6.75%
11.43%
19.84%
25.30%
32.06%
35.98%
YEAR 1
YEAR 2
YEAR 3
YEAR 4
PENULTIMATE YEAR
FINAL YEAR
c
Percentage of students intending to permanently leave medicine after FY2
Percentage of Medical Students
0.19%
0.23%
0.73%
1.05%
0.87%
1.87%
YEAR 1
YEAR 2
YEAR 3
YEAR 4
PENULTIMATE YEAR
FINAL YEAR
d

Demographic subgroup	Number intenting to leave medicine (%)	Number intenting to emigrate (%)
Ethnicity		
White	147 (2.52)	1,938 (33.20)
Asian or Asian British	99 (3.27)	911 (30.10)
Black, Black British, Caribbean or African	15 (2.84)	176 (33.27)
Mixed or multiple ethnic groups	24 (4.32)	191 (34.41)
Other	10 (2.44)	141 (34.39)
Prefer not to say	8 (6.30)	35 (27.56)
Gender		
Female	134 (1.92)	2,183 (31.29)
Male	167 (4.87)	1,191 (34.73)
Non-binary	1 (1.56)	12 (18.75)
Prefer not to say	1 (6.25)	6 (37.50)
Level of education		
Postgraduate	44 (2.35)	669 (35.72)
Undergraduate	259 (3.01)	2,723 (31.62)
Previous schooling		
Private education	118 (3.27)	1,287 (35.70)
State education	170 (2.57)	2,024 (30.62)
Prefer not to say	15 (5.51)	81 (29.78)
Fee status		
Home	276 (3.00)	2,774 (30.13)
EU	15 (3.58)	217 (51.79)
International (non-EU)	12 (1.40)	401 (46.63)
Current year of study		
Year 1	21 (1.07)	645 (32.86)
Year 2	42 (1.95)	713 (33.13)
Year 3	53 (2.72)	596 (30.53)
Year 4 (not penultimate year)	46 (4.86)	326 (34.42)
Penultimate year	75 (3.77)	616 (30.97)
Final year	66 (4.45)	396 (33.45)
Total	303 (100.00)	3,392 (100.00)

only

Key themes generated	Number of mentions	Percentage of students
Financial considerations	*4,284*	*80.92%*
Remuneration	4,080	*77.07%*
Fees incurred by medical practice, e.g. examinations, courses	155	*2.93%*
Pension	49	*0.93%*
Working in the NHS	*4,102*	*77.48%*
Work-life balance, rotas and flexibility	*1,749*	*33.04%*
Working conditions	*1,389*	*26.24%*
Levels of stress, responsibility and pressure	*337*	*6.37%*
Breaks, leave and non-clinical opportunities	*214*	*4.04%*
Resources, equipment, technology and facilities	*205*	*3.87%*
Incentives, benefits and perks, e.g. parking, accommodation, etc	*203*	*3.83%*
Visa status and citizenship	5	*0.09%*
Training and practice	*1,745*	*32.96%*
Autonomy of working location and reduction in rotational training	*525*	*9.92%*
Levels of competition for foundation posts, specialty training posts, and consultant posts	*446*	*8.42%*
Quality of training and teaching	*282*	*5.33%*
Streamlining foundation and specialty training	*174*	*3.29%*
"Service provision", non-clinical responsibilities, and bureaucracy	*126*	*2.38%*
Postgraduate training application and allocation processes	124	*2.34%*
Regulation of AHPs* and prioritisation of doctors' training and tasks	46	*0.87%*
Variety and degree of specialty exposure before training application	22	*0.42%*
The NHS and society	*1,672*	*31.58%*
Staffing levels	850	*16.06%*
Funding of the NHS, social care, and other health services	258	*4.87%*
Operational reform, efficiency and reduction in non-clinical middle management	174	*3.29%*
Bed availability, waiting lists, appointment duration and patient experience	125	*2.36%*
Prioritising mental well-being of NHS staff	105	*1.98%*
Changes in government leadership	64	*1.21%*
Addressing discrimination in the NHS	36	*0.68%*
Reversal of privatisation of the NHS and assurance of NHS' longevity	32	*0.60%*
Increased privatision of the NHS	28	*0.53%*
Culture and support	*994*	*18.78%*
Treatment and respect for doctors and AHPs	371	*7.01%*
Support for doctors and AHPs	306	*5.78%*
Workplace culture	224	*4.23%*
Staff morale	63	*1.19%*
Autonomy of practice, litigation, and the GMC	30	*0.57%*
Medical school and education	*194*	*3.66%*
Career, portfolio and specialty application guidance	78	*1.47%*
University degree funding and debt	71	*1.34%*
Quality, design and conditions of medical school education	24	*0.45%*
Medical school places and changes to entry requirements	15	*0.28%*
Degree length and flexibility	6	*0.11%*
Other	*110*	*2.08%*
Vague, uninterpretable, or otherwise uncategorisable	*110*	*2.08%*
Total	*5,294*	*100.00%*

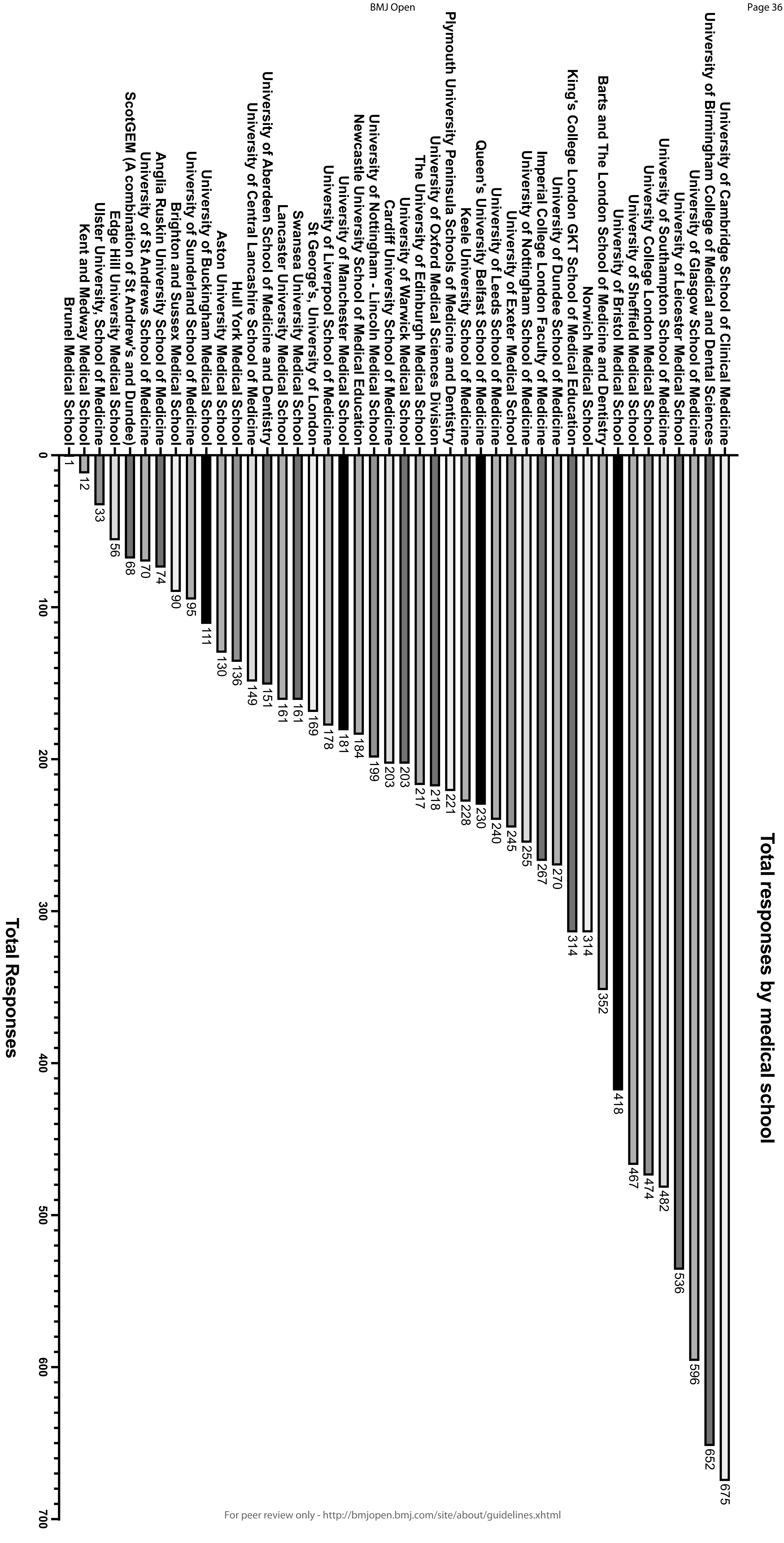
Total responses by medical school
Total Responses
0
100
200
300
400
500
600
700
University of Cambridge School of Clinical Medicine 675
University of Birmingham College of Medical and Dental Sciences 652
University of Glasgow School of Medicine 596
University of Leicester Medical School 536
University of Southampton School of Medicine 482
University College London Medical School 474
University of Sheffield Medical School 467
University of Bristol Medical School 418
Barts and The London School of Medicine and Dentistry 352
Norwich Medical School 314
King's College London GKT School of Medical Education 314
University of Dundee School of Medicine 270
Imperial College London Faculty of Medicine 267
University of Nottingham School of Medicine 255
University of Exeter Medical School 245
University of Leeds School of Medicine 240
Queen's University Belfast School of Medicine 230
Keele University School of Medicine 228
Plymouth University Peninsula Schools of Medicine and Dentistry 221
University of Oxford Medical Sciences Division 218
The University of Edinburgh Medical School 217
University of Warwick Medical School 203
Cardiff University School of Medicine 203
University of Nottingham - Lincoln Medical School 199
Newcastle University School of Medical Education 184
University of Manchester Medical School 181
University of Liverpool School of Medicine 178
St George's, University of London 169
Swansea University Medical School 161
Lancaster University Medical School 161
University of Aberdeen School of Medicine and Dentistry 151
University of Central Lancashire School of Medicine 149
Hull York Medical School 136
Aston University Medical School 130
University of Buckingham Medical School 111
University of Sunderland School of Medicine 95
Brighton and Sussex Medical School 90
Anglia Ruskin University School of Medicine 74
University of St Andrews School of Medicine 70
ScotGEM (A combination of St Andrew's and Dundee) 68
Edge Hill University Medical School 56
Ulster University, School of Medicine 33
Kent and Medway Medical School 12
Brunel Medical School 1

**Students' intention after graduation**	**Number (%)**	**Confidence Interval**
Complete both FY1 and FY2	8,806 (83.98)	[83.26, 84.67]
Complete FY1 and emigrate to practice medicine	1,101 (10.50)	[9.93,11.10]
Complete FY1 and leave medicine permanently	132 (1.26)	[1.06, 1.49]
Leave medicine permanently	104 (0.99)	[0.82, 1.20]
Emigrate to practice medicine	220 (2.10)	[1.84, 2.39]
Take a break or undertake further study	123 (1.17)	[0.98, 1.40]

For peer review only

Students' intention after the Foundation Programme	Number (%)	Confidence Interval
Enter specialty training in the UK	4,294 (48.76)	[47.72, 49.81]
Assume a non-training clinical job in the UK	1,859 (21.11)	[20.27, 21.98]
Emigrate to practice medicine abroad (including temporarily)	2,071 (23.52)	[22.64, 24.42]
Take a break or undertake further study	515 (5.85)	[5.38, 6.36]
Leave medicine permanently	67 (0.76)	[0.60, 0.97]

For peer review only

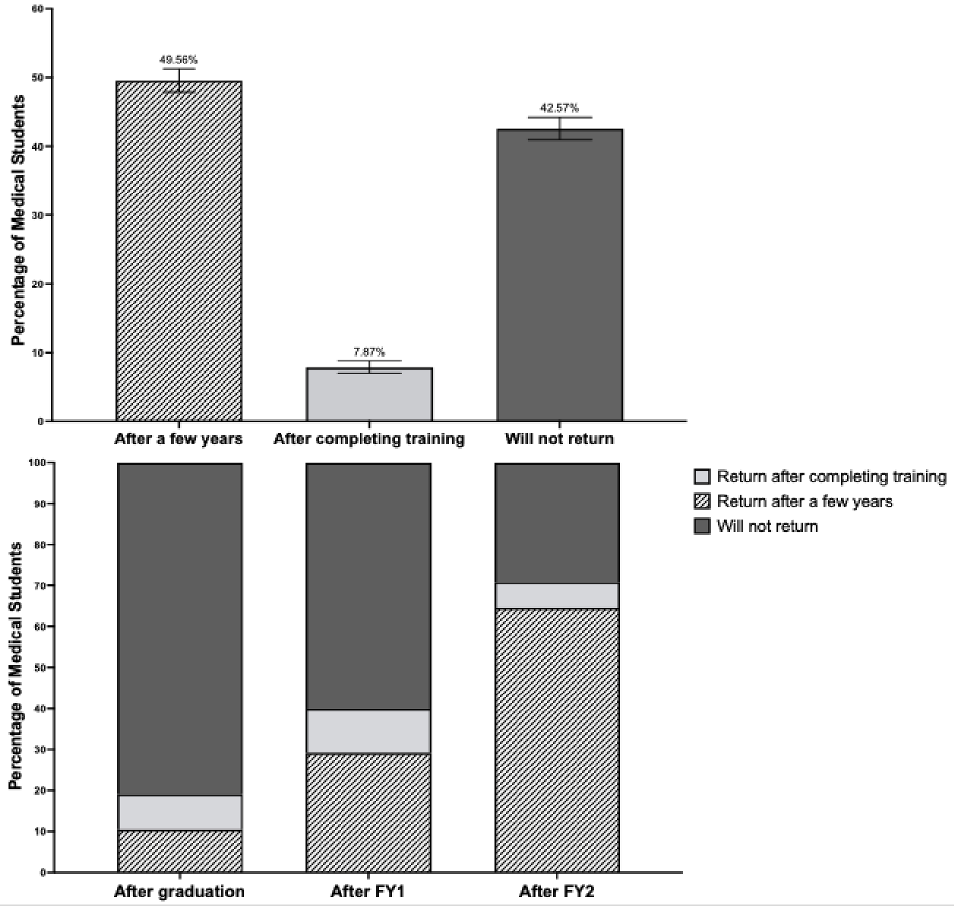
Percentage of Medical Students
49.56%
7.87%
42.57%
After a few years
After completing training
Will not return
Percentage of Medical Students
Return after completing training
Return after a few years
Will not return
After graduation
After FY1
After FY2

review only

Students' intention after graduation	Year 1	Year 2	Year 3	Year 4 (not penultimate year)	Penultimate Year	Final Year
Complete both FY1 and FY2	1616 (82.32)	1723 (80.07)	1643 (84.17)	759 (80.15)	1728 (86.88)	1337 (90.16)
Complete FY1 and emigrate to practice medicine	251 (12.79)	283 (13.15)	208 (10.66)	114 (12.04)	161 (8.09)	84 (5.66)
Complete FY1 and leave medicine permanently	8 (0.41)	15 (0.70)	24 (1.23)	18 (1.90)	37 (1.86)	30 (2.02)
Leave medicine permanently	10 (0.51)	23 (1.07)	17 (0.87)	20 (2.11)	23 (1.16)	11 (0.74)
Emigrate to practice medicine	61 (3.11)	65 (3.02)	45 (2.31)	19 (2.01)	20 (1.01)	10 (0.67)
Take a break or undertake further study	17 (0.87)	43 (2.00)	15 (0.77)	17 (1.80)	20 (1.01)	11 (0.74)

For peer review only

Students' intention after the Foundation Programme	Year 1	Year 2	Year 3	Year 4 (not penultimate year)	Penultimate Year	Final Year
Enter specialty training in the UK	1093 (67.64)	1071 (62.16)	866 (52.71)	298 (39.26)	621 (35.94)	345 (25.80)
Assume a non-training clinical job in the UK	109 (6.75)	197 (11.43)	326 (19.84)	192 (25.30)	554 (32.06)	481 (35.98)
Emigrate to practice medicine abroad (including temporarily)	333 (20.61)	365 (21.18)	343 (20.88)	193 (25.43)	435 (25.17)	402 (30.07)
Take a break or undertake further study	78 (4.83)	86 (4.99)	96 (5.84)	68 (8.96)	103 (5.96)	84 (6.28)
Leave medicine permanently	3 (0.19)	4 (0.23)	12 (0.73)	8 (1.05)	15 (0.87)	25 (1.87)

For peer review only

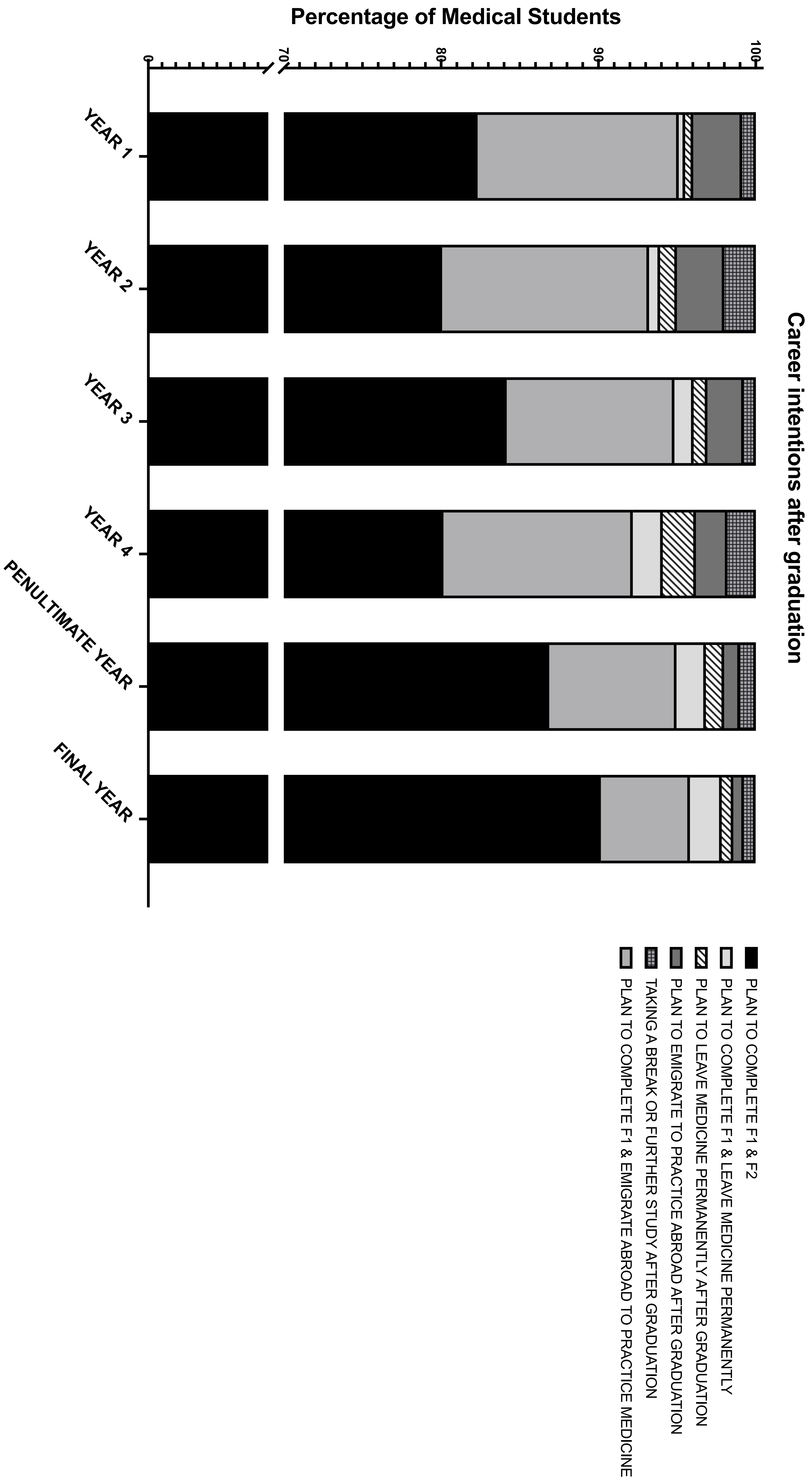
Percentage of Medical Students
0
70
80
90
100
Career intentions after graduation
YEAR 1
YEAR 2
YEAR 3
YEAR 4
PENULTIMATE YEAR
FINAL YEAR
PLAN TO COMPLETE F1 & F2
PLAN TO COMPLETE F1 & LEAVE MEDICINE PERMANENTLY
PLAN TO LEAVE MEDICINE PERMANENTLY AFTER GRADUATION
PLAN TO EMIGRATE TO PRACTICE ABROAD AFTER GRADUATION
TAKING A BREAK OR FURTHER STUDY AFTER GRADUATION
PLAN TO COMPLETE F1 & EMIGRATE ABROAD TO PRACTICE MEDICINE

Demographic subgroup	Return prospects		
	After a few years	After completing training	Will not return
Ethnicity			
White	1,133 (58.46)	131 (6.76)	674 (34.78)
Asian or Asian British	334 (36.66)	78 (8.56)	499 (54.77)
Black, Black British, Caribbean or African	79 (44.89)	15 (8.52)	82 (46.59)
Mixed or multiple ethnic groups	88 (46.07)	23 (12.04)	80 (41.88)
Other	40 (28.37)	18 (12.77)	83 (58.87)
Prefer not to say	7 (20.00)	2 (5.71)	26 (74.29)
Gender			
Female	1,165 (53.37)	175 (8.02)	843 (38.62)
Male	512 (42.99)	92 (7.72)	587 (49.29)
Non-binary	3 (25.00)	0 (0)	9 (75.00)
Prefer not to say	1 (16.67)	0 (0)	5 (83.33)
Level of education			
Postgraduate	311 (46.49)	51 (7.62)	307 (45.89)
Undergraduate	1,370 (50.31)	216 (7.93)	1137 (41.76)
Previous schooling			
Private education	578 (44.91)	113 (8.78)	596 (46.31)
State education	1,072 (52.96)	143 (7.07)	809 (39.97)
Prefer not to say	31 (38.27)	11 (13.58)	39 (48.15)
Fee status			
Home	1,572 (56.67)	221 (7.97)	981 (35.36)
EU	45 (20.74)	17 (7.83)	155 (71.43)
International (Non-EU)	64 (15.96)	29 (7.23)	308 (76.81)
Current year of study			
Year 1	297 (46.05)	65 (10.08)	283 (43.88)
Year 2	346 (48.53)	55 (7.71)	312 (43.76)
Year 3	281 (47.15)	55 (9.23)	260 (43.62)
Year 4 (not penultimate year)	163 (50.00)	25 (7.67)	138 (42.33)
Penultimate year	313 (50.81)	47 (7.63)	256 (41.56)
Final year	281 (56.65)	20 (4.03)	195 (39.31)
Total	1681 (49.56)	267 (7.87)	1444 (42.57)

For peer review only - http://bmjopen.bmj.com/site/about/guidelines.xhtml

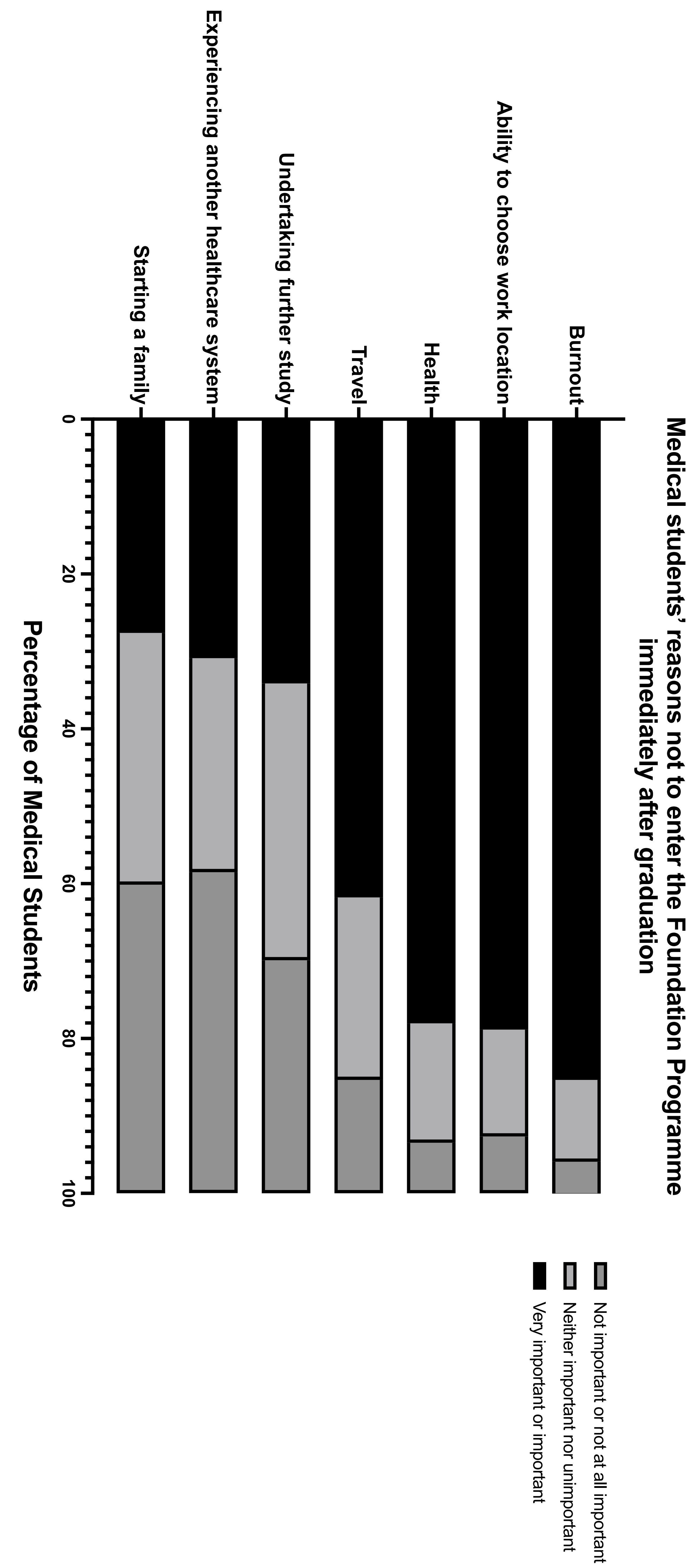
Medical students' reasons not to enter the Foundation Programme immediately after graduation
Burnout
Ability to choose work location
Health
Travel
Undertaking further study
Experiencing another healthcare system
Starting a family
0
20
40
60
80
100
Percentage of Medical Students
Not important or not at all important
Neither important nor unimportant
Very important or important

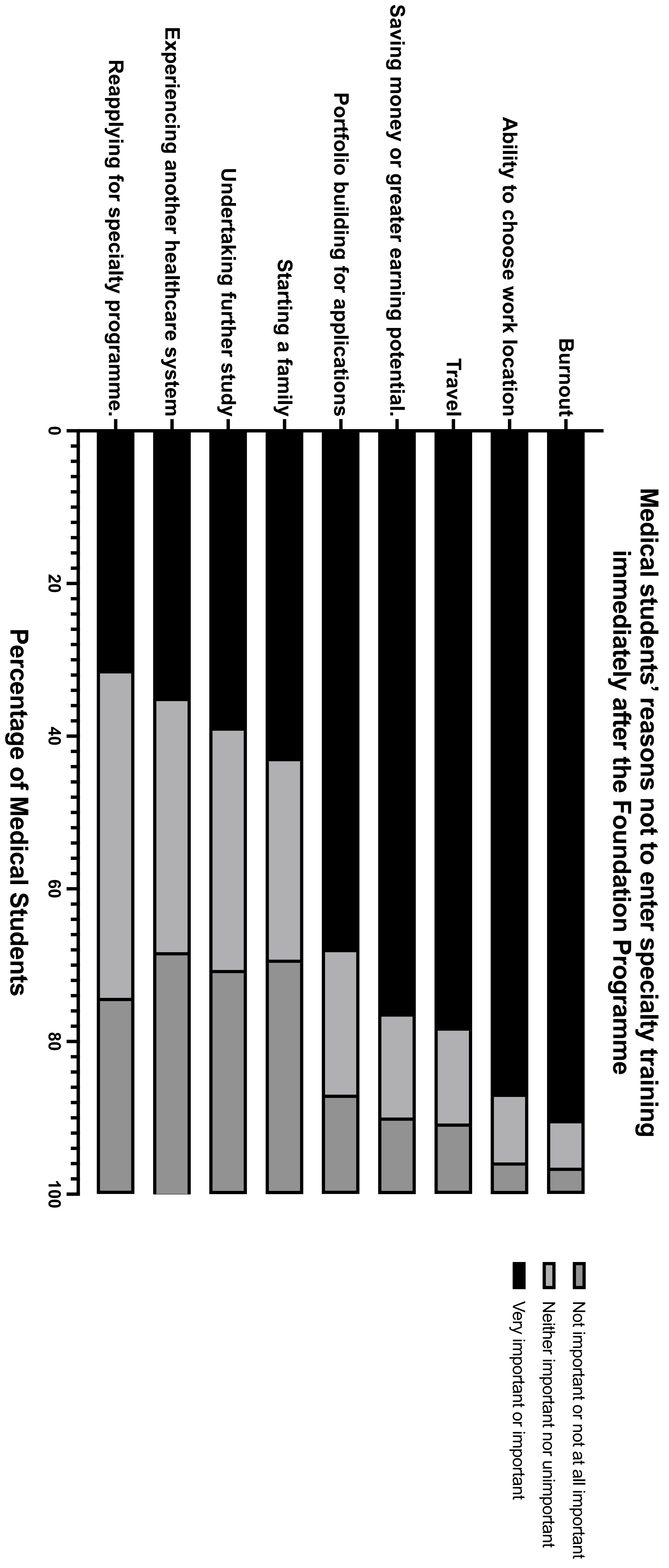
Medical students' reasons not to enter specialty training immediately after the Foundation Programme
Reapplying for specialty programme.
Experiencing another healthcare system
Undertaking further study
Starting a family
Portfolio building for applications
Saving money or greater earning potential.
Travel
Ability to choose work location
Burnout
0
20
40
60
80
100
Percentage of Medical Students
Not important or not at all important
Neither important nor unimportant
Very important or important

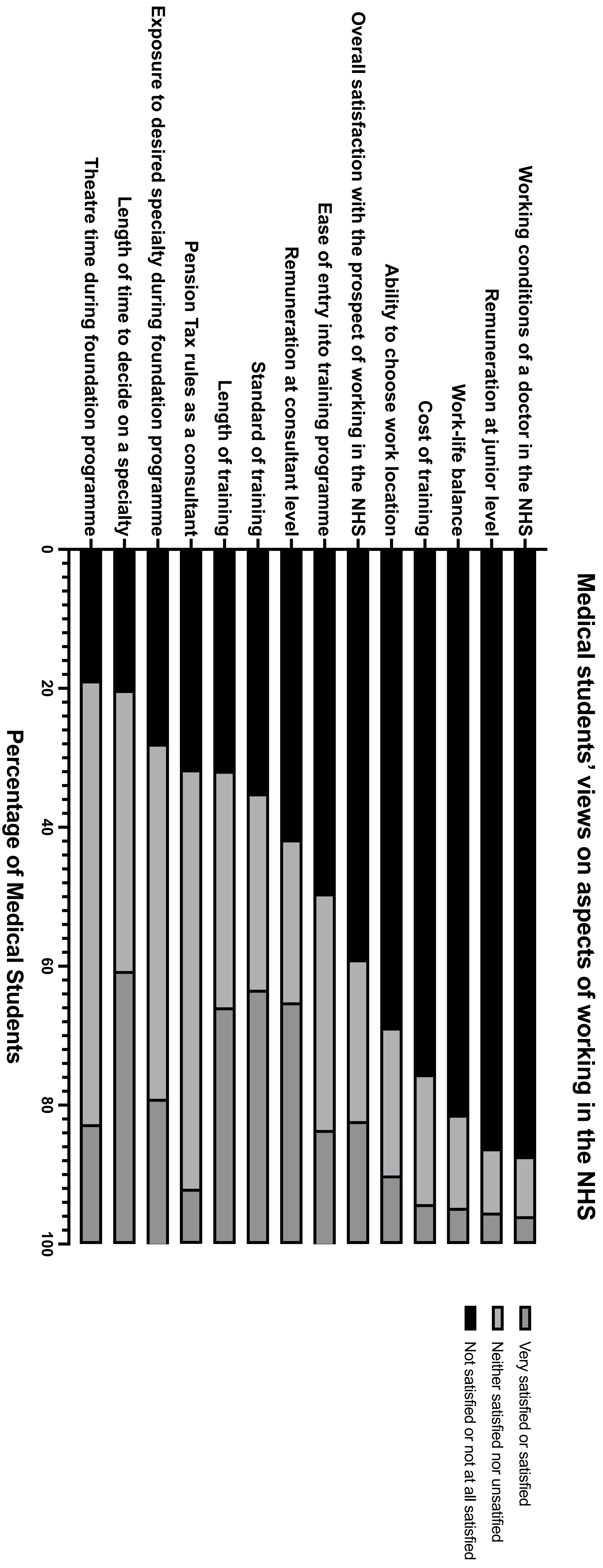
Medical students' views on aspects of working in the NHS
Working conditions of a doctor in the NHS
Remuneration at junior level
Work-life balance
Cost of training
Ability to choose work location
Overall satisfaction with the prospect of working in the NHS
Ease of entry into training programme
Remuneration at consultant level
Standard of training
Length of training
Pension Tax rules as a consultant
Exposure to desired specialty during foundation programme
Length of time to decide on a specialty
Theatre time during foundation programme
0
20
40
60
80
100
Percentage of Medical Students
Very satisfied or satisfied
Neither satisfied nor unsatified
Not satisfied or not at all satisfied

Demographics

AIMS - Ascertaining the career Intentions of UK Medical Students' post-graduation: a cross-sectional survey

Thank you for taking part in the study. Please note that participating in this survey is entirely optional.

In 2010, 83.1% of Foundation Year 2 (F2) doctors went into further training. In 2019, this number was only 34.9%. This represents a significant change in the makeup of doctors in the UK on a backdrop of a wider NHS staffing challenge. AIMS endeavours to understand the factors involved in medical students' decision-making around their future career. Specifically, we are interested in what students' current career plans are, and why they may, or may not choose to pursue specialty training, or a medical career more broadly, in the UK. We are also hoping to understand current views on the prospect of working in the NHS.

All responses will remain confidential. Your email address will only be visible to the study leads and will be deleted from our records once all data has been collected (unless you consent to being followed up at the end of the survey) and there is no need for further communication. You may withdraw from the study at any point by contacting Tomas Ferreira, tf385@cam.ac.uk.

By submitting your answers to the survey, you consent to us collecting this data and acknowledging that anonymised data may be published and used for purposes beyond this study. Ethical approval was granted by the University of Cambridge Research Ethics Committee (PRE.2022.124) on 5 January 2023.

All participants will be entered into a prize draw for the chance to win £300!

I understand that my participation is voluntary and that I am free to withdraw at any time without giving a reason and I consent to participate in this study.

○ Yes

Email Address

Please enter your institutional email address (ending in 'ac.uk'. We will use this to verify your student status and we may contact you to notify you of a prize win or for clarification of responses). Please ensure there are no spaces at the end of your email.

Age

Gender

Which of the below options best describes your ethnicity?

University

Year of study - Please read description.

(as of September 2022)

- If you are in your fourth year of study and it is your final year, please select final year (i.e., GEM)

- If you are in your fourth year of study but it is your penultimate year, please select penultimate year.

- If you are currently intercalating, please select your current year of study (e.g., intercalating between 3rd and 4th year on a 5 year course please select Year 4).

- Treat the first year of a GEM course as still equivalent to first year.

- If you are in a "Foundation" or "Gateway" year (also known as Y0), please select Year 1.

- ○ Year 1
- ○ Year 2
- ○ Year 3
- ○ Year 4 (not penultimate year)
- ○ Penultimate year
- ○ Final year

What is your expected graduation year?

Do you have a previous or intercalated degree?

- ○ Yes, prior to studying Medicine.
- ○ Yes, an intercalated degree.
- ○ Yes, both.
- ○ Not yet, but intend on intercalating.

- ◯ Not yet, but currently intercalating.
- ◯ No.

What is your student fee status?

- ◯ Home
- ◯ EU
- ◯ International (Non-EU)

Did you, at any point in your education, attend a fee-paying independent school?

E.g., private school.

- ◯ Yes
- ◯ No
- ◯ Prefer not to say

Intentions

Do you intend to join the NHS Foundation Programme after graduation?

- ◯ Yes - plan to complete F1 & F2
- ◯ Yes - plan to complete F1 & emigrate to practice abroad
- ◯ Yes - plan to complete F1 & leave medicine permanently.
- ◯ No - plan on leaving medicine permanently.
- ◯ No - plan on emigrating
- ◯ No - plan on taking a break or undertaking further study.

What do you intend to do after completing the NHS Foundation Programme?

- ◯ Enter specialty training in the UK
- ◯ Non-training clinical job in the UK, e.g. 'F3 year', JCF or CTF
- ◯ Emigrating to practice medicine abroad (including temporarily)
- ◯ Taking a break or undertaking further study

○ Leaving medicine permanently

You have indicated your intention to leave medicine permanently. In which industry do you plan to work after leaving medicine? If unsure, please enter N/A"

In which country do you intend to practice?

If you are unsure, please enter N/A.

Reasons for emigrating to practice abroad

In your previous answers, you have indicated your intentions to practice medicine abroad.

Please indicate the level of importance of the below factors in your decision making

	Very important	Important	Neither important nor unimportant	Not important	Not at all important
Remuneration or pay at junior level	○	○	○	○	○
Remuneration or pay at consultant level	○	○	○	○	○
Work-life balance	○	○	○	○	○
Family	○	○	○	○	○
Desire for a life change	○	○	○	○	○
Ease of entry into training (competition)	○	○	○	○	○
Length of training	○	○	○	○	○
Standard of training	○	○	○	○	○
Ability to choose work location	○	○	○	○	○

Working conditions of a doctor in the NHS	◯	◯	◯	◯	◯
Uncertainty about which specialty to pursue	◯	◯	◯	◯	◯

You have indicated that you intend to emigrate to practice medicine, do you intend on returning to the UK?

- ◯ Yes - after a few years
- ◯ Yes - after I complete my training
- ◯ No

Reasons for leaving medicine permanently

In your previous answers, you have indicated your intentions to leave medicine permanently.

Please indicate the level of importance of the below factors in your decision making.

	Very important	Important	Neither important nor unimportant	Not important	Not at all important
Remuneration or pay at junior level	◯	◯	◯	◯	◯
Remuneration or pay at consultant level	◯	◯	◯	◯	◯
Work-life balance	◯	◯	◯	◯	◯
Family	◯	◯	◯	◯	◯
Desire for a life change	◯	◯	◯	◯	◯
Ability to choose work location	◯	◯	◯	◯	◯
Working conditions of a doctor in the NHS	◯	◯	◯	◯	◯
Stress levels associated with profession	◯	◯	◯	◯	◯
Burnout	◯	◯	◯	◯	◯

Reasons for not entering specialty training immediately after F2

In your previous answers, you have indicated your intentions to not enter specialty training immediately after completing your F2 year. Please indicate the level of importance of the below factors in your decision making.

	Very important	Important	Neither important nor unimportant	Not important	Not at all important
Saving money or greater earning potential.	○	○	○	○	○
Undertaking further study	○	○	○	○	○
Portfolio building for applications	○	○	○	○	○
Travel	○	○	○	○	○
Experiencing another healthcare system	○	○	○	○	○
Starting a family	○	○	○	○	○
Reapplying for specialty programme.	○	○	○	○	○
Ability to choose work location	○	○	○	○	○
Burnout	○	○	○	○	○
Uncertainty about which specialty to pursue	○	○	○	○	○

Reasons for not entering foundation training immediately after graduation

In your previous answers, you have indicated your intentions to not enter foundation training immediately after graduation. Please indicate the level of importance of the below factors in your decision making.

	Very important	Important	Neither important nor unimportant	Not important	Not at all important

Undertaking further study	○	○	○	○	○
Travel	○	○	○	○	○
Experiencing another healthcare system	○	○	○	○	○
Starting a family	○	○	○	○	○
Ability to choose work location	○	○	○	○	○
Health	○	○	○	○	○
Burnout	○	○	○	○	○

Views on a career in the NHS

For each of the points below, how would you describe your level of satisfaction regarding their current status in the NHS?

	Very satisfied	Satisfied	Neither satisfied nor unsatisfied	Not satisfied	Not at all satisfied
Remuneration or pay at junior level	○	○	○	○	○
Remuneration or pay at consultant level	○	○	○	○	○
Work-life balance	○	○	○	○	○
Ability to choose work location	○	○	○	○	○
Ease of entry into training (competition)	○	○	○	○	○
Length of training	○	○	○	○	○
Standard of training	○	○	○	○	○
Working conditions of a doctor in the NHS	○	○	○	○	○
Exposure to desired specialty during foundation programme	○	○	○	○	○
Theatre time during foundation programme	○	○	○	○	○

Cost of training (i.e., mandatory exams, courses, memberships)	○	○	○	○	○
Length of time to decide on a specialty	○	○	○	○	○
Pension Tax rules as a consultant	○	○	○	○	○
Overall satisfaction with the prospect of working in the NHS	○	○	○	○	○

Are you certain about which specialty you wish to pursue?

- ○ Very certain
- ○ Somewhat certain
- ○ Neither certain nor uncertain
- ○ Somewhat uncertain
- ○ Very uncertain

Which specialty (or specialties) most interest you?

Select **up to** a maximum of 3 options (if you are certain, please select only one)

- ☐ Acute internal medicine
- ☐ Allergy
- ☐ Anaesthetics
- ☐ Audio vestibular medicine
- ☐ Cardio-thoracic surgery
- ☐ Cardiology
- ☐ Clinical genetics
- ☐ Clinical neurophysiology
- ☐ Clinical oncology
- ☐ Community sexual and reproductive health
- ☐ Dermatology
- ☐ Emergency medicine
- ☐ Endocrinology and diabetes mellitus
- ☐ Gastro-enterology
- ☐ General practice
- ☐ General surgery

- ☐ Genito-urinary medicine
- ☐ Geriatric medicine
- ☐ Haematology
- ☐ Histopathology
- ☐ Immunology
- ☐ Infectious diseases
- ☐ Intensive care medicine
- ☐ Medical microbiology
- ☐ Medical oncology
- ☐ Neurology
- ☐ Neurosurgery
- ☐ Nuclear medicine
- ☐ Obstetrics and gynaecology
- ☐ Occupational medicine
- ☐ Ophthalmology
- ☐ Oral and maxillo-facial surgery
- ☐ Otolaryngology (ENT)
- ☐ Paediatric surgery
- ☐ Paediatrics
- ☐ Palliative medicine
- ☐ Pathology
- ☐ Plastic surgery
- ☐ Psychiatry
- ☐ Public health medicine
- ☐ Radiology
- ☐ Rehabilitation medicine
- ☐ Renal medicine
- ☐ Respiratory medicine
- ☐ Rheumatology
- ☐ Sport and exercise medicine
- ☐ Trauma and orthopaedic surgery
- ☐ Tropical medicine
- ☐ Urology
- ☐ Vascular surgery

What steps could be taken to improve the prospect of working in the NHS?

(Optional)

Do you consent to being contacted by us for potential follow-up studies regarding your career intentions?

We will store your email address to contact you in the future.

- ○ Yes
- ○ No

Powered by Qualtrics

For peer review only

Eligible Medical Schools and Approved Programmes

A combination of the universities of Dundee and St. Andrews (ScotGEM)
A combination of the University of Brighton and the University of Sussex
A combination of the University of Hull and the University of York
Anglia Ruskin School of Medicine
Aston Medical School
Brunel University London Medical School
Cardiff University
Edge Hill University Medical School
Imperial College London
Keele University
Kent and Medway Medical School
King's College London
Lancaster University
Queen Mary University of London
St George's University of London
Swansea University
The Queen's University of Belfast
The University of Aberdeen
The University of Birmingham
The University of Bristol
The University of Buckingham
The University of Cambridge
The University of Central Lancashire
The University of Dundee
The University of Dundee
The University of East Anglia
The University of Edinburgh
The University of Exeter
The University of Glasgow
The University of Leeds
The University of Leicester
The University of Liverpool
The University of Manchester
The University of Newcastle
The University of Nottingham
The University of Oxford
The University of Plymouth
The University of Sheffield
The University of Southampton
The University of St Andrew's
The University of Warwick
Ulster University School of Medicine
University College London
University of Sunderland School of Medicine

Excluded for lack of cohort at time of recruitment:

- University of Chester Medical School
- Three Counties Medical School

For peer review only

AIMS UNIVERSITY OF CAMBRIDGE School of Clinical Medicine

Participant Information Sheet

Ascertaining the career Intentions of Medical Students in the UK post-graduation: a cross-sectional study

Thank you for your interest in participating in this study. Please take a moment to read the following information. If you have any questions or concerns, please contact the principal researcher, Tomas Ferreira at tf385@cam.ac.uk.

What is the aim of this study? This study aims to determine current medical students' career intentions post-graduation and post-foundation training, to identify factors involved in decision making for students' career choices and to analyse medical students' views on how the prospect of working in the NHS could be improved.

Why have I been selected to take part? All medical students currently studying at UK medical schools recognised by the General Medical Council (GMC) are being invited to take part in the questionnaire.

What do I have to do? If you decide to participate in this study, you will be asked to complete a questionnaire about your background, your career intentions after graduation and after foundation training, and your motivations for these answers. This study is voluntary. If you choose to participate, you will be asked to complete the survey by clicking on the link found at the end of this document. This survey is expected to take about 4-7 minutes to complete, but there is no time limit. No background knowledge is required. By submitting the survey, you consent to the collection and storage of data in accordance with the UK General Data Protection Regulation (GDPR) within the survey. For more information on GDPR please click on the following link: https://gdpr-info.eu.

Do I have to participate? Participation is entirely voluntary. You may withdraw at any point during the questionnaire for any reason, before submitting your answers, by closing the browser. In cases of withdrawal from the study prior to submission of the survey, no data is recorded. If you have already submitted data and wish to withdraw from the study, please contact tf385@cam.ac.uk by 31st March 2023.

Who has approved this study? This study has been reviewed and approved by the University of Cambridge's Research Ethics Committee on the 5th of January 2023, reference PRE.2022.124.

How will my data be used? All answers will be anonymous, and we will take all reasonable precautions to ensure that they remain confidential. Data will be stored in a password-protected file and may be used in academic publications. Your IP address will not be stored. After completion of data collection, no email addresses will be stored unless you consent to being followed up via the survey's final question. Prior to completion of data collection, we will store your institutional email address to confirm your student status. Research data will be stored for a minimum of ten years after publication or public release.

Who will have access to my data? Qualtrics is the data controller of the personal data held about you and, as such, will determine how your personal data are used. Their privacy notice can be found here: https://www.qualtrics.com/privacy-statement. Qualtrics will share any email address you provide and your anonymised responses with the University of Cambridge, for the purposes of research as highlighted above. Researchers involved in the project will have access to this anonymised data.

Are there any benefits to taking part? Although there are no immediate individual benefits to participating in this survey, you are given the opportunity to contribute to research which may impact you. You may find this survey an opportunity to self-reflect on your career plans after you graduate. Additionally, all participants will be entered into a prize draw for a chance to win £300!

Will the research be published? The findings of this study may be published in peer-reviewed journals, presented at conferences and a summary of the findings will be made available on social media.

For peer review only

AIMS

UNIVERSITY OF CAMBRIDGE
School of Clinical Medicine

Are there any possible risks involved with my participation? There are no anticipated disadvantages, side effects, risks, and/or discomforts of taking part in this study. If participating in the study leads to distress, you may stop the survey at any time. If your distress continues after leaving the survey, we have provided a list of supportive services nationwide that can be helpful and that you might consider contacting (appears at the close of survey).

Who do I contact if I have a concern about the study or I wish to complain? If you have a concern about any aspect of this project, please speak to the principal researcher tf385@cam.ac.uk. If you remain unhappy or wish to make a formal complaint, please contact the Research and Information Governance, School of Clinical Medicine, University of Cambridge: Research.Governance@medschl.cam.ac.uk.

How do I find out what was learned in this study? This study is expected to be completed by April 2023. If you would like a brief summary of the results, please write to us by email to request information

Who to contact for further details? For any further questions or more information on the study, please contact us on the following email address: tf385@cam.ac.uk.

Link to the survey: https://cambridge.eu.qualtrics.com/jfe/form/SV_cx55RTspDLTIzWK

Kind Regards,

Tomas Ferreira
AIMS Study Lead

Dr. Rita Horvath
Supervisor, Director of Research, Horvath Laboratory, Department of Clinical Neurosciences, University of Cambridge

For peer review only

STROBE Statement—Checklist of items that should be included in reports of ***cross-sectional studies***

	Item No	Recommendation
**Title and abstract**	1	(*a*) Indicate the study's design with a commonly used term in the title or the abstract
		(*b*) Provide in the abstract an informative and balanced summary of what was done and what was found
**Introduction**		
Background/rationale	2	Explain the scientific background and rationale for the investigation being reported
Objectives	3	State specific objectives, including any prespecified hypotheses
**Methods**		
Study design	4	Present key elements of study design early in the paper
Setting	5	Describe the setting, locations, and relevant dates, including periods of recruitment, exposure, follow-up, and data collection
Participants	6	(*a*) Give the eligibility criteria, and the sources and methods of selection of participants
Variables	7	Clearly define all outcomes, exposures, predictors, potential confounders, and effect modifiers. Give diagnostic criteria, if applicable
Data sources/ measurement	8*	For each variable of interest, give sources of data and details of methods of assessment (measurement). Describe comparability of assessment methods if there is more than one group
Bias	9	Describe any efforts to address potential sources of bias
Study size	10	Explain how the study size was arrived at
Quantitative variables	11	Explain how quantitative variables were handled in the analyses. If applicable, describe which groupings were chosen and why
Statistical methods	12	(*a*) Describe all statistical methods, including those used to control for confounding
		(*b*) Describe any methods used to examine subgroups and interactions
		(*c*) Explain how missing data were addressed
		(*d*) If applicable, describe analytical methods taking account of sampling strategy
		(*e*) Describe any sensitivity analyses
**Results**		
Participants	13*	(a) Report numbers of individuals at each stage of study—eg numbers potentially eligible, examined for eligibility, confirmed eligible, included in the study, completing follow-up, and analysed
		(b) Give reasons for non-participation at each stage
		(c) Consider use of a flow diagram
Descriptive data	14*	(a) Give characteristics of study participants (eg demographic, clinical, social) and information on exposures and potential confounders
		(b) Indicate number of participants with missing data for each variable of interest
Outcome data	15*	Report numbers of outcome events or summary measures
Main results	16	(*a*) Give unadjusted estimates and, if applicable, confounder-adjusted estimates and their precision (eg, 95% confidence interval). Make clear which confounders were adjusted for and why they were included
		(*b*) Report category boundaries when continuous variables were categorized
		(*c*) If relevant, consider translating estimates of relative risk into absolute risk for a meaningful time period
Other analyses	17	Report other analyses done—eg analyses of subgroups and interactions, and sensitivity analyses

For peer review only

For peer review only - http://bmjopen.bmj.com/site/about/guidelines.xhtml

**Discussion**		
Key results	18	Summarise key results with reference to study objectives
Limitations	19	Discuss limitations of the study, taking into account sources of potential bias or imprecision. Discuss both direction and magnitude of any potential bias
Interpretation	20	Give a cautious overall interpretation of results considering objectives, limitations, multiplicity of analyses, results from similar studies, and other relevant evidence
Generalisability	21	Discuss the generalisability (external validity) of the study results
**Other information**		
Funding	22	Give the source of funding and the role of the funders for the present study and, if applicable, for the original study on which the present article is based

*Give information separately for exposed and unexposed groups.

Note: An Explanation and Elaboration article discusses each checklist item and gives methodological background and published examples of transparent reporting. The STROBE checklist is best used in conjunction with this article (freely available on the Web sites of PLoS Medicine at http://www.plosmedicine.org/, Annals of Internal Medicine at http://www.annals.org/, and Epidemiology at http://www.epidem.com/). Information on the STROBE Initiative is available at www.strobe-statement.org.

For peer review only

BMJ Open

Career intentions of medical students in the United Kingdom: a national, cross-sectional study (AIMS Study)

Journal:	*BMJ Open*
Manuscript ID	bmjopen-2023-075598.R1
Article Type:	Original research
Date Submitted by the Author:	13-Jul-2023
Complete List of Authors:	Ferreira, Tomas; University of Cambridge School of Clinical Medicine<br>Collins, Alexander; Imperial College London, School of Public Health, Faculty of Medicine<br>Feng, Oliver; University of Cambridge, Statistical Laboratory, Centre for Mathematical Sciences<br>Samworth, Richard; University of Cambridge, Statistical Laboratory<br>Horvath, Rita; University of Cambridge School of Clinical Medicine<br>., the AIMS Collaborative; University of Cambridge School of Clinical Medicine
**Primary Subject Heading**:	Medical education and training
Secondary Subject Heading:	Medical management, Health economics, Health informatics, Health policy, Health services research
Keywords:	Health policy < HEALTH SERVICES ADMINISTRATION & MANAGEMENT, QUALITATIVE RESEARCH, Health Education, Organisation of health services < HEALTH SERVICES ADMINISTRATION & MANAGEMENT, MEDICAL EDUCATION & TRAINING, Health economics < HEALTH SERVICES ADMINISTRATION & MANAGEMENT

SCHOLARONE™
Manuscripts

I, the Submitting Author has the right to grant and does grant on behalf of all authors of the Work (as defined in the below author licence), an exclusive licence and/or a non-exclusive licence for contributions from authors who are: i) UK Crown employees; ii) where BMJ has agreed a CC-BY licence shall apply, and/or iii) in accordance with the terms applicable for US Federal Government officers or employees acting as part of their official duties; on a worldwide, perpetual, irrevocable, royalty-free basis to BMJ Publishing Group Ltd ("BMJ") its licensees and where the relevant Journal is co-owned by BMJ to the co-owners of the Journal, to publish the Work in this journal and any other BMJ products and to exploit all rights, as set out in our licence.

The Submitting Author accepts and understands that any supply made under these terms is made by BMJ to the Submitting Author unless you are acting as an employee on behalf of your employer or a postgraduate student of an affiliated institution which is paying any applicable article publishing charge ("APC") for Open Access articles. Where the Submitting Author wishes to make the Work available on an Open Access basis (and intends to pay the relevant APC), the terms of reuse of such Open Access shall be governed by a Creative Commons licence – details of these licences and which Creative Commons licence will apply to this Work are set out in our licence referred to above.

Other than as permitted in any relevant BMJ Author's Self Archiving Policies, I confirm this Work has not been accepted for publication elsewhere, is not being considered for publication elsewhere and does not duplicate material already published. I confirm all authors consent to publication of this Work and authorise the granting of this licence.

For peer review only

Career intentions of medical students in the United Kingdom: a national, cross-sectional study (AIMS Study)

Tomas Ferreira[1], Alexander M. Collins[2], Oliver Feng[3], Richard J. Samworth[3], Rita Horvath[1], and the AIMS collaborative

Affiliations

[1] School of Clinical Medicine, University of Cambridge, Cambridge, United Kingdom

[2] School of Public Health, Faculty of Medicine, Imperial College London, London, United Kingdom

[3] Statistical Laboratory, Centre for Mathematical Sciences, University of Cambridge, Cambridge, United Kingdom

Postal address of corresponding author:

Tomas Ferreira

Cambridge Centre for Brain Repair, Ed Adrian Building,

Cambridge,

CB2 0PY

tf385@cam.ac.uk

For peer review only

Abstract

Objective: To determine current United Kingdom (UK) medical students' career intentions after graduation and upon completing the Foundation Programme (FP), and to ascertain the motivations behind these intentions.

Design: Cross-sectional, mixed-methods survey of UK medical students, using a non-random sampling method.

Setting: All 44 UK medical schools recognised by the General Medical Council.

Participants: 10,486 medical students.

Primary and secondary outcomes: Career intentions of medical students post-graduation and post-Foundation Programme, motivations behind these career intentions, characterising the medical student population and correlating demographic factors and propensity to leave the National Health Service (NHS).

Results: The majority of participating students (8,806/10,486, 83.98%) planned to complete both years of the FP after graduation, with under half of these students (4,294/8,806, 48.76%) intending to pursue specialty training thereafter. A subanalysis of career intentions after the FP by year of study revealed a significant decrease in students' intentions to enter specialty training as they advanced through medical school. Approximately a third of surveyed students (3,392/10,486, 32.35%) intended to emigrate to practise medicine, with 42.57% (n=1,444) of those students not planning to return. In total, 2.89% of students intended to leave medicine altogether (n=303). Remuneration, work-life balance, and working conditions were important factors in decision-making regarding emigration and leaving the profession. Subgroup analyses based on gender, type of schooling, fee type, and educational background were performed. Qualitative thematic analysis revealed that the most commonly cited factors that would improve the prospect of working in the NHS

For peer review only

included improvements to remuneration, flexibility and work-life balance, working conditions, staffing levels, and autonomy in the location of work.

Conclusions: The AIMS study highlights UK students' views and career intentions, revealing a concerning proportion considering alternative careers or emigration. Addressing factors such as remuneration, work-life balance, and working conditions may increase retention of doctors and improve workforce planning efforts.

Word count: 5,789

Strengths and Limitations:

- This represents the largest ever study of UK medical students, and the largest study investigating medical students' career intentions, providing valuable insights into their future plans.
- This comprehensive survey addresses a topical and critical issue, providing important findings with significant implications for the NHS.
- Due to the cross-sectional design of the study, it captures a 'snapshot' in time, and is thus unable to reflect changes in students' career intentions over time.
- A high consent rate of 71.29% for follow-up studies allows for the possibility of longitudinal validation and observation of changes over time.
- Despite being the largest study of UK medical students, approximately 21.50% of all UK medical students participated, which may introduce selection bias as it may be that this survey appealed to those already intending to leave the NHS or are interested in this topic.

For peer review only

Introduction

Training doctors is a costly investment, and measuring the extent of attrition from the health service in the country of training is crucial to ensure optimal value. Understanding medical students' career plans and trajectories post-graduation is an important factor in effective workforce planning and retention.

There are several factors behind doctors' motivations to emigrate to practise medicine abroad or leave the profession entirely. Commonly cited themes among doctors in the United Kingdom (UK) include pay erosion and low pay compared to alternative destinations, working conditions within the National Health Service (NHS), wellbeing, work-life balance, and better training opportunities abroad (1, 2).

For peer review only - http://bmjopen.bmj.com/site/about/guidelines.xhtml

The UK has 3.2 doctors for every 1,000 people, ranking 25th amongst the Organisation for Economic Co-operation and Development (OECD) countries. This represents the lowest number of doctors per capita among European countries in the OECD (3). The British government has responded to the issue of an insufficient number of doctors by opening new medical schools and expanding the student capacity of existing ones (4, 5). Recently, there have been proposals to double the number of medical school places as a solution to address the shortage of doctors in the NHS (6). However, without addressing the issue of doctors leaving the NHS, increasing the number of medical students is unlikely to provide a sustainable long-term solution. Recruitment efforts may be ineffective if the retention of doctors is not simultaneously addressed. This highlights the pressing need for a multifaceted approach that considers both recruitment and retention strategies to effectively address the workforce challenges in the NHS.

Medical Education in the United Kingdom

In the UK, after medical school, medical graduates enter the Foundation Programme, a two-year programme consisting of a series of 4-month or 6-month rotations through various specialties and clinical settings. The successful completion of the programme's first year (FY1) provides doctors with full registration with the UK's medical regulator, the General Medical Council (GMC). This registration is recognised internationally. In many cases, individuals who leave the NHS after FY1 rather than immediately following graduation may do so because of the opportunities available with the full registration provided by completion of FY1. Completion of the second year of the programme (FY2) allows applicants to apply for specialist training pathways, such as those in psychiatry, neurosurgery, and general practice (7, 8).

To the best of our knowledge, this is the largest study of UK medical students to date. This mixed-methods study aimed to investigate current medical students' career intentions after graduation and upon completing the Foundation Programme, and the motivations behind these intentions. Secondary outcomes included determining which demographic factors alter the propensity to pursue different career paths available to a medical graduate, determining which specialties medical students plan to pursue and understanding current views on the prospect of working in the NHS. These data provide important answers to the current workforce challenges within the NHS and could help address some of the concerns of those making up the future of the profession.

Methods

Study Design

For peer review only

AIMS (Ascertaining the career Intentions of UK Medical Students) was a national, multi-centre, cross-sectional study of medical students conducted in accordance with its protocol (9). The study employed a non-random sampling method to recruit participants from 44 UK medical schools recognised by the General Medical Council (GMC).

A novel, self-administered, 71-item questionnaire was developed. The survey was hosted on the Qualtrics survey platform (Provo, Utah, USA), a GDPR-compliant online platform that supports both mobile and desktop devices. Prior to completing the survey, all participants provided informed consent. All participants were asked to complete the first section of the survey (Questions 1 to 11). Subsequent question visibility was dependent on participants' answers to previous questions. The fewest number of items available to any one participant was 30, and the largest was 43. Questions were structured using a combination of Likert scale matrices, multiple-choice options, and free-text entry to broaden the capture of sentiment nuance and improve precision in the data. A copy of the questionnaire and the Participant Information Sheet can be found in the Supplemental Materials.

Participant recruitment and eligibility

To minimise bias, a network of approximately 200 collaborators across 42 medical schools was recruited prior to the study launch to ensure equitable access to the survey. All medical students in all year groups were eligible to apply, and positions were advertised via medical student societies, social media, and internal medical school newsletters. They were responsible for maximising the response numbers within their year group at their medical schools. Collaborators were instructed to use a range of distribution methods, including social media, internal bulletins/newsletters, and email communication. This approach aimed to achieve a representative sample and improve the generalisability of our findings.

In order to qualify for collaborative authorship, students were required to achieve a minimum of 35 responses, or 15% of their year group (whichever number was lowest). The survey was disseminated between January 16, 2023, and March 27, 2023.

To be eligible for participation, individuals must have been actively enrolled in a UK medical school acknowledged by the General Medical Council (GMC) and listed by the Medical School Council (MSC) (Supplemental Materials). Certain new medical schools have received approval from the GMC but have yet to admit their inaugural cohort of students. These schools were therefore excluded from our study since they had no medical students at the time of data collection.

Data Collection

For peer review only - http://bmjopen.bmj.com/site/about/guidelines.xhtml

For peer review only

The survey consisted of five parts. Part 1 involved a background and demographics section, which all participants were required to answer. In Part 2, participants were asked to indicate their intended career paths immediately after graduation and after foundation training (if applicable). Part 3 explored the factors influencing their decision-making. Part 4 surveyed their current specialty preferences. The final part featured a free-entry text box inviting participants to articulate how the prospect of working in the NHS could be improved. Consent for follow-up studies was also obtained in this section.

Data processing and storage

Each response was restricted to a single institutional email address to mitigate the risk of data duplication. Any replicated email entries were removed prior to data analysis. In cases where identical entries contained distinct responses, the most recent entry was retained. Entries where respondents did not provide a valid institutional email address were removed prior to data analysis to preserve the integrity of the study.

Quantitative data analysis

Descriptive analysis was carried out with Microsoft Excel (v16.71) (Arlington, Virginia, USA), and statistical inference was performed using RStudio (v4.2.1) (Boston, Massachusetts, USA). Tables and graphs were generated using GraphPad Prism (v9.5.0) (San Diego, California, USA). Odds ratios (OR), confidence intervals (CI) and p-values were computed by fitting single-variable logistic regression models to explore the effect of various demographic characteristics on students' career intentions. Confidence intervals were calculated at 95% level. We used $p<0.05$ to determine the statistical significance for all tests.

The findings of this study were reported in accordance with the STROBE (Strengthening the Reporting of Observational Studies in Epidemiology) guidelines (10).

Qualitative data analysis

Participants were asked to share which steps, if any, could be taken to improve the prospect of working in the NHS. The resulting qualitative data underwent inductive thematic analysis as per Braun and Clarke's method, with attention paid to reflexivity (11). By employing an inductive analysis approach, we conducted data analysis without predetermined themes. This approach offered the advantage of allowing the results to emerge directly from the data, ensuring a data-driven analysis. Upon familiarisation with the data, responses were imported into Microsoft Excel and individually labelled with an initial set of codes. These codes and the patterns identified were later used to generate themes, which were then arranged into larger overarching categories. To account for the inherent subjectivity in the interpretation of free-text data, members of the authorship team discussed points of uncertainty until a consensus was reached. To ensure the reliability and

For peer review only - http://bmjopen.bmj.com/site/about/guidelines.xhtml

consistency of the coding process, a subset of the data was independently coded by two authors. Any discrepancies in coding were discussed, and consensus was reached through team meetings to enhance intercoder reliability.

To address potential researcher bias, a reflexive approach was adopted. Regular team discussions were held to deliberate on differing interpretations and challenge potential biases. Additionally, the research team sought external input through peer review and feedback, engaging individuals familiar with the research topic. By actively engaging in reflexivity, critically examining our own assumptions, and incorporating external insights, we aimed to minimise the impact of personal biases and subjectivities on the qualitative data analysis process. These strategies ensured a rigorous and credible analysis of the qualitative data.

Patients and Public Involvement

None.

Results

Demographics

In total, 10,486 students across all 44 medical schools in the UK participated in the survey (Supplementary Figure 1). This represents approximately 21.50% of the medical student population in the UK. The mean response number per medical school was 244, and the median was 203 (IQR 135-281). A breakdown of the response numbers per medical school can be found in the Supplemental Materials. The median age for participants was 22 (IQR 20-23). Although responses were obtained from all year groups, there were relatively fewer responses from students in the 'Year 4 (not penultimate year)' category, due to a smaller number of students in intercalating courses or schools with six-year medical programmes, rather than the conventional five-year curriculum. Among the participants, 66.5% were female (n=6,977), 32.7% were male (n=3,429), 0.6% were non-binary (n=64), and 16 individuals preferred not to disclose their gender (*Table 1*).

Table 1 – Demographic characteristics of participants

Characteristic	Number (%)
Ethnicity	
White	5,838 (55.67)

For peer review only

Asian or Asian British	3,027 (28.87)
Black, Black British, Caribbean or African	529 (5.04)
Mixed or multiple ethnic groups	555 (5.29)
Other	410 (3.91)
Prefer not to say	127 (1.21)
Gender	
Female	6,977 (66.54)
Male	3,429 (32.70)
Non-binary	64 (0.61)
Prefer not to say	16 (0.15)
Level of education	
Postgraduate	1,873 (17.86)
Undergraduate	8,613 (82.14)
Previous schooling	
Private education	3,605 (34.38)
State education	6,609 (63.03)
Prefer not to say	272 (2.59)
Fee status	
Home	9,207 (87.80)
EU	419 (4.00)
International (Non-EU)	860 (8.20)
Current year of study	
Year 1	1,963 (18.72)
Year 2	2,152 (20.52)
Year 3	1,952 (18.62)
Year 4 (not penultimate year)	947 (9.03)
Penultimate Year	1,989 (18.97)

For peer review only - http://bmjopen.bmj.com/site/about/guidelines.xhtml

Final Year	1,483 (14.14)
Age	
Median (range)	22 (17-48)
Total	10,486 (100.00)

Career intentions

All participants were asked their current career intention for immediately after graduation, as shown in *Supplemental Table 1*. The majority of students (8,806/10,486, 83.98% (CI: 83.26%, 84.67%)) planned to complete both years of the UK's foundation training, Foundation Years 1 (FY1) and 2 (FY2); 10.50% (CI: 9.93%, 11.10%) intended to complete FY1 and then emigrate to practise medicine (n=1,101); 1.26% (CI: 1.06%, 1.49%) of students planned to complete FY1 and then permanently leave the profession (n=132); 0.99% (CI: 0.82%, 1.20%) of students intended to leave medicine permanently immediately after graduation (n=104); 2.10% (CI: 1.84%, 2.39%) of students planned to emigrate to practise medicine abroad immediately after graduation (n=220); and 1.17% (CI: 0.98%, 1.40%) of students intended to take a break or undertake further study post-graduation (n=123).

Participants intending to complete both years of the Foundation Programme were then asked their intentions thereafter; the results can be seen in *Supplemental Table 2*. Of these 8,806 respondents, 48.76% (n=4,294, CI: 47.72%, 49.81%) planned to enter specialty training in the UK immediately after the Foundation Programme; 21.11% (n=1,859, CI: 20.27%, 21.98%) intended to enter a non-training clinical job in the UK (a common form of 'F3' year, including posts such as junior clinical fellowship (JCF) or clinical teaching fellowship (CTF), or working as a locum doctor). These positions, while clinical in nature and valuable for gaining practical experience, do not typically contribute to full accreditation within a medical specialty, thus termed 'non-training'. A further 23.52% of students (n=2,071, CI: 22.64%, 24.42%) intended to emigrate to practise medicine abroad, whilst 5.85% (n=515, CI: 5.38%, 6.36%) planned to take a break or undertake further study. 67 students (0.76%, CI: 0.60%, 0.97%) planned to leave medicine permanently.

A total of 32.35% of medical students (n=3,392/10,486, CI: 31.46%, 33.25%) intended to emigrate to practise medicine, either immediately after graduation (n=220/3,292, 6.49%, CI: 5.71%, 7.36%), after completion of FY1 (n=1,101/3,292 32.46%, CI: 30.90%, 34.05%) or after FY2 (n=2,071/3,292, 61.06%, CI: 59.40%, 62.68%). These students were asked their likelihood of their return to UK medicine (return prospects); 49.56%

For peer review only - http://bmjopen.bmj.com/site/about/guidelines.xhtml

(n=1,681, CI: 47.88%, 51.24%) planned to return after a few years, whilst 7.87% (n=267, CI: 7.01%, 8.83%) intended to return after completion of their medical training abroad. The remaining 42.57% (n=1,444, CI: 40.92%, 44.24%) of students emigrating indicated no intentions to return (Supplemental Figure 2a). Of those favouring emigration immediately after graduation, 80.91% did not intend to return to the UK (n=178/220, CI: 75.20%, 85.55%). This number decreased to 60.03% (n=661/1101, CI: 57.11%, 62.89%)) in those emigrating after completing FY1, and 29.21% (n=605/2071, CI: 27.29%, 31.21%) in those emigrating after completing FY2, as demonstrated in Supplemental Figure 2b.

All students intending to emigrate to practise medicine were asked the countries to which they were considering emigrating via a free-entry text box. Students were able to list multiple locations or express if they were undecided. A total of 4,115 responses were received from 3,392 students. 25.03% of students (n=849) did not express a preference for any particular destination (Figure 1c). The remaining 2,543 medical students listed 3,266 destination preferences. Australia was the most commonly mentioned destination (42.35%), followed by New Zealand (18.03%), the United States (10.38%) and Canada (10.29%).

A total of 303/10,486 (2.89%, CI: 2.59%, 3.23%) of medical students planned to leave the profession entirely, either immediately after graduating (n=104/303, 34.32%, CI: 29.20%, 39.84%), after completion of FY1 (n=132/303, 43.56%, CI: 38.1%, 49.19%), or after completion of FY2 (n=67/303, 22.11%, CI: 17.8%, 27.12%). Students intending to leave the profession were asked the alternative industries they were considering for their future careers (Figure 1d). 21.12% (n=64/303) of those planning to leave the profession did not yet have an industry in mind. Of the remaining 78.88%, career destinations mentioned most often included consulting, technology, financial services and law.

(Insert Figures 1, a-d)

Career Intention Subanalyses

Subanalysis of career intentions after graduation by year of study revealed an overall increase in the proportion of students intending to complete the Foundation Programme as they progressed in their medical studies (*Supplemental Figure 3*). *Supplemental Tables 3 and 4* highlight students' career intentions after graduation and Foundation Programme, respectively, by year group.

Subanalysis of career intentions after completion of FY2 by current year of study revealed a significant decrease in the proportion of students looking to enter specialty training as they progressed in their medical studies (*Supplemental Table 4*). By contrast, student intentions to emigrate, permanently leave the profession and assume non-training clinical positions also increased as students advanced through medical school (*Figures 2, a-d*).

For peer review only - http://bmjopen.bmj.com/site/about/guidelines.xhtml

For peer review only

(Insert Figures 2, a-d)

Subanalysis of the subgroup intending to leave medicine (n=303, 2.89%) revealed a significant difference in the proportion of students taking this decision by various demographic characteristics, as highlighted in *Table 2*. Specifically, males were significantly more likely to leave medicine than females (OR 2.61, CI [2.08, 3.30], p<0.00001), and state-educated students had a higher likelihood of leaving medicine compared to privately educated students (OR 1.28, CI: [1.01, 1.62], p=0.04). However, no statistically significant difference between home students and non-home students, including international and non-EU students was identified (OR 1.26, CI [0.71, 2.06], p=0.39). Similarly, we did not find a statistically significant difference between undergraduates and postgraduates in their likelihood of leaving medicine (OR 1.29, CI [0.94, 1.80], p=0.124).

We subanalysed the group of students intending to emigrate to practise by ethnicity, gender, stage of training, educational background, and previous schooling (*Table 2)*. Males were significantly more likely to emigrate to practise medicine than females (OR 1.17, CI [1.07, 1.27], p<0.001). Postgraduate students were significantly more likely to emigrate to practise medicine than undergraduate students (OR 1.20, CI [1.08, 1.33], p<0.001). Privately educated students were significantly more likely to emigrate to practise medicine than their state educated peers (OR 1.26, CI [1.15, 1.37], p<0.00001). Non-home students (international and non-EU fees) were considerably more likely to emigrate to practise medicine than home students (OR 2.33, CI [1.92, 2.84], p<0.00001).

Table 2 – Demographic subanalysis of students intending to leave the medical profession and of students intending to emigrate to practise medicine.

Demographic subgroup	Number intending to leave medicine (%)
Ethnicity	
White	147 (2.52)
Asian or Asian British	99 (3.27)
Black, Black British, Caribbean or African	15 (2.84)
Mixed or multiple ethnic groups	24 (4.32)
Other	10 (2.44)
Prefer not to say	8 (6.30)

For peer review only - http://bmjopen.bmj.com/site/about/guidelines.xhtml

Gender	
Female	134 (1.92)
Male	167 (4.87)
Non-binary	1 (1.56)
Prefer not to say	1 (6.25)
Level of education	
Postgraduate	44 (2.35)
Undergraduate	259 (3.01)
Previous schooling	
Private education	118 (3.27)
State education	170 (2.57)
Prefer not to say	15 (5.51)
Fee status	
Home	276 (3.00)
EU	15 (3.58)
International (non-EU)	12 (1.40)
Current year of study	
Year 1	21 (1.07)
Year 2	42 (1.95)
Year 3	53 (2.72)
Year 4 (not penultimate year)	46 (4.86)
Penultimate year	75 (3.77)
Final year	66 (4.45)

Total	303 (100.00)

We also performed demographic subanalysis on students' likelihood to return to the UK if emigrating abroad (*Supplemental Table 5*). Males were significantly less likely to return to the UK after emigrating to practise medicine than females (OR 0.65, CI [0.56, 0.75], p<0.00001). Postgraduates were less likely to return to the UK after emigrating to practise medicine than undergraduates (OR 0.85, CI [0.71, 1.00], p=0.05). Privately educated students were significantly less likely to return to the UK after emigrating to practise medicine than state educated students (OR 0.77, CI [0.67, 0.89], p<0.001). Non-home students (international and EU fees) were significantly less likely to return to the UK after emigrating to practise medicine than home students (OR 0.18, CI [0.14, 0.23], p<0.00001).

Reasons for students' decisions and overall view of aspects of working in the NHS.

Once students had indicated their intended career option, they were asked the importance behind each of the factors below in their decision to do so. A series of Likert scale matrices were used, with options varying from 'Very important' to 'Not at all important'. The elements used in the matrices were compiled by the authors through a review of the literature, social media, and input from other clinicians. Students' reasons for leaving the NHS, either by emigrating or leaving the profession entirely, can be found in *Figures 1a and 1b*. For those not entering either the Foundation Programme or specialty training, immediately after completion of medical school or foundation training, burnout, and the ability to choose their working location were the most important factors in this decision. The full results can be found in the *Supplemental Figures 4 and 5*.

Remuneration at junior level, work-life balance, autonomy over choice of location and the working conditions of doctors in the NHS were identified as the most important factors for students intending to emigrate to practise medicine (Figure 2a). Similarly, this was also the case for those leaving medicine, with the addition of nearly 82% of medical students listing burnout as an important or very important reason to abandon the profession (*Figure 2b*).

To better ascertain the medical student population's overview of working in the NHS, students were asked to share their degree of satisfaction with several aspects of working in the NHS. Likert scale matrices were again used in a similar fashion, with options ranging from 'Very satisfied' to 'Not at all satisfied'. *Supplemental Figure 6* highlights this. Less than 6% of the medical student population reported feeling satisfied or very satisfied with remuneration at junior level, work-life balance, working conditions of a doctor in the NHS, and costs associated with training (such as charges for memberships and examinations). A sizeable proportion of

For peer review only - http://bmjopen.bmj.com/site/about/guidelines.xhtml

participants responded with a neutral rating, neither satisfied nor unsatisfied, when asked about several aspects of their prospective medical training. Specifically, these aspects included pension tax rules as a consultant, theatre time during the Foundation Programme, and exposure to their desired specialty during the foundation programme. In cases where participants may not have held strong opinions on a particular aspect, they tended to select the neutral option. Only 17.26% of students were satisfied or very satisfied with the overall prospect of working in the NHS.

Thematic analysis

Improving the prospect of working in the NHS

In total, 10,486 survey responses were collected, of which 5,294 students provided qualitative data by answering the optional question 'What steps could be taken to improve the prospect of working in the NHS?', resulting in a response rate of 50.47%. The qualitative data revealed a wide range of responses in terms of both length and content, which were subjected to thematic analysis. The majority of responses could be grouped into at least one of six distinct categories. Most of the responses alluded to concepts present in multiple categories and so were counted in all which applied. Notably, a small proportion of responses (2.08%, n=110) were deemed too broad, too vague, unintelligible, or otherwise impossible to categorise and were grouped into a separate category labelled as "Other".

"Financial considerations" were the most commonly cited area for improvement (n=4,284, 80.92%), encompassing a desire for greater remuneration, changes to pension policy, and a reduction in mandatory fees incurred by working as a doctor. A substantial proportion of those advocating for enhanced remuneration made specific reference to the concept of "pay restoration": a reversal of any real terms decline in pay faced by doctors. Concepts relating to "Working in the NHS" were also extremely prevalent (n=4,102, 77.48%), generally focusing on improved work-life balance and working conditions in the health service.

Responses to which the label of "Training and practice" was attributed (n=1,745, 32.96%) chiefly focused on autonomy of working location, with many opposing the current system of rotational training, in which doctors' working location is changed on a semi-regular basis throughout their training. This may involve regularly moving between hospital departments or to different hospitals. The issues of accessibility, quality, and streamlining – generally, shortening – of postgraduate training programmes also arose quite often. Some were dissatisfied with the presence of tasks seen to constitute "service provision" during training programmes, such as performing phlebotomy and writing patient discharge summaries, in the place of dedicated teaching or training opportunities. A number of respondents proposed changes to the current application and allocation processes for foundation and specialty training programmes. Recurring ideas were a reduced emphasis on portfolio-building and the phasing-out of centralised, national applicant ranking methods.

For peer review only - http://bmjopen.bmj.com/site/about/guidelines.xhtml

In the "NHS and society" category (n=1,672, 31.58%), perceived insufficient staffing levels and a desire for increased NHS funding were the most noteworthy topics of discussion. Some described frustration with current NHS management structures and systemic inefficiencies, at times advocating for a thinning of middle-management along with a greater role for doctors and other clinicians in the system's operation. A minority suggested changes in government leadership and current political circumstances might enhance the prospect of working in the NHS. Interestingly, there were some conflicting views among our respondents, with one group backing the reversal of perceived NHS privatisation and a guarantee that it will continue to exist in its current form. The opposing, and similar sized, group promoted increasing levels of privatisation and, occasionally, introducing a fee-for-service model.

Slightly less common were responses which were grouped into the "Culture and support" category (n=994, 18.78%), in which perceptions of insufficient support, and inadequate rights, respect, and treatment of doctors and allied health professionals led the argument. Those who considered the latter mentioned a sense of these roles and the individuals who fill them not being valued or shown the respect they deserve. Finally, "Medical school and education" was the category cited least often by our respondents (n=194, 3.66%), with primary concerns relating to university degree funding and debt, and a lack of early career guidance.

Table 3 presents the categories and their corresponding themes in full, along with the number of responses that corresponded to each theme.

Table 3 – Thematic analysis of students' suggestions for improving the prospect of working in the NHS. *AHPs = allied health professionals, e.g., physician's associate and advanced clinical practitioners.

Key themes generated	Number of mentions	Percentage of students
Financial considerations	*4,284*	*80.92%*
Remuneration	4,080	*77.07%*
Fees incurred by medical practice, e.g., examinations, courses	155	*2.93%*
Pension	49	*0.93%*

For peer review only - http://bmjopen.bmj.com/site/about/guidelines.xhtml

Working in the NHS	*4,102*	*77.48%*
Work-life balance, rotas, and flexibility	*1,749*	*33.04%*
Working conditions	*1,389*	*26.24%*
Levels of stress, responsibility, and pressure	*337*	*6.37%*
Breaks, leave and non-clinical opportunities	*214*	*4.04%*
Resources, equipment, technology, and facilities	*205*	*3.87%*
Incentives, benefits and perks, e.g., parking, accommodation, etc	*203*	*3.83%*
Visa status and citizenship	5	*0.09%*
Training and practice	*1,745*	*32.96%*
Autonomy of working location and reduction in rotational training	*525*	*9.92%*
Levels of competition for foundation posts, specialty training posts, and consultant posts	*446*	*8.42%*
Quality of training and teaching	*282*	*5.33%*
Streamlining foundation and specialty training	*174*	*3.29%*
"Service provision", non-clinical responsibilities, and bureaucracy	*126*	*2.38%*
Postgraduate training application and allocation processes	124	*2.34%*
Regulation of AHPs* and prioritisation of doctors' training and tasks	46	*0.87%*
Variety and degree of specialty exposure before training application	22	*0.42%*
The NHS and society	*1,672*	*31.58%*
Staffing levels	850	*16.06%*
Funding of the NHS, social care, and other health services	258	*4.87%*
Operational reform, efficiency, and reduction in non-clinical middle management	174	*3.29%*

Bed availability, waiting lists, appointment duration and patient experience	125	*2.36%*
Prioritising mental well-being of NHS staff	105	*1.98%*
Changes in government leadership	64	*1.21%*
Addressing discrimination in the NHS	36	*0.68%*
Reversal of privatisation of the NHS and assurance of NHS' longevity	32	*0.60%*
Increased privatisation of the NHS	28	*0.53%*
Culture and support	*994*	*18.78%*
Treatment and respect for doctors and AHPs	371	*7.01%*
Support for doctors and AHPs	306	*5.78%*
Workplace culture	224	*4.23%*
Staff morale	63	*1.19%*
Autonomy of practice, litigation, and the GMC	30	*0.57%*
Medical school and education	*194*	*3.66%*
Career, portfolio, and specialty application guidance	78	*1.47%*
University degree funding and debt	71	*1.34%*
Quality, design, and conditions of medical school education	24	*0.45%*
Medical school places and changes to entry requirements	15	*0.28%*
Degree length and flexibility	6	*0.11%*
Other	*110*	*2.08%*
Vague, uninterpretable, or otherwise uncategorisable	*110*	*2.08%*
Total	*5,294*	*100.00%*

Discussion

Principal findings

Our findings highlight that a high proportion of participating medical students intend to either leave the profession or permanently emigrate to practise medicine. There are no previous studies to which to compare these results, so it is hard to gauge how these figures may have changed over time. We have observed that with each successive year of medical school, students become less inclined to enter specialty training in the UK without a break, or at all. Specifically, less than a quarter of final-year medical students intend to enter specialty training immediately after the Foundation Programme. In total, 35.23% of medical students plan to leave the NHS within two years of graduating, either to practise abroad or to pursue other careers. Approximately 60% of the medical student population in the UK is either not satisfied or not at all satisfied with the prospect of working in the NHS.

Implications

The NHS is facing a critical workforce shortage, with approximately 10,000 doctors relinquishing their licence to practise in 2021, representing a loss of nearly one-tenth of the doctor workforce (5, 12). A British Medical Association (BMA) survey of 8,000 senior doctors determined that 44% of NHS consultants in England plan to leave or take a break from working in the NHS over the next year (13). Similarly, a recent survey of 4,553 junior doctors in the NHS reported that 4 in 10 plan to leave the NHS, with 33% of these wanting to emigrate to another country to work (14). The combination of these previous surveys of the doctor workforce, and the results of our medical student survey suggest this trend is presently unlikely to improve. The GMC has recognised the problem and called for immediate action to mitigate the exodus of doctors from the NHS to more attractive employers (15).

Countries within the anglosphere, namely Australia, New Zealand, the United States and Canada, were the most widely cited destinations for students intending to emigrate. This is perhaps unsurprising given the higher salaries, reports of improved work-life balance, and the fact that these countries' primary language is English (16). Our study's findings align with previous literature highlighting doctors' reasons for emigration, namely pay, working conditions, and work-life balance (1, 17).

This study highlights that a disconcerting proportion of participating students, 32.35% (CI: 31.46%, 33.25%), intend to emigrate to practise medicine, with nearly half of these students intending not to return. This represents a large proportion of the medical student cohort. Despite these figures, there remains great uncertainty in this area. It is important to note that a considerable number of students who initially express an

For peer review only - http://bmjopen.bmj.com/site/about/guidelines.xhtml

intention to emigrate temporarily may ultimately choose to stay abroad permanently (17). Similarly, some students who do not intend to return to the UK may change their minds in the future. Students paying EU or international fees reported significantly higher intentions to emigrate permanently. The stage at which students intend to emigrate appears to be related to the likelihood of return. Notably, our study suggests that the proportion of students who intend to leave the NHS may be underestimated, as more students express a desire to leave as they progress through medical school. Moreover, once students enter the Foundation Program, a proportion may decide to leave the NHS, even if they had not previously intended to do so.

Insights into the emigration intentions of medical students in other nations indicate that a substantial proportion express a desire to emigrate and practise medicine in countries such as the United Kingdom, the United States of America, and Canada. For instance, in a study, it was found that 49.7% of Malagasy medical students and 25.2% of Tanzanian medical students expressed their intention to emigrate to practise (18). Similarly, in another study, it was revealed that 44.6% of Ugandan medical students planned to emigrate (19). These findings highlight the significant interest among medical students from these countries to pursue opportunities abroad.

Our results indicate that 2.89% of the medical students participating in our study expressed intentions to quit medicine. In a study conducted in Kazakhstan, a similar trend was observed with 4% of the participants expressing a desire to leave the medical profession (20). Notably, similar to our results, the study reported a pattern in which medical students in junior years were less inclined to express a desire to leave the profession compared to students in senior years (20).

In addition to the 35.24% of sampled medical students intending to quit the NHS within two years of graduating, a considerable proportion of students (21.11%, CI: 20.27%, 21.98%) intend to assume a non-training clinical position in the UK after completing the Foundation Programme. Participants reported motivations for working in a non-training clinical post in keeping with existing literature surrounding the 'F3' year, with burnout, the ability to choose work location, travel and a greater earning potential being the most compelling reasons to do so (21, 22). Furthermore, in this aspect, we report an increase in intention to not take up specialty posts immediately after the Foundation Programme, with an increase from 6.75% (CI: 5.62%, 8.08%) of first-year students to 35.98% (CI: 33.45%, 38.59%) of final year students. A contributing factor to this scenario could be a significant increase in competition ratios for specialty training posts, partly due to increasing medical student places and no corresponding increase in the number of training posts available (for example, neurosurgery ST1 competition ratio was 3.9 in 2013 vs 15.94 in 2022) (23). Without corresponding increases to specialist training posts, increases in medical school places may be ineffective in doctor retention.

Historically, the vast majority of medical graduates pursued specialty training immediately after completing their Foundation Programme; for instance, in 2010, 83.1% of doctors entered specialty training after

For peer review only - http://bmjopen.bmj.com/site/about/guidelines.xhtml

completing FY2. However, after steadily decreasing year-on-year, this percentage was only 34.9% of doctors in 2019 (7). The UK Foundation Programme Office has not repeated the survey since then, so surmising how these statistics may have changed in the interim is difficult. Our findings report that under half of medical students intend to enter specialty training after the Foundation Programme, with a negative correlation between medical student seniority and intention to enter specialty training with no break, or at all. Only 25.80% of final-year students intend to do so. In the UKFPO survey, those doctors had experienced the negative aspects of the profession. As such, it is concerning to observe this decline in interest among medical students, who have yet to formally begin their career in medicine.

The findings of our study align with existing literature on the factors influencing junior doctors' career decisions. Consistently, previous research emphasises the significance of working conditions, location, and earnings in shaping these decisions (1, 2, 21, 22, 24-27). Challenging work environments, long hours, and inadequate support contribute to disillusionment, burnout, and a desire to pursue alternative career paths (28). Similarly, the autonomy to choose work location emerges as a key factor in medical students, echoing findings among junior doctors. Earnings have consistently been identified as an influential factor for both junior doctors and medical students (1, 2, 16, 17, 21-27). Financial considerations impact their quality of life, student loan repayments, and long-term financial stability. The allure of higher salaries and better earning potential in other healthcare systems or professions can attract medical graduates away from NHS training programs. Addressing working conditions, providing career advancement opportunities, ensuring internationally competitive salaries, and considering location preferences can improve the ability to attract and retain talented professionals. Our study contributes to the growing body of literature by including medical students and supporting the notion that working conditions, location, and earnings are significant factors influencing junior doctors' decisions to enter or remain in training. These findings underscore the importance of addressing these factors to create a supportive and appealing environment for junior doctors, ultimately promoting better retention rates within the NHS.

Furthermore, our results suggest that the recent calls for dramatic increases in medical school places are unlikely to resolve the NHS staffing shortages. The MSC has responded to the original call to increase places by 5,000 students by stating multiple barriers, including cost, clinical placement capacity and the lack of a strategic approach to growth. It is estimated that to increase medical schools' capacity by just 5,000 places, approximately £1 billion per annum would be required (29). Additionally, the training of medical students heavily relies on clinical exposure, which in turn is dependent on availability of clinical teaching staff, facilities for training and opportunities (6). Without a corresponding increase in clinical placement capacity, an increase in medical student places may lead to a decrease in the standard of medical education. Our results indicate that increases in medical student places via expansion of existing medical schools or the establishment of new medical schools may not result in proportionate increases in doctors wishing to remain in the NHS. Any attempts to reverse the NHS workforce challenge may benefit from prioritising doctor retention. Here we

For peer review only - http://bmjopen.bmj.com/site/about/guidelines.xhtml

have highlighted the reasons driving medical students to plan for careers outside of the NHS; addressing these problems is likely to result in improved retention rates.

While there have been studies which i) explore which specialties junior doctors or medical students intend on pursuing, and exploring factors attracting them to said specialties (30-52); ii) focus on reasons why doctors are leaving the UK(1, 2, 24, 53); iii) exploring how medical students and junior doctors feel about specific aspects of working within the NHS (25-27, 54), and iv) studies investigating the desire for a career break post-FY2 (21, 22), there have been no recent, high-powered studies explicitly aimed at medical students, irrespective of current career ambitions or seniority, investigating overall career intentions and correlating it with demographic factors and medical student seniority. Any statistically significant differences in career intentions between demographic subgroups should be considered carefully and discussed within the correct context. Further studies are required to fully elucidate the reasons behind these disparities.

Limitations

When interpreting this study's results, there are important limitations to consider. Firstly, the study's cross-sectional nature means we are unable to gauge how students' career intentions may have changed or will change. To address this, we have asked all participants for consent to participate in an anticipated follow-up study which will enable validation of responses and measurement of change over time; to this, we obtained a 71.29% consent rate.

While this study represents the largest ever study of UK medical students, it is worth noting that approximately 21.50% of the total population of medical students participated. Consequently, we cannot exclude the possibility of selection bias. It may be that this survey appealed to those already intending to leave the NHS or are interested in this topic. In the context of the UK's medical student population, females were overrepresented in our study despite concerted efforts to ensure equitable outreach during our study advertising phase. While steps were taken to reduce bias in the interpretation of the study's qualitative data, inherent subjectivity remains a limitation.

Additionally, the questions in our survey instruct students to be definitive even when they might not yet have an idea of their career plans, particularly for those in the younger years of medical school. Finally, it should be emphasised that the respondents were medical students who may have limited knowledge of the realities of working in the NHS. Their current reported perceptions may change once they begin their career in the NHS.

Conclusion

For peer review only - http://bmjopen.bmj.com/site/about/guidelines.xhtml

This study highlights that an alarming proportion of medical students intend to leave the profession or emigrate to practise medicine. The proportion of students who plan to leave the NHS within two years of graduating is considerable, representing a potential loss of valuable medical talent. Alarmingly, the majority of medical students are either not at all satisfied or not satisfied with the prospect of working in the NHS. Additionally, an increasing proportion of students intend to take up non-training clinical positions, which could reduce the availability of highly skilled doctors in the NHS. The findings of this study underscore the urgency of addressing the factors that are driving the exodus of doctors from the NHS and suggest that increased recruitment of medical students may not provide an adequate solution to the staffing challenges. The causes of the problem are complex, and finding a solution will require a multifaceted approach. Steps could include improving work-life balance, increasing salaries, addressing the growing competition for training posts, and promoting greater flexibility in career pathways. Undoubtedly, the continued loss of skilled professionals from the NHS represents a significant concern, so it Is critical to consider means of reversing this trend.

For peer review only

Notes

Authors' contributions

TF responsible for conceptualisation. TF responsible for obtaining funding and ethical approval. TF responsible for collaborator recruitment and management. TF responsible for project administration. TF and AMC responsible for designing the survey. TF responsible for writing the manuscript. TF and AMC responsible for qualitative analysis. OF and RJS responsible for statistical quantitative analysis. All authors responsible for editing and revising the manuscript. RH responsible for supervision. TF is the guarantor. All authors have read and approved the manuscript.

Collaborators

Mario K Teo, Crispin C Wigfield, Dania Al-Hashimi, Maeve K Mulchrone, Alisha Pervaiz, Heather A Lewis, Anson Wong, Buzz Gilks, Charlotte Casteleyn, Sara Kidher, Erin Fitzsimons-West, Tanzil Rujeedawa, Meghna Sreekumar, Eliza Wade, Juel Choppy-Madeleine, Yasemin Durmus, Olivia King, Yu Ning Ooi, Malvi Shah, Tan Jih Yih, Samantha Burley, Basma R Khan, Emma Slack, Rishik S Pilla, Jenny Yang, Vaishvi Dalal, Brennan L Gibson, Emma Westwood, Brandon S H Low, Sara R Sabur , Wentin Chen, Maryam A Malik, Safa Razzaq, Amardeep Sidki, Giulia Cianci, Felicity Greenfield, Sajad Hussain, Alexandra Thomas, Annie

For peer review only - http://bmjopen.bmj.com/site/about/guidelines.xhtml

Harrison, Hugo Bernie, Luke Dcaccia, Linnuel J Pregil, Olivia Rowe, Ananya Jain, Gregory K Anyaegbunam, Syed Z Jafri, Arthur Handscomb, Sudhanvita Arun, Alfaiya Hashmi, Ankith Pandian, Joseph R Nicholson, Hannah Layton-Joyce, Kouther Mohsin, Matilda Gardener, Eunice C Y Kwan, Emily R Finbow, Sakshi Roy, Zoe M Constantinou, Mackenzie Garlick, Clare L Carney, Samantha Gold, Bilal Qureshi, Daniel Magee, Grace Annetts, Khyatee Shah, Kholood T Munir, Timothy Neill, Gurpreet K Atwal, Anesu Kusosa, Anthony Vijayanathan, Mia Mäntylä, Momina Iqbal, Sara Raja, Tushar Rakhecha, Muhammad H Shah, Pranjil Pokharel, Ashna Anil, Kate Stenning, Katie Appleton, Keerthana Uthayakumar, Rajan Panacer, Yasmin Owadally, Dilaxiha Rajendran, Harsh S Modalavalasa, Marta M Komosa, Morea Turjaka, Sruthi Saravanan, Amelia Dickson, Jack M Read, Georgina Cooper, Wing Chi Do, Chiamaka Anthony-Okeke, Daria M Bageac, David C W Loh, Rida Khan, Ruth Omenyo, Aidan Baker, Imogen Milner, Kavyesh Vivek, Manon Everard, Wajiha Rahman, Denis Chen, Michael E. Bryan, Shama Maliha, Vera Onongaya, Amber Dhoot, Catherine L Otoibhi, Harry Donkin-Everton, Mia K Whelan, Claudia S F Hobson, Anthony Haynes, Joshua Bayes-Green, Mariam S Malik, Subanki Srisakthivel, Sophie Kidd, Alan Saji, Govind Dhillon, Muhammed Asif, Riya Patel, Jessica L Marshall, Nain T Raja, Tawfique Rizwan, Aleksandra Dunin-Borkowska, James Brawn, Karthig Thillaivasan, Zainah Sindhoo, Ayeza Akhtar, Emma Hitchcock, Kelly Fletcher, Lok Pong Cheng, Medha Pillaai, Sakshi Garg, Wajahat Khan, Ben Sweeney, Ria Bhatt, Madison Slight, Adan M I Chew, Cameron Thurlow, Kriti Yadav, Niranjan Rajesh, Nathan-Dhruv Mistry, Alyssa Weissman, Juan F E Jaramillo, William Thompson, Gregor W Abercromby, Emily Gaskin, Chloe Milton, Matthew Kokkat, Momina Hussain, Nana A. Ohene-Darkoh, Syeda T Islam, Anushruti Yadav, Eve Richings, Samuel Foxcroft, Sukhdev Singh, Vivek Sivadev, Guilherme Movio, Ellena Leigh, Harriet Charlton, James A Cairn, Julia Shaaban , Leah Njenje, Mark J Bishop, Humairaa Ismail, Sarah L Henderson, Daniel C Chalk, Daniel J Mckenna, Fizah Hasan, Kanishka Saxena , Iona E Gibson & Saad Dosani.

Transparency declaration

TF, the lead author (the manuscript's guarantor), affirms that the manuscript is an honest, accurate, and transparent account of the study being reported; that no important aspects of the study have been omitted; and that any discrepancies from the study have been explained.

Ethics approval and consent to participate.

Ethical approval was granted by the University of Cambridge Research Ethics Committee (reference PRE.2022.124) on the [5]th of January 2023.

Consent for publication

The Corresponding Author has the right to grant on behalf of all authors and does grant on behalf of all authors, an exclusive licence (or non-exclusive for government employees) on a worldwide basis to the BMJ Publishing Group Ltd to permit this article (if accepted) to be published in BMJ editions and any other BMJPGL products and sublicences such use and exploit all subsidiary rights, as set out in our licence.

Competing interests

For peer review only - http://bmjopen.bmj.com/site/about/guidelines.xhtml

All authors have completed the Unified Competing Interest form (available on request from the corresponding author) and declare: no support from any organisation for the submitted work; no financial relationships with any organisations that might have an interest in the submitted work in the previous three years, no other relationships or activities that could appear to have influenced the submitted work.

Funding

Queens' College, University of Cambridge. The institution has had no role in the design of the study, nor collection, analysis, and interpretation of data and in writing the manuscript.

Acknowledgements

We would like to thank all students that participated in this study. We would also like to thank Mr Mario K Teo and Mr Crispin C Wigfield for their advice in the earlier stages of the study.

Author'' information (optional)

Not applicable.

Data availability

No additional data available.

For peer review only

Figures

Figures 1.a-d - **a)** importance of factors influencing medical students' intention to emigrate and practise medicine; **b)** importance of factors influencing medical students' intention to leave the medical profession entirely and seek an alternative career; **c)** locations cited as potential destinations by students who intend to emigrate to practise medicine.; **d)** preferred industries to work in by those intending to leave medicine. *Several respondents cited the Middle East or Gulf region rather than specifying which country; these responses were grouped with individual destinations in the region

Figures 2.a-d - Proportions of students by year of study (with 95% confidence intervals) intending to **a)** directly enter specialty training after FY2; **b)** emigrate to practise medicine after FY2; **c)** enter a non-training clinical post after FY2, for example as a locum doctor or clinical fellow; **d)** leave medicine permanently after FY2 to pursue an alternative career. "Year 4" represents students in their fourth year of study, but not their penultimate year. Percentages in figures reflect the proportion of students in each year group for each intention.

Bibliography

1. Lambert TW, Smith F, Goldacre MJ. Why doctors consider leaving UK medicine: qualitative analysis of comments from questionnaire surveys three years after graduation. Journal of the Royal Society of Medicine. 2018;111(1):18-30.
2. Surman G, Goldacre MJ, Lambert TW. UK-trained junior doctor'' intentions to work in UK medicine: questionnaire surveys, three years after graduation. Journal of the Royal Society of Medicine. 2017;110(12):493-500.
3. OECD (2023), Doctors (indicator). doi: 10.1787/4355e1ec-en (Accessed on 15 April 2023), Available from: https://data.oecd.org/healthres/doctors.htm
4. Rimmer A. Five medical schools are created in England in bid to increase home grown doctors. BMJ: British Medical Journal (Online). 2018;360.
5. General Medical Council. The state of medical education and practice in the UK. The workforce report 2022.
6. Phillips S MI. A roadmap to double medical school places. Dec 2022.
7. UKFPO. UK Foundation Programme 2019 F2 Career UKFPO 2019 F2 Career Destinations Survey. 2019.
8. De Souza B. Choosing your specialty Foundation training. BMJ. 2007;334(7601):s172-s3.
9. Ferreira T, Collins AM, Horvath R. Ascertaining the Career Intentions of Medical Students (AIMS) in the United Kingdom Post Graduation: Protocol for a Mixed Methods Study. JMIR Research Protocols. 2023 Jun 19;12(1):e45992.
10. Elm Ev. The Strengthening the Reporting of Observational Studies in Epidemiology (STROBE) statement: guidelines for reporting observational studies. Ann Inter Med. 2007;147:573-7.

For peer review only - http://bmjopen.bmj.com/site/about/guidelines.xhtml

11. Braun V, Clarke V. Using thematic analysis in psychology. Qualitative research in psychology. 2006;3(2):77-101.
12. NHS Digital Health. NHS Workforce Statistics, December 2021 Doctors by Grade and Specialty. 2022.
13. BMA. Catastrophic crisis facing NHS as nearly half of hospital consultants plan to leave in next year, warns BMA2022. Available from: https://www.bma.org.uk/bma-media-centre/catastrophic-crisis-facing-nhs-as-nearly-half-of-hospital-consultants-plan-to-leave-in-next-year-warns-bma.
14. Waters A. A third of junior doctors plan to leave NHS to work abroad in next 12 months. British Medical Journal Publishing Group; 2022.
15. General Medical Council. Migration and the medical workforce 2022 [Available from: https://www.gmc-uk.org/news/news-archive/migration-and-the-medical-workforce.
16. Grant P. Physician job satisfaction in New Zealand versus the United Kingdom. The New Zealand Medical Journal (Online). 2004;117(1204).
17. Sharma A, Lambert TW, Goldacre MJ. Why UK-trained doctors leave the UK: cross-sectional survey of doctors in New Zealand. Journal of the royal society of medicine. 2012;105(1):25-34.
18. Chaet A, Fessehaie N, Rajaguru PR, Alavi Jusabani M, Randaoharison P, Samison L, Anderson U, Ramanantoanina P, Zafimar M, Numfor A, Hardaker WM. Comparing the drivers of medical student emigration intention across two African nations. Medical Education. 2021 Oct;55(10):1194-204.
19. Kizito S, Mukunya D, Nakitende J, Nambasa S, Nampogo A, Kalyesubula R, Katamba A, Sewankambo N. Career intentions of final year medical students in Uganda after graduating: the burden of brain drain. BMC medical education. 2015 Dec;15:1-7.
20. Faizullina K, Kausova G, Kalmataeva Z, Nurbakyt A, Buzdaeva S. Career intentions and dropout causes among medical students in Kazakhstan. Medicina. 2013 Jun;49(6):45.
21. Church HR, Agius SJ. The F3 phenomenon: Early-career training breaks in medical training. A scoping review. Medical Education. 2021;55(9):1033-46.
22. Hollis AC, Streeter J, Van Hamel C, Milburn L, Alberti H. The new cultural norm: reasons why UK foundation doctors are choosing not to go straight into speciality training. BMC Medical Education. 2020;20(1):1-9.
23. NHS Health Education England. Competition Ratios [Available from: https://medical.hee.nhs.uk/medical-training-recruitment/medical-specialty-training/competition-ratios].
24. Milner A, Nielsen R, Verdery AM. Brexit and the European National Health Service England Workforce: A Quantitative Analysis of Doctors' Perceived Professional Impact and Intentions to Leave the United Kingdom. Annals of global health. 2021;87(1).
25. Scanlan GM, Cleland J, Johnston P, Walker K, Krucien N, Skåtun D. What factors are critical to attracting NHS foundation doctors into specialty or core training? A discrete choice experiment. BMJ open. 2018;8(3):e019911.
26. Ryan C, Ward E, Jones M. Recruitment and retention of trainee physicians: a retrospective analysis of the motivations and influences on career choice of trainee physicians. QJM: An International Journal of Medicine. 2018;111(5):313-8.
27. Cleland JA, Johnston P, Watson V, Krucien N, Skåtun D. What do UK medical students value most in their careers? A discrete choice experiment. Medical Education. 2017;51(8):839-51.
28. House of Commons Health and Social Care Committee. (2021). Workforce burnout and resilience in the NHS and social care: Second Report of Session 2021–22 (Publication No. HC 22). [Available from: https://committees.parliament.uk/publications/6158/documents/68766/default/] Accessed July 8, 2023.
29. Medical Schools Council. The expansion of medical student numbers in the United Kingdom. 2021.
30. Singh A, Alberti H. Why UK medical students change career preferences: an interview study. Perspectives on medical education. 2021;10(1):41-9.
31. Misky AT, Shah RJ, Fung CY, Sam AH, Meeran K, Kingsbury M, et al. Understanding concepts of generalism and specialism amongst medical students at a research-intensive London medical school. BMC Medical Education. 2022;22(1):1-11.
32. Lambert TW, Smith F, Goldacre MJ. Career specialty choices of UK medical graduates of 2015 compared with earlier cohorts: questionnaire surveys. Postgraduate Medical Journal. 2018;94(1110):191-7.
33. Surman G, Lambert TW, Goldacre MJ. Trends in junior doctors' certainty about their career choice of eventual clinical specialty: UK surveys. Postgraduate medical journal. 2013;89(1057):632-7.
34. Svirko E, Goldacre MJ, Lambert T. Career choices of the United Kingdom medical graduates of 2005, 2008 and 2009: questionnaire surveys. Medical teacher. 2013;35(5):365-75.
35. Ibrahim M, Fanshawe A, Patel V, Goswami K, Chilvers G, Ting M, et al. What factors influence British medical students' career intentions? Medical teacher. 2014;36(12):1064-72.
36. Reid K, Alberti H. Medical students' perceptions of general practice as a career; a phenomenological study using socialisation theory. Education for Primary Care. 2018;29(4):208-14.

For peer review only - http://bmjopen.bmj.com/site/about/guidelines.xhtml

37. Rehman U, Sarwar MS, Brennan PA. Attitude of clinical medical students to Oral and Maxillofacial Surgery as a career: a perspective from two English Medical Schools. British Journal of Oral and Maxillofacial Surgery. 2022;60(4):448-53.
38. Barber S, Brettell R, Perera-Salazar R, Greenhalgh T, Harrington R. UK medical students' attitudes towards their future careers and general practice: a cross-sectional survey and qualitative analysis of an Oxford cohort. BMC medical education. 2018;18(1):1-9.
39. Oliver H, Hudson B, Oliver C, Oliver M. UK undergraduate aspirations and attitudes survey: do we have a perception problem in clinical radiology? Clinical Radiology. 2020;75(2):158. e15-. e24.
40. Emmanouil B, Goldacre MJ, Lambert TW. Aspirations to become an anaesthetist: longitudinal study of historical trends and trajectories of UK-qualified doctors' early career choices and of factors that have influenced their choices. BMC anesthesiology. 2017;17(1):1-9.
41. Barat A, Goldacre MJ, Lambert TW. Junior doctors' early career choices do not predict career destination in neurology: 40 years of surveys of UK medical graduates. BMC medical education. 2019;19(1):1-9.
42. Robinson T, Lefroy J. How do medical students' experiences inform their opinions of general practice and its potential as a future career choice? Education for Primary Care. 2022:1-9.
43. Tambyraja AL, McCrea CA, Parks RW, Garden OJ. Attitudes of medical students toward careers in general surgery. World journal of surgery. 2008;32(6):960-3.
44. Thomas A. What about forensic psychiatry as a career? Undergraduate and early post-graduate medical perspectives. Criminal Behaviour and Mental Health. 2012;22(4):247-51.
45. Smith F, Lambert TW, Pitcher A, Goldacre MJ. Career choices for cardiology: cohort studies of UK medical graduates. BMC medical education. 2013;13(1):1-8.
46. Maisonneuve JJ, Pulford C, Lambert TW, Goldacre MJ. Career choices for geriatric medicine: national surveys of graduates of 1974–2009 from all UK medical schools. Age and ageing. 2014;43(4):535-41.
47. Goodson AM, Payne KF, Tahim A, Cabot L, Fan K. Awareness of oral and maxillofacial surgery as a specialty and potential career pathway amongst UK medical undergraduates. The Surgeon. 2013;11(2):92-5.
48. Halder N, Hadjidemetriou C, Pearson R, Farooq K, Lydall GJ, Malik A, et al. Student career choice in psychiatry: findings from 18 UK medical schools. International Review of Psychiatry. 2013;25(4):438-44.
49. Goldacre MJ, Fazel S, Smith F, Lambert T. Choice and rejection of psychiatry as a career: surveys of UK medical graduates from 1974 to 2009. The British Journal of Psychiatry. 2013;202(3):228-34.
50. Pakpoor J, Handel AE, Disanto G, Davenport RJ, Giovannoni G, Ramagopalan SV. National survey of UK medical students on the perception of neurology. BMC medical education. 2014;14(1):1-5.
51. Sutton PA, Mason J, Vimalachandran D, McNally S. Attitudes, motivators, and barriers to a career in surgery: a national study of UK undergraduate medical students. Journal of surgical education. 2014;71(5):662-7.
52. Moore J, McDiarmid A, Johnston P, Cleland J. Identifying and exploring factors influencing career choice, recruitment and retention of anaesthesia trainees in the UK. Postgraduate medical journal. 2017;93(1096):61-6.
53. Wilson HC, Abrams S, Simpkin Begin A. Drexit: Understanding why junior doctors leave their training programs to train overseas: An observational study of UK physicians. Health Science Reports. 2021;4(4):e419.
54. Lachish S, Goldacre MJ, Lambert T. Associations between perceived institutional support, job enjoyment, and intentions to work in the United Kingdom: national questionnaire survey of first year doctors. BMC medical education. 2016;16(1):1-8.

For peer review only

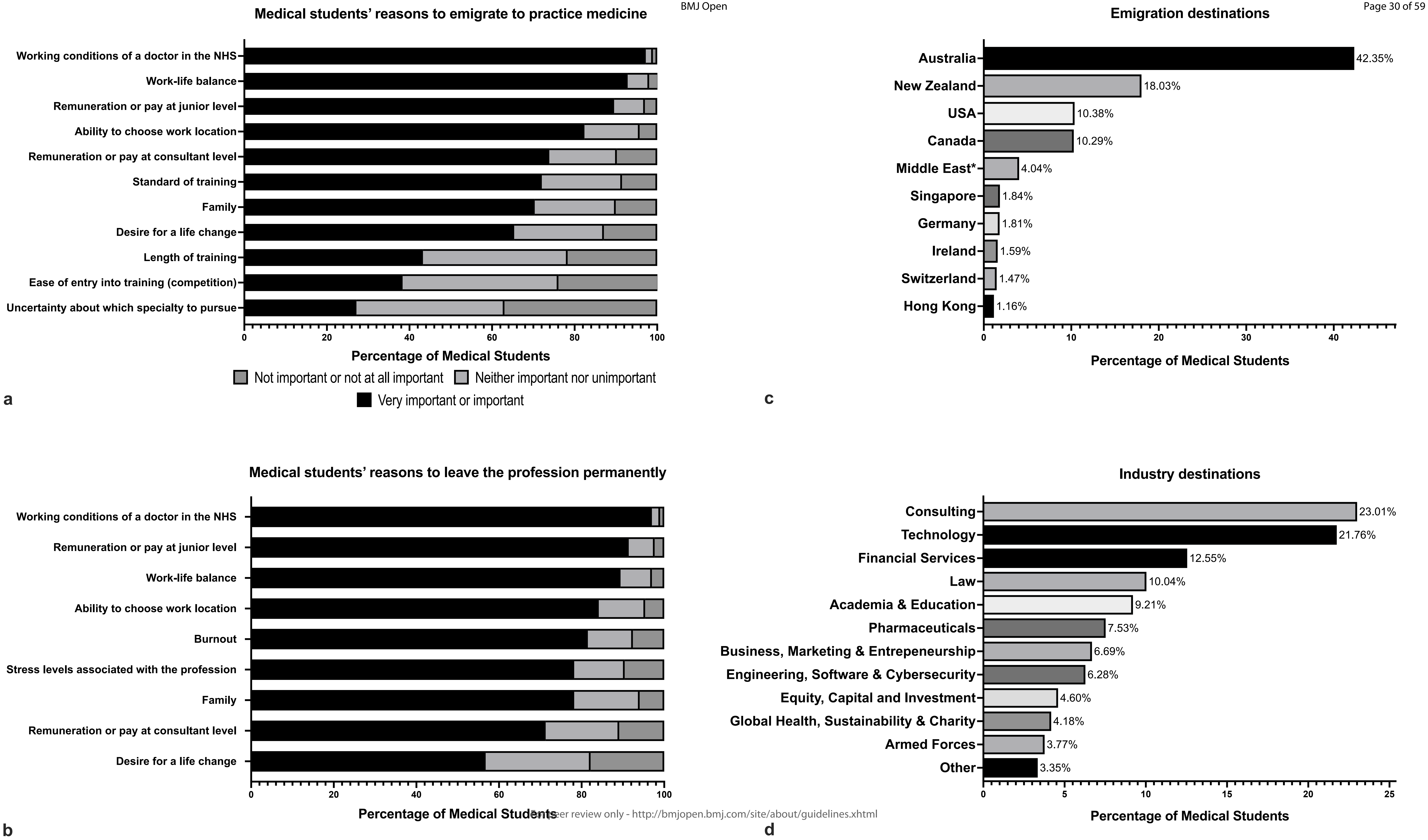
Medical students' reasons to emigrate to practice medicine
Working conditions of a doctor in the NHS
Work-life balance
Remuneration or pay at junior level
Ability to choose work location
Remuneration or pay at consultant level
Standard of training
Family
Desire for a life change
Length of training
Ease of entry into training (competition)
Uncertainty about which specialty to pursue
0
20
40
60
80
100
Percentage of Medical Students
Not important or not at all important
Neither important nor unimportant
Very important or important
a
Emigration destinations
Australia
42.35%
New Zealand
18.03%
USA
10.38%
Canada
10.29%
Middle East*
4.04%
Singapore
1.84%
Germany
1.81%
Ireland
1.59%
Switzerland
1.47%
Hong Kong
1.16%
0
10
20
30
40
Percentage of Medical Students
c
Medical students' reasons to leave the profession permanently
Working conditions of a doctor in the NHS
Remuneration or pay at junior level
Work-life balance
Ability to choose work location
Burnout
Stress levels associated with the profession
Family
Remuneration or pay at consultant level
Desire for a life change
0
20
40
60
80
100
Percentage of Medical Students
b
Industry destinations
Consulting
23.01%
Technology
21.76%
Financial Services
12.55%
Law
10.04%
Academia & Education
9.21%
Pharmaceuticals
7.53%
Business, Marketing & Entrepeneurship
6.69%
Engineering, Software & Cybersecurity
6.28%
Equity, Capital and Investment
4.60%
Global Health, Sustainability & Charity
4.18%
Armed Forces
3.77%
Other
3.35%
0
5
10
15
20
25
Percentage of Medical Students
d

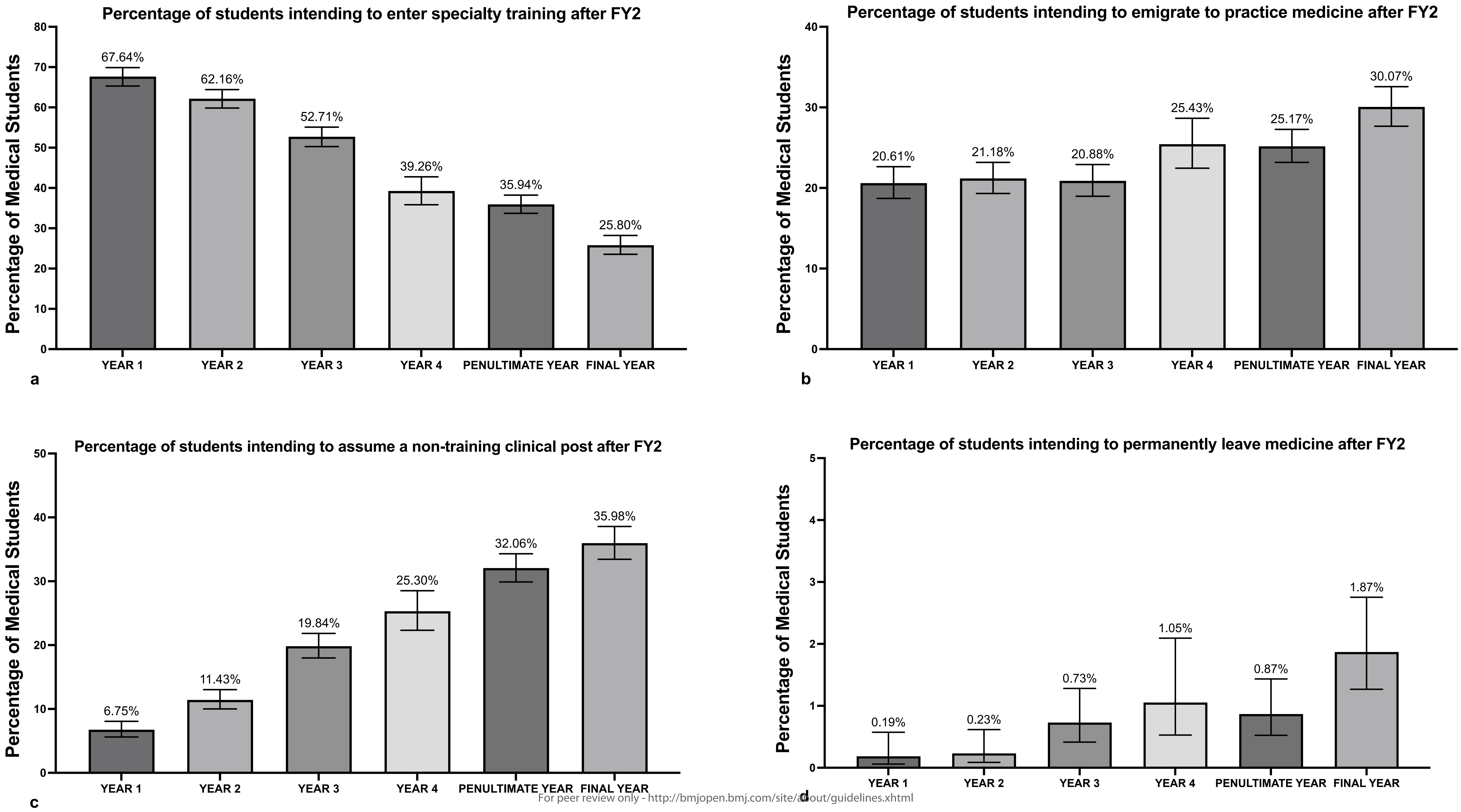
Percentage of students intending to enter specialty training after FY2
Percentage of Medical Students
67.64%
62.16%
52.71%
39.26%
35.94%
25.80%
YEAR 1
YEAR 2
YEAR 3
YEAR 4
PENULTIMATE YEAR
FINAL YEAR
a
Percentage of students intending to emigrate to practice medicine after FY2
Percentage of Medical Students
20.61%
21.18%
20.88%
25.43%
25.17%
30.07%
YEAR 1
YEAR 2
YEAR 3
YEAR 4
PENULTIMATE YEAR
FINAL YEAR
b
Percentage of students intending to assume a non-training clinical post after FY2
Percentage of Medical Students
6.75%
11.43%
19.84%
25.30%
32.06%
35.98%
YEAR 1
YEAR 2
YEAR 3
YEAR 4
PENULTIMATE YEAR
FINAL YEAR
c
Percentage of students intending to permanently leave medicine after FY2
Percentage of Medical Students
0.19%
0.23%
0.73%
1.05%
0.87%
1.87%
YEAR 1
YEAR 2
YEAR 3
YEAR 4
PENULTIMATE YEAR
FINAL YEAR
d

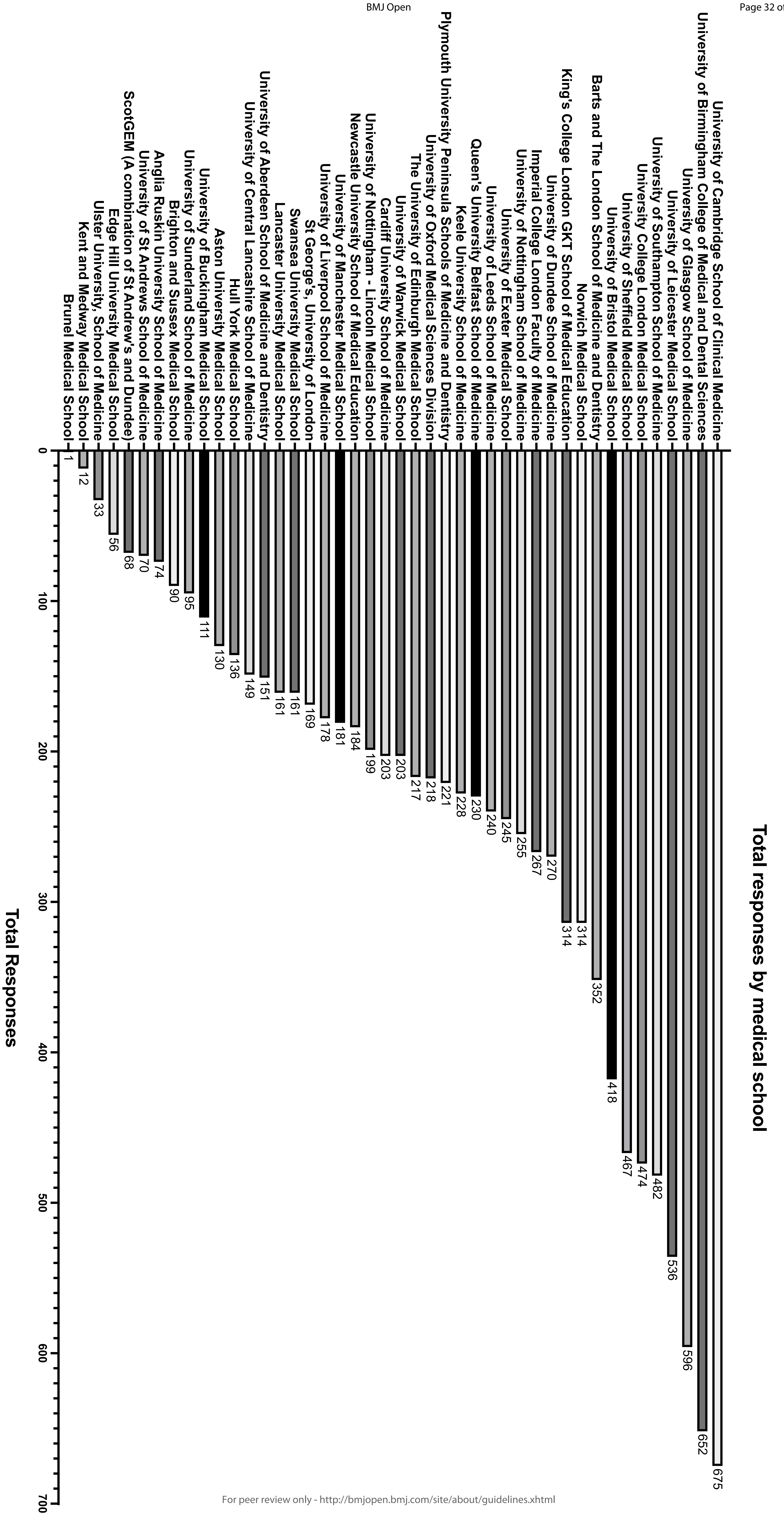
Total responses by medical school
University of Cambridge School of Clinical Medicine
675
University of Birmingham College of Medical and Dental Sciences
652
University of Glasgow School of Medicine
596
University of Leicester Medical School
536
University of Southampton School of Medicine
482
University College London Medical School
474
University of Sheffield Medical School
467
University of Bristol Medical School
418
Barts and The London School of Medicine and Dentistry
352
Norwich Medical School
314
King's College London GKT School of Medical Education
314
University of Dundee School of Medicine
270
Imperial College London Faculty of Medicine
267
University of Nottingham School of Medicine
255
University of Exeter Medical School
245
University of Leeds School of Medicine
240
Queen's University Belfast School of Medicine
230
Keele University School of Medicine
228
Plymouth University Peninsula Schools of Medicine and Dentistry
221
University of Oxford Medical Sciences Division
218
The University of Edinburgh Medical School
217
University of Warwick Medical School
203
Cardiff University School of Medicine
203
University of Nottingham - Lincoln Medical School
199
Newcastle University School of Medical Education
184
University of Manchester Medical School
181
University of Liverpool School of Medicine
178
St George's, University of London
169
Swansea University Medical School
161
Lancaster University Medical School
161
University of Aberdeen School of Medicine and Dentistry
151
University of Central Lancashire School of Medicine
149
Hull York Medical School
136
Aston University Medical School
130
University of Buckingham Medical School
111
University of Sunderland School of Medicine
95
Brighton and Sussex Medical School
90
Anglia Ruskin University School of Medicine
74
University of St Andrews School of Medicine
70
ScotGEM (A combination of St Andrew's and Dundee)
68
Edge Hill University Medical School
56
Ulster University, School of Medicine
33
Kent and Medway Medical School
12
Brunel Medical School
1
0
100
200
300
400
500
600
700
Total Responses

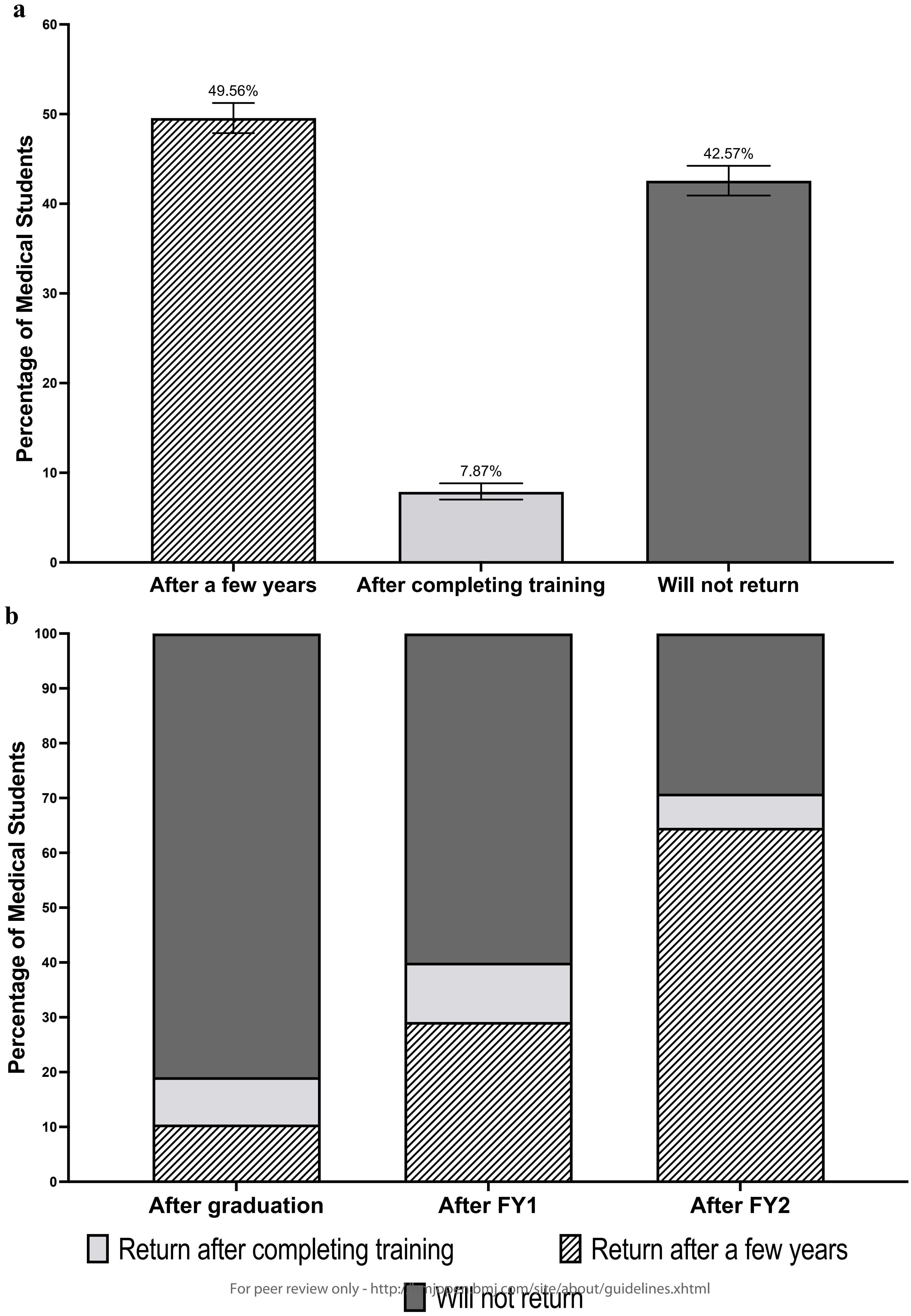
a
Percentage of Medical Students
60
50
40
30
20
10
0
49.56%
7.87%
42.57%
After a few years
After completing training
Will not return
b
Percentage of Medical Students
100
90
80
70
60
50
40
30
20
10
0
After graduation
After FY1
After FY2
Return after completing training
Return after a few years
Will not return

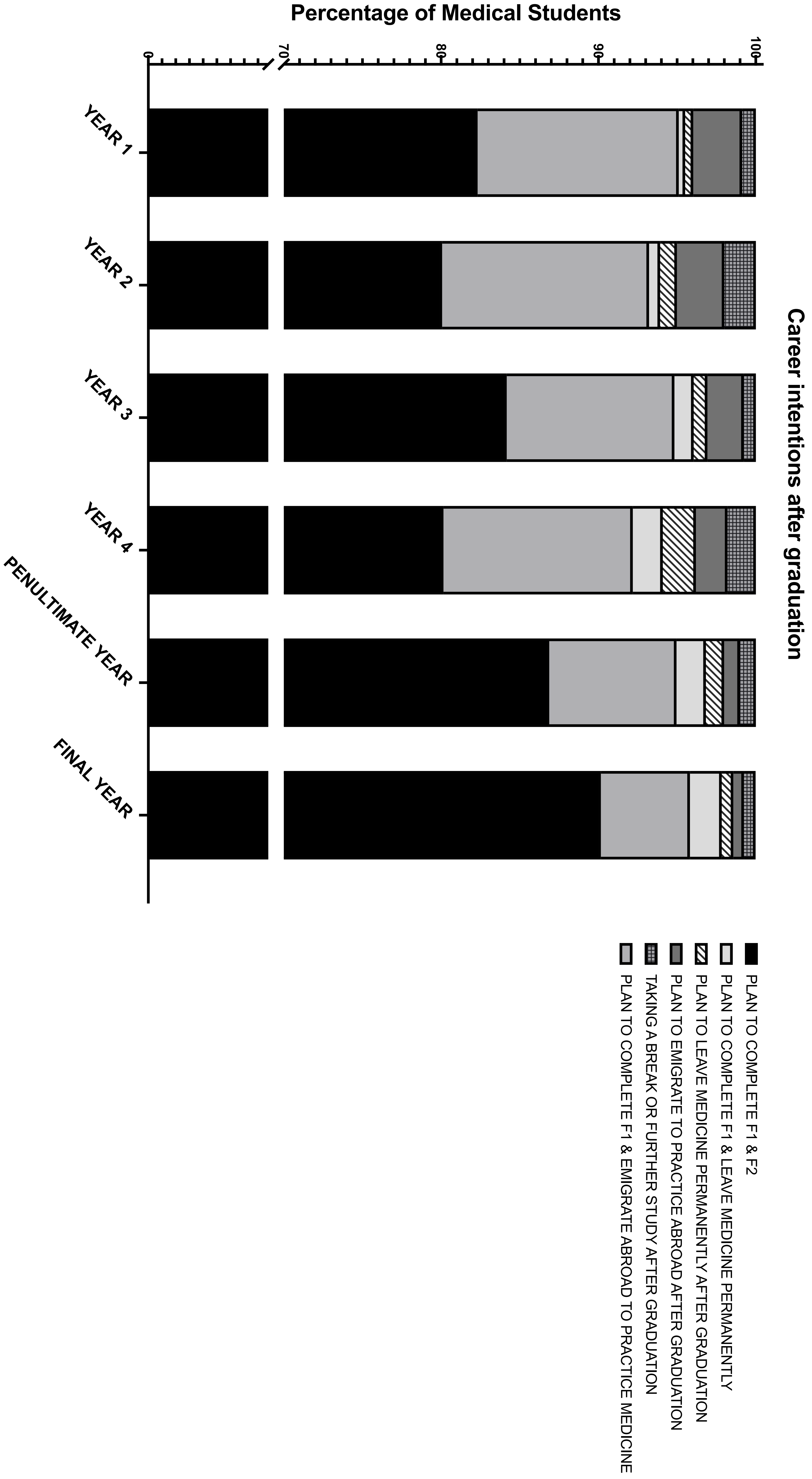
Percentage of Medical Students
0
70
80
90
100
YEAR 1
YEAR 2
YEAR 3
YEAR 4
PENULTIMATE YEAR
FINAL YEAR
Career intentions after graduation
PLAN TO COMPLETE F1 & F2
PLAN TO COMPLETE F1 & LEAVE MEDICINE PERMANENTLY
PLAN TO LEAVE MEDICINE PERMANENTLY AFTER GRADUATION
PLAN TO EMIGRATE TO PRACTICE ABROAD AFTER GRADUATION
TAKING A BREAK OR FURTHER STUDY AFTER GRADUATION
PLAN TO COMPLETE F1 & EMIGRATE ABROAD TO PRACTICE MEDICINE

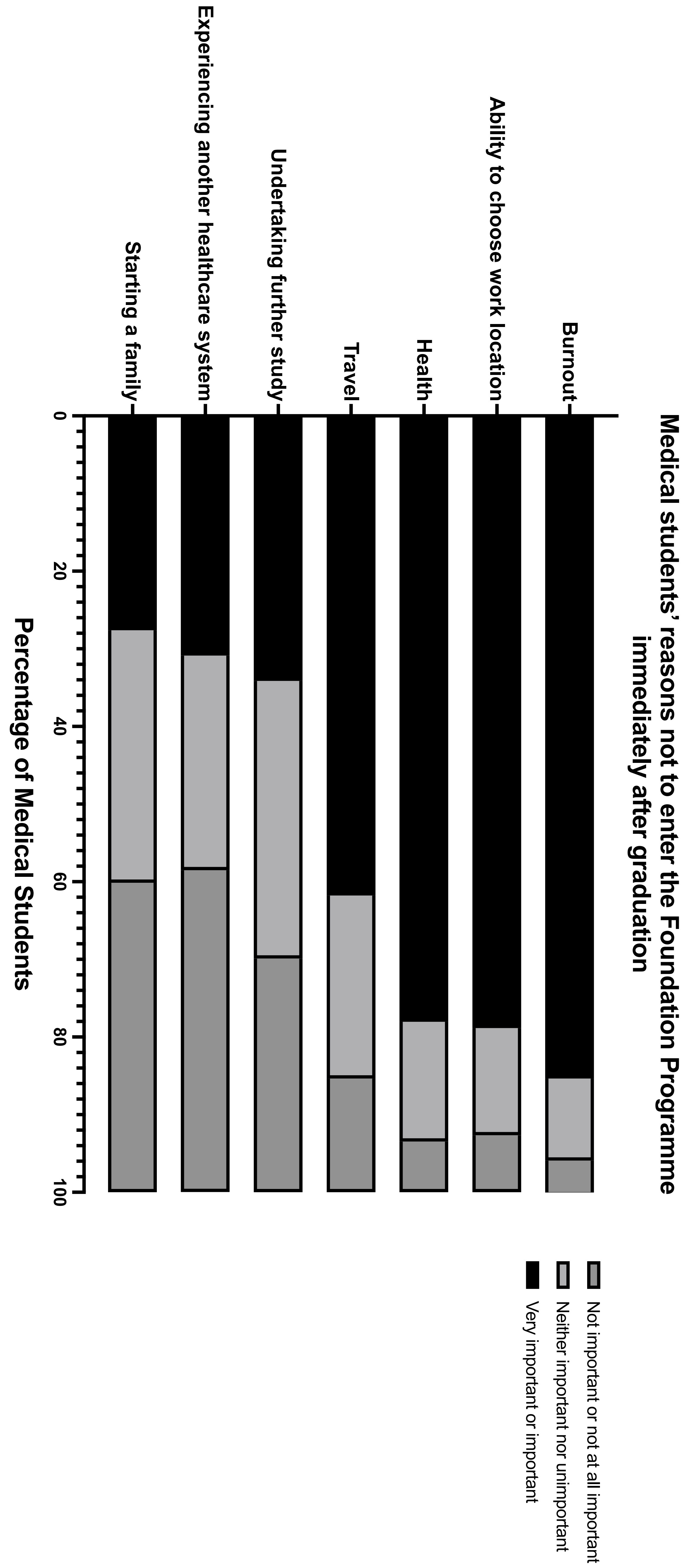
Medical students' reasons not to enter the Foundation Programme immediately after graduation
Starting a family
Experiencing another healthcare system
Undertaking further study
Travel
Health
Ability to choose work location
Burnout
0
20
40
60
80
100
Percentage of Medical Students
Not important or not at all important
Neither important nor unimportant
Very important or important

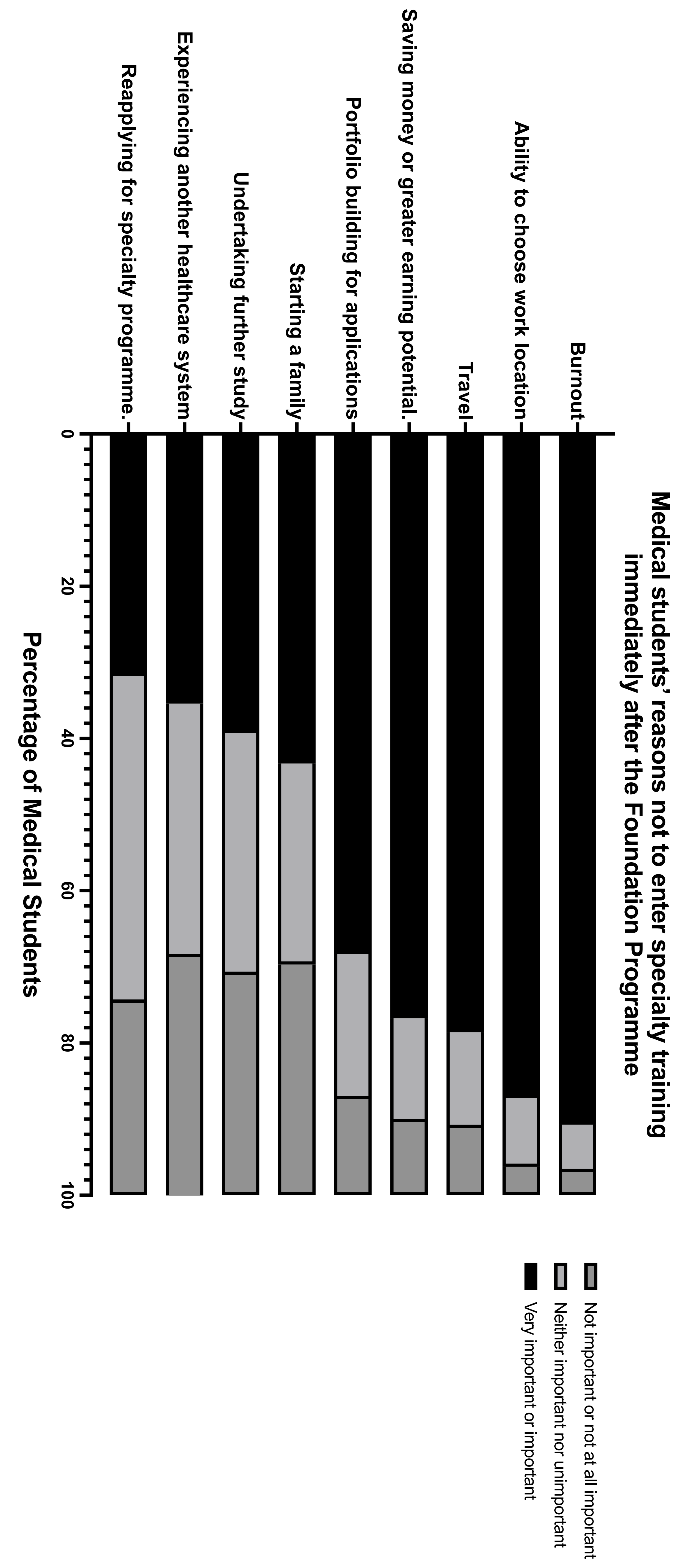
Medical students' reasons not to enter specialty training immediately after the Foundation Programme
Burnout
Ability to choose work location
Travel
Saving money or greater earning potential.
Portfolio building for applications
Starting a family
Undertaking further study
Experiencing another healthcare system
Reapplying for specialty programme.
0
20
40
60
80
100
Percentage of Medical Students
Not important or not at all important
Neither important nor unimportant
Very important or important

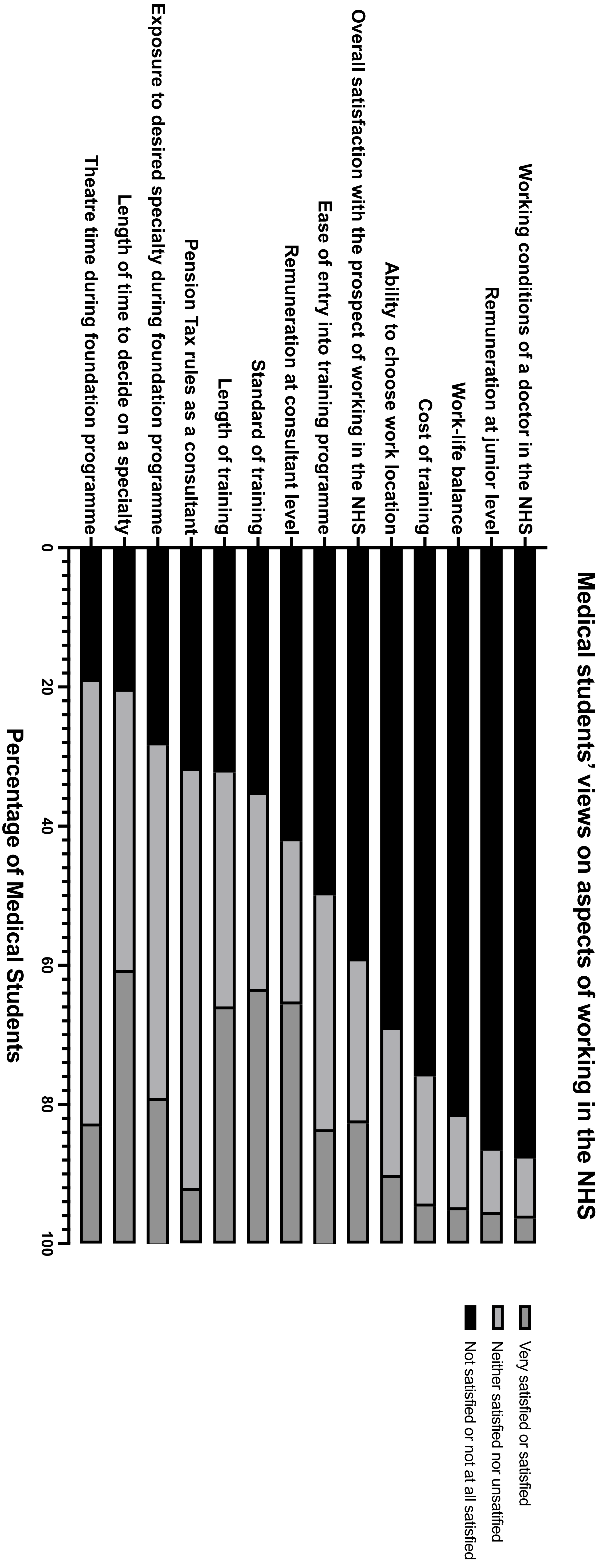
Medical students' views on aspects of working in the NHS
Working conditions of a doctor in the NHS
Remuneration at junior level
Work-life balance
Cost of training
Ability to choose work location
Overall satisfaction with the prospect of working in the NHS
Ease of entry into training programme
Remuneration at consultant level
Standard of training
Length of training
Pension Tax rules as a consultant
Exposure to desired specialty during foundation programme
Length of time to decide on a specialty
Theatre time during foundation programme
0
20
40
60
80
100
Percentage of Medical Students
Very satisfied or satisfied
Neither satisfied nor unsatified
Not satisfied or not at all satisfied

Students' intention after graduation	Number (%)	Confidence Interval
Complete both FY1 and FY2	8,806 (83.98)	[83.26, 84.67]
Complete FY1 and emigrate to practice medicine	1,101 (10.50)	[9.93,11.10]
Complete FY1 and leave medicine permanently	132 (1.26)	[1.06, 1.49]
Leave medicine permanently	104 (0.99)	[0.82, 1.20]
Emigrate to practice medicine	220 (2.10)	[1.84, 2.39]
Take a break or undertake further study	123 (1.17)	[0.98, 1.40]

view only

Students' intention after the Foundation Programme	Number (%)	Confidence Interval
Enter specialty training in the UK	4,294 (48.76)	[47.72, 49.81]
Assume a non-training clinical job in the UK	1,859 (21.11)	[20.27, 21.98]
Emigrate to practice medicine abroad (including temporarily)	2,071 (23.52)	[22.64, 24.42]
Take a break or undertake further study	515 (5.85)	[5.38, 6.36]
Leave medicine permanently	67 (0.76)	[0.60, 0.97]

review only

Students' intention after graduation	Year 1	Year 2	Year 3	Year 4 (not penultimate year)	Penultimate Year	Final Year
Complete both FY1 and FY2	1616 (82.32)	1723 (80.07)	1643 (84.17)	759 (80.15)	1728 (86.88)	1337 (90.16)
Complete FY1 and emigrate to practice medicine	251 (12.79)	283 (13.15)	208 (10.66)	114 (12.04)	161 (8.09)	84 (5.66)
Complete FY1 and leave medicine permanently	8 (0.41)	15 (0.70)	24 (1.23)	18 (1.90)	37 (1.86)	30 (2.02)
Leave medicine permanently	10 (0.51)	23 (1.07)	17 (0.87)	20 (2.11)	23 (1.16)	11 (0.74)
Emigrate to practice medicine	61 (3.11)	65 (3.02)	45 (2.31)	19 (2.01)	20 (1.01)	10 (0.67)
Take a break or undertake further study	17 (0.87)	43 (2.00)	15 (0.77)	17 (1.80)	20 (1.01)	11 (0.74)

Students' intention after the Foundation Programme	Year 1	Year 2	Year 3	Year 4 (not penultimate year)	Penultimate Year	Final Year
Enter specialty training in the UK	1093 (67.64)	1071 (62.16)	866 (52.71)	298 (39.26)	621 (35.94)	345 (25.80)
Assume a non-training clinical job in the UK	109 (6.75)	197 (11.43)	326 (19.84)	192 (25.30)	554 (32.06)	481 (35.98)
Emigrate to practice medicine abroad (including temporarily)	333 (20.61)	365 (21.18)	343 (20.88)	193 (25.43)	435 (25.17)	402 (30.07)
Take a break or undertake further study	78 (4.83)	86 (4.99)	96 (5.84)	68 (8.96)	103 (5.96)	84 (6.28)
Leave medicine permanently	3 (0.19)	4 (0.23)	12 (0.73)	8 (1.05)	15 (0.87)	25 (1.87)

For peer review only - http://bmjopen.bmj.com/site/about/guidelines.xhtml

Demographic subgroup	Return prospects		
	After a few years	After completing training	Will not return
Ethnicity			
White	1,133 (58.46)	131 (6.76)	674 (34.78)
Asian or Asian British	334 (36.66)	78 (8.56)	499 (54.77)
Black, Black British, Caribbean or African	79 (44.89)	15 (8.52)	82 (46.59)
Mixed or multiple ethnic groups	88 (46.07)	23 (12.04)	80 (41.88)
Other	40 (28.37)	18 (12.77)	83 (58.87)
Prefer not to say	7 (20.00)	2 (5.71)	26 (74.29)
Gender			
Female	1,165 (53.37)	175 (8.02)	843 (38.62)
Male	512 (42.99)	92 (7.72)	587 (49.29)
Non-binary	3 (25.00)	0 (0)	9 (75.00)
Prefer not to say	1 (16.67)	0 (0)	5 (83.33)
Level of education			
Postgraduate	311 (46.49)	51 (7.62)	307 (45.89)

Undergraduate	1,370 (50.31)	216 (7.93)	1137 (41.76)
Previous schooling			
Private education	578 (44.91)	113 (8.78)	596 (46.31)
State education	1,072 (52.96)	143 (7.07)	809 (39.97)
Prefer not to say	31 (38.27)	11 (13.58)	39 (48.15)
Fee status			
Home	1,572 (56.67)	221 (7.97)	981 (35.36)
EU	45 (20.74)	17 (7.83)	155 (71.43)
International (Non-EU)	64 (15.96)	29 (7.23)	308 (76.81)
Current year of study			
Year 1	297 (46.05)	65 (10.08)	283 (43.88)
Year 2	346 (48.53)	55 (7.71)	312 (43.76)
Year 3	281 (47.15)	55 (9.23)	260 (43.62)
Year 4 (not penultimate year)	163 (50.00)	25 (7.67)	138 (42.33)
Penultimate year	313 (50.81)	47 (7.63)	256 (41.56)
Final year	281 (56.65)	20 (4.03)	195 (39.31)

Total	1681 (49.56)	267 (7.87)	1444 (42.57)

For peer review only

AIMS UNIVERSITY OF CAMBRIDGE School of Clinical Medicine

Participant Information Sheet

Ascertaining the career Intentions of Medical Students in the UK post-graduation: a cross-sectional study

Thank you for your interest in participating in this study. Please take a moment to read the following information. If you have any questions or concerns, please contact the principal researcher, Tomas Ferreira at tf385@cam.ac.uk.

What is the aim of this study? This study aims to determine current medical students' career intentions post-graduation and post-foundation training, to identify factors involved in decision making for students' career choices and to analyse medical students' views on how the prospect of working in the NHS could be improved.

Why have I been selected to take part? All medical students currently studying at UK medical schools recognised by the General Medical Council (GMC) are being invited to take part in the questionnaire.

What do I have to do? If you decide to participate in this study, you will be asked to complete a questionnaire about your background, your career intentions after graduation and after foundation training, and your motivations for these answers. This study is voluntary. If you choose to participate, you will be asked to complete the survey by clicking on the link found at the end of this document. This survey is expected to take about 4-7 minutes to complete, but there is no time limit. No background knowledge is required. By submitting the survey, you consent to the collection and storage of data in accordance with the UK General Data Protection Regulation (GDPR) within the survey. For more information on GDPR please click on the following link: https://gdpr-info.eu.

Do I have to participate? Participation is entirely voluntary. You may withdraw at any point during the questionnaire for any reason, before submitting your answers, by closing the browser. In cases of withdrawal from the study prior to submission of the survey, no data is recorded. If you have already submitted data and wish to withdraw from the study, please contact tf385@cam.ac.uk by 31st March 2023.

Who has approved this study? This study has been reviewed and approved by the University of Cambridge's Research Ethics Committee on the 5th of January 2023, reference PRE.2022.124.

How will my data be used? All answers will be anonymous, and we will take all reasonable precautions to ensure that they remain confidential. Data will be stored in a password-protected file and may be used in academic publications. Your IP address will not be stored. After completion of data collection, no email addresses will be stored unless you consent to being followed up via the survey's final question. Prior to completion of data collection, we will store your institutional email address to confirm your student status. Research data will be stored for a minimum of ten years after publication or public release.

Who will have access to my data? Qualtrics is the data controller of the personal data held about you and, as such, will determine how your personal data are used. Their privacy notice can be found here: https://www.qualtrics.com/privacy-statement. Qualtrics will share any email address you provide and your anonymised responses with the University of Cambridge, for the purposes of research as highlighted above. Researchers involved in the project will have access to this anonymised data.

Are there any benefits to taking part? Although there are no immediate individual benefits to participating in this survey, you are given the opportunity to contribute to research which may impact you. You may find this survey an opportunity to self-reflect on your career plans after you graduate. Additionally, all participants will be entered into a prize draw for a chance to win £300!

Will the research be published? The findings of this study may be published in peer-reviewed journals, presented at conferences and a summary of the findings will be made available on social media.

For peer review only

AIMS UNIVERSITY OF CAMBRIDGE School of Clinical Medicine

Are there any possible risks involved with my participation? There are no anticipated disadvantages, side effects, risks, and/or discomforts of taking part in this study. If participating in the study leads to distress, you may stop the survey at any time. If your distress continues after leaving the survey, we have provided a list of supportive services nationwide that can be helpful and that you might consider contacting (appears at the close of survey).

Who do I contact if I have a concern about the study or I wish to complain? If you have a concern about any aspect of this project, please speak to the principal researcher tf385@cam.ac.uk. If you remain unhappy or wish to make a formal complaint, please contact the Research and Information Governance, School of Clinical Medicine, University of Cambridge: Research.Governance@medschl.cam.ac.uk.

How do I find out what was learned in this study? This study is expected to be completed by April 2023. If you would like a brief summary of the results, please write to us by email to request information

Who to contact for further details? For any further questions or more information on the study, please contact us on the following email address: tf385@cam.ac.uk.

Link to the survey: https://cambridge.eu.qualtrics.com/jfe/form/SV_cx55RTspDLTIzWK

Kind Regards,

Tomas Ferreira
AIMS Study Lead

Dr. Rita Horvath
Supervisor, Director of Research, Horvath Laboratory, Department of Clinical Neurosciences, University of Cambridge

For peer review only

Demographics

AIMS - Ascertaining the career Intentions of UK Medical Students' post-graduation: a cross-sectional survey

Thank you for taking part in the study. Please note that participating in this survey is entirely optional.

In 2010, 83.1% of Foundation Year 2 (F2) doctors went into further training. In 2019, this number was only 34.9%. This represents a significant change in the makeup of doctors in the UK on a backdrop of a wider NHS staffing challenge. AIMS endeavours to understand the factors involved in medical students' decision-making around their future career. Specifically, we are interested in what students' current career plans are, and why they may, or may not choose to pursue specialty training, or a medical career more broadly, in the UK. We are also hoping to understand current views on the prospect of working in the NHS.

All responses will remain confidential. Your email address will only be visible to the study leads and will be deleted from our records once all data has been collected (unless you consent to being followed up at the end of the survey) and there is no need for further communication. You may withdraw from the study at any point by contacting Tomas Ferreira, tf385@cam.ac.uk.

By submitting your answers to the survey, you consent to us collecting this data and acknowledging that anonymised data may be published and used for purposes beyond this study. Ethical approval was granted by the University of Cambridge Research Ethics Committee (PRE.2022.124) on 5 January 2023.

All participants will be entered into a prize draw for the chance to win £300!

I understand that my participation is voluntary and that I am free to withdraw at any time without giving a reason and I consent to participate in this study.

○ Yes

Email Address

Please enter your institutional email address (ending in 'ac.uk'. We will use this to verify your student status and we may contact you to notify you of a prize win or for clarification of responses). Please ensure there are no spaces at the end of your email.

Age

Gender

Which of the below options best describes your ethnicity?

University

Year of study - Please read description.

(as of September 2022)

- If you are in your fourth year of study and it is your final year, please select final year (i.e., GEM)

- If you are in your fourth year of study but it is your penultimate year, please select penultimate year.

- If you are currently intercalating, please select your current year of study (e.g., intercalating between 3rd and 4th year on a 5 year course please select Year 4).

- Treat the first year of a GEM course as still equivalent to first year.

- If you are in a "Foundation" or "Gateway" year (also known as Y0), please select Year 1.

- ◯ Year 1
- ◯ Year 2
- ◯ Year 3
- ◯ Year 4 (not penultimate year)
- ◯ Penultimate year
- ◯ Final year

What is your expected graduation year?

Do you have a previous or intercalated degree?

- ◯ Yes, prior to studying Medicine.
- ◯ Yes, an intercalated degree.
- ◯ Yes, both.
- ◯ Not yet, but intend on intercalating.

- Not yet, but currently intercalating.
- No.

What is your student fee status?

- Home
- EU
- International (Non-EU)

Did you, at any point in your education, attend a fee-paying independent school?

E.g., private school.

- Yes
- No
- Prefer not to say

Intentions

Do you intend to join the NHS Foundation Programme after graduation?

- Yes - plan to complete F1 & F2
- Yes - plan to complete F1 & emigrate to practice abroad
- Yes - plan to complete F1 & leave medicine permanently.
- No - plan on leaving medicine permanently.
- No - plan on emigrating
- No - plan on taking a break or undertaking further study.

What do you intend to do after completing the NHS Foundation Programme?

- Enter specialty training in the UK
- Non-training clinical job in the UK, e.g. 'F3 year', JCF or CTF
- Emigrating to practice medicine abroad (including temporarily)
- Taking a break or undertaking further study

○ Leaving medicine permanently

You have indicated your intention to leave medicine permanently. In which industry do you plan to work after leaving medicine? If unsure, please enter N/A"

In which country do you intend to practice?

If you are unsure, please enter N/A.

Reasons for emigrating to practice abroad

In your previous answers, you have indicated your intentions to practice medicine abroad.

Please indicate the level of importance of the below factors in your decision making

	Very important	Important	Neither important nor unimportant	Not important	Not at all important
Remuneration or pay at junior level	○	○	○	○	○
Remuneration or pay at consultant level	○	○	○	○	○
Work-life balance	○	○	○	○	○
Family	○	○	○	○	○
Desire for a life change	○	○	○	○	○
Ease of entry into training (competition)	○	○	○	○	○
Length of training	○	○	○	○	○
Standard of training	○	○	○	○	○
Ability to choose work location	○	○	○	○	○

Working conditions of a doctor in the NHS	○	○	○	○	○
Uncertainty about which specialty to pursue	○	○	○	○	○

You have indicated that you intend to emigrate to practice medicine, do you intend on returning to the UK?

- ○ Yes - after a few years
- ○ Yes - after I complete my training
- ○ No

Reasons for leaving medicine permanently

In your previous answers, you have indicated your intentions to leave medicine permanently.

Please indicate the level of importance of the below factors in your decision making.

	Very important	Important	Neither important nor unimportant	Not important	Not at all important
Remuneration or pay at junior level	○	○	○	○	○
Remuneration or pay at consultant level	○	○	○	○	○
Work-life balance	○	○	○	○	○
Family	○	○	○	○	○
Desire for a life change	○	○	○	○	○
Ability to choose work location	○	○	○	○	○
Working conditions of a doctor in the NHS	○	○	○	○	○
Stress levels associated with profession	○	○	○	○	○
Burnout	○	○	○	○	○

Reasons for not entering specialty training immediately after F2

In your previous answers, you have indicated your intentions to not enter specialty training immediately after completing your F2 year. Please indicate the level of importance of the below factors in your decision making.

	Very important	Important	Neither important nor unimportant	Not important	Not at all important
Saving money or greater earning potential.	○	○	○	○	○
Undertaking further study	○	○	○	○	○
Portfolio building for applications	○	○	○	○	○
Travel	○	○	○	○	○
Experiencing another healthcare system	○	○	○	○	○
Starting a family	○	○	○	○	○
Reapplying for specialty programme.	○	○	○	○	○
Ability to choose work location	○	○	○	○	○
Burnout	○	○	○	○	○
Uncertainty about which specialty to pursue	○	○	○	○	○

Reasons for not entering foundation training immediately after graduation

In your previous answers, you have indicated your intentions to not enter foundation training immediately after graduation. Please indicate the level of importance of the below factors in your decision making.

	Very important	Important	Neither important nor unimportant	Not important	Not at all important

Undertaking further study	○	○	○	○	○
Travel	○	○	○	○	○
Experiencing another healthcare system	○	○	○	○	○
Starting a family	○	○	○	○	○
Ability to choose work location	○	○	○	○	○
Health	○	○	○	○	○
Burnout	○	○	○	○	○

Views on a career in the NHS

For each of the points below, how would you describe your level of satisfaction regarding their current status in the NHS?

	Very satisfied	Satisfied	Neither satisfied nor unsatisfied	Not satisfied	Not at all satisfied
Remuneration or pay at junior level	○	○	○	○	○
Remuneration or pay at consultant level	○	○	○	○	○
Work-life balance	○	○	○	○	○
Ability to choose work location	○	○	○	○	○
Ease of entry into training (competition)	○	○	○	○	○
Length of training	○	○	○	○	○
Standard of training	○	○	○	○	○
Working conditions of a doctor in the NHS	○	○	○	○	○
Exposure to desired specialty during foundation programme	○	○	○	○	○
Theatre time during foundation programme	○	○	○	○	○

Cost of training (i.e., mandatory exams, courses, memberships)	◯	◯	◯	◯	◯
Length of time to decide on a specialty	◯	◯	◯	◯	◯
Pension Tax rules as a consultant	◯	◯	◯	◯	◯
Overall satisfaction with the prospect of working in the NHS	◯	◯	◯	◯	◯

Are you certain about which specialty you wish to pursue?

◯ Very certain
◯ Somewhat certain
◯ Neither certain nor uncertain
◯ Somewhat uncertain
◯ Very uncertain

Which specialty (or specialties) most interest you?

Select **up to** a maximum of 3 options (if you are certain, please select only one)

☐ Acute internal medicine
☐ Allergy
☐ Anaesthetics
☐ Audio vestibular medicine
☐ Cardio-thoracic surgery
☐ Cardiology
☐ Clinical genetics
☐ Clinical neurophysiology
☐ Clinical oncology
☐ Community sexual and reproductive health
☐ Dermatology
☐ Emergency medicine
☐ Endocrinology and diabetes mellitus
☐ Gastro-enterology
☐ General practice
☐ General surgery

- ☐ Genito-urinary medicine
- ☐ Geriatric medicine
- ☐ Haematology
- ☐ Histopathology
- ☐ Immunology
- ☐ Infectious diseases
- ☐ Intensive care medicine
- ☐ Medical microbiology
- ☐ Medical oncology
- ☐ Neurology
- ☐ Neurosurgery
- ☐ Nuclear medicine
- ☐ Obstetrics and gynaecology
- ☐ Occupational medicine
- ☐ Ophthalmology
- ☐ Oral and maxillo-facial surgery
- ☐ Otolaryngology (ENT)
- ☐ Paediatric surgery
- ☐ Paediatrics
- ☐ Palliative medicine
- ☐ Pathology
- ☐ Plastic surgery
- ☐ Psychiatry
- ☐ Public health medicine
- ☐ Radiology
- ☐ Rehabilitation medicine
- ☐ Renal medicine
- ☐ Respiratory medicine
- ☐ Rheumatology
- ☐ Sport and exercise medicine
- ☐ Trauma and orthopaedic surgery
- ☐ Tropical medicine
- ☐ Urology
- ☐ Vascular surgery

What steps could be taken to improve the prospect of working in the NHS?

(Optional)

Do you consent to being contacted by us for potential follow-up studies regarding your career intentions?

We will store your email address to contact you in the future.

- ◯ Yes
- ◯ No

Powered by Qualtrics

For peer review only

Eligible Medical Schools and Approved Programmes

A combination of the universities of Dundee and St. Andrews (ScotGEM)
A combination of the University of Brighton and the University of Sussex
A combination of the University of Hull and the University of York
Anglia Ruskin School of Medicine
Aston Medical School
Brunel University London Medical School
Cardiff University
Edge Hill University Medical School
Imperial College London
Keele University
Kent and Medway Medical School
King's College London
Lancaster University
Queen Mary University of London
St George's University of London
Swansea University
The Queen's University of Belfast
The University of Aberdeen
The University of Birmingham
The University of Bristol
The University of Buckingham
The University of Cambridge
The University of Central Lancashire
The University of Dundee
The University of Dundee
The University of East Anglia
The University of Edinburgh
The University of Exeter
The University of Glasgow
The University of Leeds
The University of Leicester
The University of Liverpool
The University of Manchester
The University of Newcastle
The University of Nottingham
The University of Oxford
The University of Plymouth
The University of Sheffield
The University of Southampton
The University of St Andrew's
The University of Warwick
Ulster University School of Medicine
University College London
University of Sunderland School of Medicine

Excluded for lack of cohort at time of recruitment:

- University of Chester Medical School
- Three Counties Medical School

For peer review only

STROBE Statement—Checklist of items that should be included in reports of ***cross-sectional studies***

	Item No	Recommendation
**Title and abstract**	1	(*a*) Indicate the study's design with a commonly used term in the title or the abstract
		(*b*) Provide in the abstract an informative and balanced summary of what was done and what was found
**Introduction**		
Background/rationale	2	Explain the scientific background and rationale for the investigation being reported
Objectives	3	State specific objectives, including any prespecified hypotheses
**Methods**		
Study design	4	Present key elements of study design early in the paper
Setting	5	Describe the setting, locations, and relevant dates, including periods of recruitment, exposure, follow-up, and data collection
Participants	6	(*a*) Give the eligibility criteria, and the sources and methods of selection of participants
Variables	7	Clearly define all outcomes, exposures, predictors, potential confounders, and effect modifiers. Give diagnostic criteria, if applicable
Data sources/ measurement	8*	For each variable of interest, give sources of data and details of methods of assessment (measurement). Describe comparability of assessment methods if there is more than one group
Bias	9	Describe any efforts to address potential sources of bias
Study size	10	Explain how the study size was arrived at
Quantitative variables	11	Explain how quantitative variables were handled in the analyses. If applicable, describe which groupings were chosen and why
Statistical methods	12	(*a*) Describe all statistical methods, including those used to control for confounding
		(*b*) Describe any methods used to examine subgroups and interactions
		(*c*) Explain how missing data were addressed
		(*d*) If applicable, describe analytical methods taking account of sampling strategy
		(*e*) Describe any sensitivity analyses
**Results**		
Participants	13*	(a) Report numbers of individuals at each stage of study—eg numbers potentially eligible, examined for eligibility, confirmed eligible, included in the study, completing follow-up, and analysed
		(b) Give reasons for non-participation at each stage
		(c) Consider use of a flow diagram
Descriptive data	14*	(a) Give characteristics of study participants (eg demographic, clinical, social) and information on exposures and potential confounders
		(b) Indicate number of participants with missing data for each variable of interest
Outcome data	15*	Report numbers of outcome events or summary measures
Main results	16	(*a*) Give unadjusted estimates and, if applicable, confounder-adjusted estimates and their precision (eg, 95% confidence interval). Make clear which confounders were adjusted for and why they were included
		(*b*) Report category boundaries when continuous variables were categorized
		(*c*) If relevant, consider translating estimates of relative risk into absolute risk for a meaningful time period
Other analyses	17	Report other analyses done—eg analyses of subgroups and interactions, and sensitivity analyses

For peer review only

**Discussion**		
Key results	18	Summarise key results with reference to study objectives
Limitations	19	Discuss limitations of the study, taking into account sources of potential bias or imprecision. Discuss both direction and magnitude of any potential bias
Interpretation	20	Give a cautious overall interpretation of results considering objectives, limitations, multiplicity of analyses, results from similar studies, and other relevant evidence
Generalisability	21	Discuss the generalisability (external validity) of the study results
**Other information**		
Funding	22	Give the source of funding and the role of the funders for the present study and, if applicable, for the original study on which the present article is based

*Give information separately for exposed and unexposed groups.

Note: An Explanation and Elaboration article discusses each checklist item and gives methodological background and published examples of transparent reporting. The STROBE checklist is best used in conjunction with this article (freely available on the Web sites of PLoS Medicine at http://www.plosmedicine.org/, Annals of Internal Medicine at http://www.annals.org/, and Epidemiology at http://www.epidem.com/). Information on the STROBE Initiative is available at www.strobe-statement.org.

1. a) “Career intentions of medical students in the United Kingdom: a national, cross-sectional study (AIMS Study)”
1. b) This has been done (Page 2).
2. The scientific background and rationale for the investigation can be found in page 3.
3. This can be found in the last paragraph of the introduction section in page 3.
4. Page 3 and Page 4
5. Page 4
6. Page 4
7. Page 3 and 4
8. Page 4
9. Page 4
10. Protocol “A sample size calculation was performed, and it was determined that a minimum of 8,026 participants are needed to have a confidence level of 95% that the results of the survey are within 1% representation of the total medical student population. This calculation used a population size for UK medical students acquired via a Freedom of Information request to the GMC.” (https://www.researchprotocols.org/2023/1/e45992). In reality, 10,486 responses received.
11. Page 4
12. Page 4
13. Pages 5-12. All questions were mandatory other than qualitative thematic analysis for which response proportion was given (page 12)
14. Pages 5 and 6. No missing data.
15. Pages 6-12.
16. Pages 6-12.
17. Pages 9-12.
18. Pages 13-15.
19. Page 2 and Page 15.
20. Pages 13-15.
21. Pages 13-15.
22. Page 17.

For peer review only - http://bmjopen.bmj.com/site/about/guidelines.xhtml

BMJ Open

Career intentions of medical students in the United Kingdom: a national, cross-sectional study (AIMS Study)

Journal:	*BMJ Open*
Manuscript ID	bmjopen-2023-075598.R2
Article Type:	Original research
Date Submitted by the Author:	27-Jul-2023
Complete List of Authors:	Ferreira, Tomas; University of Cambridge School of Clinical Medicine<br>Collins, Alexander; Imperial College London, School of Public Health, Faculty of Medicine<br>Feng, Oliver; University of Cambridge, Statistical Laboratory, Centre for Mathematical Sciences<br>Samworth, Richard; University of Cambridge, Statistical Laboratory<br>Horvath, Rita; University of Cambridge School of Clinical Medicine<br>., the AIMS Collaborative; University of Cambridge School of Clinical Medicine
**Primary Subject Heading**:	Medical education and training
Secondary Subject Heading:	Medical management, Health economics, Health informatics, Health policy, Health services research
Keywords:	Health policy < HEALTH SERVICES ADMINISTRATION & MANAGEMENT, QUALITATIVE RESEARCH, Health Education, Organisation of health services < HEALTH SERVICES ADMINISTRATION & MANAGEMENT, MEDICAL EDUCATION & TRAINING, Health economics < HEALTH SERVICES ADMINISTRATION & MANAGEMENT

SCHOLARONE™
Manuscripts

I, the Submitting Author has the right to grant and does grant on behalf of all authors of the Work (as defined in the below author licence), an exclusive licence and/or a non-exclusive licence for contributions from authors who are: i) UK Crown employees; ii) where BMJ has agreed a CC-BY licence shall apply, and/or iii) in accordance with the terms applicable for US Federal Government officers or employees acting as part of their official duties; on a worldwide, perpetual, irrevocable, royalty-free basis to BMJ Publishing Group Ltd ("BMJ") its licensees and where the relevant Journal is co-owned by BMJ to the co-owners of the Journal, to publish the Work in this journal and any other BMJ products and to exploit all rights, as set out in our licence.

The Submitting Author accepts and understands that any supply made under these terms is made by BMJ to the Submitting Author unless you are acting as an employee on behalf of your employer or a postgraduate student of an affiliated institution which is paying any applicable article publishing charge ("APC") for Open Access articles. Where the Submitting Author wishes to make the Work available on an Open Access basis (and intends to pay the relevant APC), the terms of reuse of such Open Access shall be governed by a Creative Commons licence – details of these licences and which Creative Commons licence will apply to this Work are set out in our licence referred to above.

Other than as permitted in any relevant BMJ Author's Self Archiving Policies, I confirm this Work has not been accepted for publication elsewhere, is not being considered for publication elsewhere and does not duplicate material already published. I confirm all authors consent to publication of this Work and authorise the granting of this licence.

For peer review only

Career intentions of medical students in the United Kingdom: a national, cross-sectional study (AIMS Study)

Tomas Ferreira[1], Alexander M. Collins[2], Oliver Feng[3], Richard J. Samworth[3], Rita Horvath[1], and the AIMS Collaborative

Affiliations

[1] School of Clinical Medicine, University of Cambridge, Cambridge, UK

[2] School of Public Health, Faculty of Medicine, Imperial College London, London, UK

[3] Statistical Laboratory, Centre for Mathematical Sciences, University of Cambridge, Cambridge, UK

Correspondence to:

Tomas Ferreira

Cambridge Centre for Brain Repair, Ed Adrian Building, Cambridge CB2 0PY, UK

tf385@cam.ac.uk

Word count: 5,143

For peer review only

Abstract

Objective: To determine current United Kingdom (UK) medical students' career intentions after graduation and upon completing the Foundation Programme (FP), and to ascertain the motivations behind these intentions.

Design: Cross-sectional, mixed-methods survey of UK medical students, using a non-random sampling method.

Setting: All 44 UK medical schools recognised by the General Medical Council.

Participants: All UK medical students were eligible to participate. The study sample consisted of 10,486 participants, approximately 25.50% of the medical student population.

Outcome measures: Career intentions of medical students post-graduation and post-Foundation Programme, motivations behind these career intentions, characterising the medical student population and correlating demographic factors and propensity to leave the National Health Service (NHS).

Results: The majority of participating students (8,806/10,486, 83.98%) planned to complete both years of the FP after graduation, with under half of these students (4,294/8,806, 48.76%) intending to pursue specialty training thereafter. A subanalysis of career intentions after the FP by year of study revealed a significant decrease in students' intentions to enter specialty training as they advanced through medical school. Approximately a third of surveyed students (3,392/10,486, 32.35%) intended to emigrate to practise medicine, with 42.57% (n=1,444) of those students not planning to return. In total, 2.89% of students intended to leave medicine altogether (n=303). Remuneration, work-life balance, and working conditions were identified as important factors in decision-making regarding emigration and leaving the profession. Subgroup analyses

For peer review only

based on gender, type of schooling, fee type, and educational background were performed. Only 17.26% of surveyed students were satisfied or very satisfied with the overall prospect of working in the NHS.

Conclusions: The AIMS study highlights UK students' views and career intentions, revealing a concerning proportion of those surveyed considering alternative careers or emigration. Addressing factors such as remuneration, work-life balance, and working conditions may increase retention of doctors and improve workforce planning efforts.

Strengths and limitations of this study

- This study represents the largest ever survey of UK medical students, and the largest study investigating medical students' career intentions, providing valuable insights into their future plans.
- This comprehensive survey addresses a topical and critical issue, providing important findings with significant implications for the NHS.
- Due to the cross-sectional design of the study, it captures a 'snapshot' in time, and is thus unable to reflect changes in students' career intentions over time.
- A high consent rate of 71.29% for follow-up studies allows for the possibility of longitudinal validation and observation of changes over time.
- Despite being the largest study of UK medical students, approximately 25.50% of the eligible UK medical students participated, which may introduce selection bias, as it may be that the survey appealed to those already intending to leave the NHS or who were interested in this topic; moreover, a comparison of the survey sample with contemporary demographic data was not possible, as the most recent available data on medical students dated back to 2018.

For peer review only

Introduction

Training doctors is a costly investment, and measuring the extent of attrition from the health service in the country of training is crucial to ensure optimal value. Understanding medical students' career plans and trajectories post-graduation is an important factor in effective workforce planning and retention.

There are several factors behind doctors' motivations to emigrate to practise medicine abroad or leave the profession entirely. Commonly cited themes among doctors in the United Kingdom (UK) include pay erosion

For peer review only - http://bmjopen.bmj.com/site/about/guidelines.xhtml

and low pay compared to alternative destinations, working conditions within the National Health Service (NHS), wellbeing, work-life balance, and better training opportunities abroad (1, 2).

The UK has 3.2 doctors for every 1,000 people, ranking 25th amongst the Organisation for Economic Co-operation and Development (OECD) countries. This figure also represents the lowest number of doctors per capita among European countries in the OECD (3). The British government has responded to the issue of an insufficient number of doctors by opening new medical schools and expanding the student capacity of existing ones (4, 5). Recently, there have been proposals to double the number of medical school places as a solution to address the shortage of doctors in the NHS (6). However, without addressing the issue of doctors leaving the NHS, increasing the number of medical students is unlikely to provide a sustainable long-term solution. Recruitment efforts may be ineffective if the retention of doctors is not simultaneously addressed. This highlights the pressing need for a multifaceted approach that considers both recruitment and retention strategies to effectively address the workforce challenges in the NHS.

Medical Education in the UK

In the UK, after medical school, medical graduates enter the Foundation Programme, a two-year programme consisting of a series of 4-month or 6-month rotations through various specialties and clinical settings. The successful completion of the programme's first year (FY1) provides doctors with full registration with the UK's medical regulator, the General Medical Council (GMC). This registration is recognised internationally. In many cases, individuals who leave the NHS after FY1 rather than immediately following graduation may do so because of the opportunities available with the full registration upon completing FY1. Completion of the second year of the programme (FY2) allows applicants to apply for specialist training pathways, such as those in psychiatry, neurosurgery, and general practice (7, 8).

To the best of our knowledge, this is the largest study of UK medical students to date. This mixed-methods study aimed to investigate current medical students' career intentions after graduation and upon completing the Foundation Programme, and the motivations behind these intentions. Secondary outcomes included determining which demographic factors alter the propensity to pursue different career paths available to a medical graduate, determining which specialties medical students plan to pursue and understanding current views on the prospect of working in the NHS. These data provide important answers to the current workforce challenges within the NHS and could help address some of the concerns of those making up the future of the profession.

Methods

Study design

For peer review only - http://bmjopen.bmj.com/site/about/guidelines.xhtml

AIMS (Ascertaining the career Intentions of UK Medical Students) was a national, multi-centre, cross-sectional study of medical students conducted in accordance with its published protocol (9). The study employed a non-random sampling method to recruit participants from 44 UK medical schools recognised by the General Medical Council (GMC).

A novel, self-administered, 71-item questionnaire was developed. The survey was hosted on the Qualtrics survey platform (Provo, Utah, USA), a GDPR-compliant online platform that supports both mobile and desktop devices. Prior to completing the survey, all participants provided informed consent. All participants were asked to complete the first section of the survey (Questions 1 to 11). Subsequent question visibility was dependent on participants' answers to previous questions. The fewest number of items available to any one participant was 30, and the largest was 43. Questions were structured using a combination of Likert scale matrices, multiple-choice options, and free-text entry to broaden the capture of sentiment nuance and improve precision in the data. A copy of the questionnaire and the Participant Information Sheet can be found in the Supplemental Materials.

Participant recruitment and eligibility

To minimise bias, a network of approximately 200 collaborators was recruited across 42 medical schools prior to the study launch to ensure equitable access to the survey. All medical students in all year groups were eligible to apply, and positions were advertised via medical student societies, social media, and internal medical school newsletters. They were responsible for maximising the response numbers within their year group at their medical schools. Collaborators were instructed to use a range of distribution methods, including social media, internal bulletins/newsletters, and email communication. This approach aimed to achieve a representative sample and improve the generalisability of our findings.

In order to qualify for collaborative authorship, students were required to achieve a minimum of 35 responses, or 15% of their year group (whichever number was lowest). The survey was disseminated between January 16, 2023, and March 27, 2023, by the AIMS Collaborative.

To be eligible for participation, individuals must have been actively enrolled in a UK medical school acknowledged by the General Medical Council (GMC) and listed by the Medical School Council (MSC) (Supplemental Materials). Certain new medical schools have received approval from the GMC but were yet to admit their inaugural cohort of students at the time of data collection. As they had no medical students, these schools were therefore excluded from our study.

Data collection

For peer review only - http://bmjopen.bmj.com/site/about/guidelines.xhtml

The survey consisted of five parts. Part 1 involved a background and demographics section, which all participants were required to answer. In Part 2, participants were asked to indicate their intended career paths immediately after graduation and after foundation training (if applicable). Part 3 explored the factors influencing their decision-making. Part 4 surveyed their current specialty preferences. The final part featured a free-entry text box inviting participants to articulate how the prospect of working in the NHS could be improved. Consent for follow-up studies was also obtained in this section.

Data processing and storage

Each response was restricted to a single institutional email address to mitigate the risk of data duplication. Any replicated email entries were removed prior to data analysis. In cases where identical entries contained distinct responses, the most recent entry was retained. Entries where respondents did not provide a valid institutional email address were removed prior to data analysis to preserve the integrity of the study.

Quantitative data analysis

Descriptive analysis was carried out with Microsoft Excel (v16.71) (Arlington, Virginia, USA), and statistical inference was performed using RStudio (v4.2.1) (Boston, Massachusetts, USA). Tables and graphs were generated using GraphPad Prism (v9.5.0) (San Diego, California, USA). Odds ratios (OR), confidence intervals (CI) and p-values were computed by fitting single-variable logistic regression models to explore the effect of various demographic characteristics on students' career intentions. Confidence intervals were calculated at 95% level. We used $p<0.05$ to determine the statistical significance for all tests.

The findings of this study were reported in accordance with the STROBE (Strengthening the Reporting of Observational Studies in Epidemiology) guidelines (10).

Planned subsequent analyses

The comprehensive scope of the AIMS questionnaire requires separate analyses for different components. Future works will specifically focus on the data obtained in parts 4 (specialty preference) and 5 (qualitative responses) of the survey. This approach ensures robust evaluation of these data and their implications, with a full thematic analysis planned for the qualitative data collected.

Patients and public involvement

For peer review only

In the preparatory phase of the study, an informal focus group convened, comprised of medical students at various training stages. These students contributed insights on potential negative aspects of the medical profession within the UK, posited as potential influences on decisions to pause or leave medical training in the UK. In addition, advice was sought from senior clinicians on this topic, providing a more balanced understanding of the issues at hand.

Results

Demographics

In total, 10,486 students across all 44 medical schools in the UK participated in the survey (Supplemental Figure 1). This represents approximately 25.50% of the medical student population in the UK (n=41,860), according to the latest accessible GMC report on medical student numbers (11). The mean response number per medical school was 244, and the median was 203 (IQR 135-281). A breakdown of the response numbers per medical school can be found in the Supplemental Materials. The median age for participants was 22 (IQR 20-23). Although responses were obtained from all year groups, there were relatively fewer responses from students in the 'Year 4 (not penultimate year)' category, likely due to a smaller number of students in intercalating courses or schools with six-year medical programmes, rather than the conventional five-year curriculum. Among the participants, 66.5% were female (n=6,977), 32.7% were male (n=3,429), 0.6% were non-binary (n=64), and 16 individuals preferred not to disclose their gender (Table 1).

Table 1. Demographic characteristics of participants

Characteristic	Number (%)
Ethnicity	
White	5,838 (55.67)
Asian or Asian British	3,027 (28.87)
Black, Black British, Caribbean or African	529 (5.04)
Mixed or multiple ethnic groups	555 (5.29)
Other	410 (3.91)
Prefer not to say	127 (1.21)
Gender	

For peer review only

For peer review only - http://bmjopen.bmj.com/site/about/guidelines.xhtml

Female	6,977 (66.54)
Male	3,429 (32.70)
Non-binary	64 (0.61)
Prefer not to say	16 (0.15)
Level of education	
Postgraduate	1,873 (17.86)
Undergraduate	8,613 (82.14)
Previous schooling	
Private education	3,605 (34.38)
State education	6,609 (63.03)
Prefer not to say	272 (2.59)
Fee status	
Home	9,207 (87.80)
EU	419 (4.00)
International (Non-EU)	860 (8.20)
Current year of study	
Year 1	1,963 (18.72)
Year 2	2,152 (20.52)
Year 3	1,952 (18.62)
Year 4 (not penultimate year)	947 (9.03)
Penultimate year	1,989 (18.97)
Final year	1,483 (14.14)
Age	
Median (range)	22 (17-48)
Total	10,486 (100.00)

Career intentions

All participants were asked their current career intention for immediately after graduation, as shown in Supplemental Table 1. The majority of participating students (8,806/10,486, 83.98% (CI: 83.26%, 84.67%)) planned to complete both years of the UK's foundation training, Foundation Years 1 (FY1) and 2 (FY2); 10.50% (CI: 9.93%, 11.10%) intended to complete FY1 and then emigrate to practise medicine (n=1,101); 1.26% (CI: 1.06%, 1.49%) planned to complete FY1 and then permanently leave the profession (n=132); 0.99% (CI: 0.82%, 1.20%) intended to leave medicine permanently immediately after graduation (n=104); 2.10% (CI: 1.84%, 2.39%) planned to emigrate to practise medicine abroad immediately after graduation (n=220); and 1.17% (CI: 0.98%, 1.40%) intended to take a break or undertake further study post-graduation (n=123).

Participants intending to complete both years of the Foundation Programme were then asked their intentions thereafter; the results can be seen in Supplemental Table 2. Of these 8,806 respondents, 48.76% (n=4,294, CI: 47.72%, 49.81%) planned to enter specialty training in the UK immediately after the Foundation Programme; 21.11% (n=1,859, CI: 20.27%, 21.98%) intended to enter a non-training clinical job in the UK (a common form of 'F3' year, including posts such as junior clinical fellowship or clinical teaching fellowship, or working as a locum doctor). These positions, while clinical in nature and valuable for gaining practical experience, do not typically contribute to full accreditation within a medical specialty, and are thus termed 'non-training'. A further 23.52% of participating students (n=2,071, CI: 22.64%, 24.42%) intended to emigrate to practise medicine abroad, whilst 5.85% (n=515, CI: 5.38%, 6.36%) planned to take a break or undertake further study. 67 of the participating students (0.76%, CI: 0.60%, 0.97%) planned to leave medicine permanently after FY2.

A total of 32.35% of the surveyed medical students (n=3,392/10,486, CI: 31.46%, 33.25%) intended to emigrate to practise medicine, either immediately after graduation (n=220/3,292, 6.49%, CI: 5.71%, 7.36%), after completion of FY1 (n=1,101/3,292 32.46%, CI: 30.90%, 34.05%) or after FY2 (n=2,071/3,292, 61.06%, CI: 59.40%, 62.68%). These students were asked their likelihood of their return to UK medicine (return prospects): 49.56% (n=1,681, CI: 47.88%, 51.24%) planned to return after a few years, whilst 7.87% (n=267, CI: 7.01%, 8.83%) intended to return after completion of their medical training abroad. The remaining 42.57% (n=1,444, CI: 40.92%, 44.24%) of those participating students planning on emigrating indicated no intentions to return (Supplemental Figure 2a). Of those favouring emigration immediately after graduation, 80.91% did not intend to return to the UK (n=178/220, CI: 75.20%, 85.55%). This number decreased to 60.03% (n=661/1101, CI: 57.11%, 62.89%)) in those planning to emigrate after completing FY1, and 29.21% (n=605/2071, CI: 27.29%, 31.21%) in those planning to emigrate after completing FY2, as demonstrated in Supplemental Figure 2b.

For peer review only - http://bmjopen.bmj.com/site/about/guidelines.xhtml

All participating students intending to emigrate to practise medicine were asked the countries to which they were considering emigrating via a free-entry text box. Students were able to list multiple locations or express if they were undecided. A total of 4,115 responses were received from 3,392 students. 25.03% (n=849) did not express a preference for any particular destination (Figure 1). The remaining 2,543 medical students listed 3,266 destination preferences. Australia was the most commonly mentioned destination (42.35%), followed by New Zealand (18.03%), the United States (10.38%) and Canada (10.29%).

A total of 303/10,486 (2.89%, CI: 2.59%, 3.23%) of surveyed medical students planned to leave the profession entirely, either immediately after graduating (n=104/303, 34.32%, CI: 29.20%, 39.84%), after completion of FY1 (n=132/303, 43.56%, CI: 38.1%, 49.19%), or after completion of FY2 (n=67/303, 22.11%, CI: 17.8%, 27.12%). Students intending to leave the profession were asked the alternative industries they were considering for their future careers (Figure 1). 21.12% (n=64/303) of those planning to leave the profession did not yet have an industry in mind. Of the remaining 78.88%, career destinations mentioned most often included consulting, technology, financial services, and law.

Career intention subanalyses

Subanalysis of career intentions after graduation by year of study revealed an overall increase in the proportion of surveyed students intending to complete the Foundation Programme as they progressed in their medical studies (Supplemental Figure 3). Supplemental Tables 3 and 4 highlight the surveyed students' career intentions after graduation and Foundation Programme, respectively, by year group.

Subanalysis of career intentions after completion of FY2 by current year of study revealed a significant decrease in the proportion of surveyed students looking to enter specialty training as they progressed in their medical studies (Supplemental Table 4). By contrast, intentions to emigrate, permanently leave the profession and assume non-training clinical positions also increased as students advanced through medical school (Figure 2).

Subanalysis of the subgroup intending to leave medicine (n=303, 2.89%) revealed a significant difference in the proportion of surveyed students taking this decision by various demographic characteristics, as highlighted in Table 2. Specifically, males were significantly more likely to plan to leave medicine than females (OR 2.61, CI [2.08, 3.30], p<0.00001), and state-educated students had a higher likelihood of planning to leave medicine compared to privately educated students (OR 1.28, CI: [1.01, 1.62], p=0.04). However, no statistically significant difference between home students and non-home students, including international and EU students, was identified (OR 1.26, CI [0.71, 2.06], p=0.39). Similarly, we did not find a statistically significant

For peer review only - http://bmjopen.bmj.com/site/about/guidelines.xhtml

difference between undergraduates and postgraduates in their likelihood of planning to leave medicine (OR 1.29, CI [0.94, 1.80], p=0.124).

We subanalysed the group of surveyed students intending to emigrate to practise by ethnicity, gender, stage of training, educational background, and previous schooling (Table 2*)*. Males were significantly more likely to plan to emigrate to practise medicine than females (OR 1.17, CI [1.07, 1.27], p<0.001). Postgraduate students were significantly more likely to plan to emigrate to practise medicine than undergraduate students (OR 1.20, CI [1.08, 1.33], p<0.001). Privately educated students were significantly more likely to plan to emigrate to practise medicine than their state educated peers (OR 1.26, CI [1.15, 1.37], p<0.00001). Non-home students (international and non-EU fees) were considerably more likely to plan to emigrate to practise medicine than home students (OR 2.33, CI [1.92, 2.84], p<0.00001).

Table 2. Demographic subanalysis of students intending to leave the medical profession and of students intending to emigrate to practise medicine

Demographic subgroup	Number intending to leave medicine (%)	Number intending to
Ethnicity		
White	147 (2.52)	1,938 (33.
Asian or Asian British	99 (3.27)	911 (30.1
Black, Black British, Caribbean or African	15 (2.84)	176 (33.2
Mixed or multiple ethnic groups	24 (4.32)	191 (34.4
Other	10 (2.44)	141 (34.3
Prefer not to say	8 (6.30)	35 (27.5
Gender		
Female	134 (1.92)	2,183 (31.
Male	167 (4.87)	1,191 (34.

For peer review only - http://bmjopen.bmj.com/site/about/guidelines.xhtml

Non-binary	1 (1.56)	12 (18.7[illegible]
Prefer not to say	1 (6.25)	6 (37.5[illegible]
Level of education		
Postgraduate	44 (2.35)	669 (35.[illegible]
Undergraduate	259 (3.01)	2,723 (31.[illegible]
Previous schooling		
Private education	118 (3.27)	1,287 (35.[illegible]
State education	170 (2.57)	2,024 (30.[illegible]
Prefer not to say	15 (5.51)	81 (29.7[illegible]
Fee status		
Home	276 (3.00)	2,774 (30.[illegible]
EU	15 (3.58)	217 (51.[illegible]
International (non-EU)	12 (1.40)	401 (46.[illegible]
Current year of study		
Year 1	21 (1.07)	645 (32.[illegible]
Year 2	42 (1.95)	713 (33.[illegible]
Year 3	53 (2.72)	596 (30.[illegible]
Year 4 (not penultimate year)	46 (4.86)	326 (34.[illegible]
Penultimate year	75 (3.77)	616 (30.[illegible]
Final year	66 (4.45)	396 (33.[illegible]
Total	303 (100.00)	3,392 (10[illegible]

For peer review only - http://bmjopen.bmj.com/site/about/guidelines.xhtml

We also performed demographic subanalysis on participating students' likelihood to return to the UK if emigrating abroad (Supplemental Table 5). Males were significantly less likely to plan to return to the UK after emigrating to practise medicine than females (OR 0.65, CI [0.56, 0.75], p<0.00001). Postgraduates were less likely to plan to return to the UK after emigrating to practise medicine than undergraduates (OR 0.85, CI [0.71, 1.00], p=0.05). Privately educated students were significantly less likely to plan to return to the UK after emigrating to practise medicine than state educated students (OR 0.77, CI [0.67, 0.89], p<0.001). Non-home students (international and EU fees) were significantly less likely to plan to return to the UK after emigrating to practise medicine than home students (OR 0.18, CI [0.14, 0.23], p<0.00001).

Reasons for students' decisions and overall view of aspects of working in the NHS

Once surveyed students had indicated their intended career option, they were asked the importance behind each of the factors below in their decision to do so. A series of Likert scale matrices were used, with options varying from 'Very important' to 'Not at all important'. The elements used in the matrices were compiled by the authors through a review of academic and grey literature, social media, and input from other clinicians. Students' reasons for planning to leave the NHS, either by emigrating or leaving the profession entirely, can be found in Figures 1a and 1b. For those not entering either the Foundation Programme or specialty training immediately after completion of medical school or foundation training, burnout, and the ability to choose their working location were the most important factors in this decision. The full results can be found in Supplemental Figures 4 and 5.

Remuneration at junior level, work-life balance, autonomy over choice of location and the working conditions of doctors in the NHS were identified as the most important factors for surveyed students intending to emigrate to practise medicine (Figure 2a). This was also the case for those planning to leave medicine, with the addition of nearly 82% of surveyed students listing burnout as an important or very important reason to abandon the profession (Figure 2b).

To better ascertain the surveyed student population's overview of working in the NHS, participants were asked to share their degree of satisfaction with several aspects of working in the NHS. Likert scale matrices were again used in a similar fashion, with options ranging from 'Very satisfied' to 'Not at all satisfied'. Figure 3 illustrates these findings. Less than 6% of the surveyed medical student population reported feeling satisfied or very satisfied with remuneration at junior level, work-life balance, working conditions of a doctor in the NHS, and costs associated with training (such as fees for professional/regulatory body memberships and examinations). A sizeable proportion of participants responded with a neutral rating, neither satisfied nor unsatisfied, when asked about certain aspects of their prospective medical training. Specifically, these aspects included pension tax rules as a consultant, theatre time during the Foundation Programme, and exposure to

For peer review only - http://bmjopen.bmj.com/site/about/guidelines.xhtml

their desired specialty during the Foundation Programme. In cases where participants may not have held strong opinions on a particular aspect, they tended to select the neutral option. Notably, however, only 17.26% of surveyed students were satisfied or very satisfied with the overall prospect of working in the NHS.

Discussion

Principal findings

Our findings demonstrate that a high proportion of the surveyed medical students intend to either leave the profession or permanently emigrate to practise medicine. To the best of our knowledge, there are no previous studies to which to compare these results, so it is difficult to gauge how these figures may have changed over time. We have observed that with each successive year of medical school, the students in our sample became less inclined to enter specialty training in the UK without a break, or at all. Specifically, less than a quarter of final-year medical students surveyed intended to enter specialty training immediately after the Foundation Programme. In total, 35.23% of the surveyed medical students plan to leave the NHS within two years of graduating, either to practise abroad or to pursue other careers. Approximately 60% of the surveyed sample of UK medical students was either not satisfied or not at all satisfied with the prospect of working in the NHS.

Implications

The NHS is facing a critical workforce shortage, with approximately 10,000 doctors relinquishing their licence to practise in 2021, representing a loss of nearly one-tenth of the doctor workforce (5, 12). A British Medical Association (BMA) survey of 8,000 senior doctors determined that 44% of NHS consultants in England plan to leave or take a break from working in the NHS over the next year (13). Similarly, a recent survey of 4,553 junior doctors in the NHS reported that 4 in 10 plan to leave the NHS, with 33% of these wanting to emigrate to another country to work (14). The combination of these previous surveys of the doctor workforce, and the results of our medical student survey suggest this trend is presently unlikely to improve. The GMC has recognised the problem and called for immediate action to mitigate the exodus of doctors from the NHS to more attractive employers (15).

Countries within the anglosphere, namely Australia, New Zealand, the United States and Canada, were the most widely cited destinations for students intending to emigrate. This is perhaps unsurprising given the higher salaries, reports of improved work-life balance, and the fact that these countries' primary language is English (16). Our study's findings align with previous literature highlighting doctors' leading reasons for emigration, namely pay, working conditions, and work-life balance (1, 17).

For peer review only - http://bmjopen.bmj.com/site/about/guidelines.xhtml

This study highlights that a disconcerting proportion of participating students, 32.35% (CI: 31.46%, 33.25%), intend to emigrate to practise medicine, with nearly half of these students intending not to return. This represents a large proportion of the current cohort of medical students. Despite these figures, there remains great uncertainty in this area. It is important to note that a considerable number of students who initially express an intention to emigrate temporarily may ultimately choose to stay abroad permanently (17). Similarly, some students who do not intend to return to the UK may change their minds in the future. Students paying EU or international fees reported significantly higher intentions to emigrate permanently. The stage at which students intend to emigrate appears to be related to the likelihood of return. Importantly, our study suggests that the proportion of students who intend to leave the NHS may be underestimated, as more students express a desire to leave as they progress through medical school. Moreover, once students enter the Foundation Program, a proportion may decide to leave the NHS, even if they had not previously intended to do so.

Insights into the emigration intentions of medical students in other nations indicate that a substantial proportion express a desire to emigrate and practise medicine in countries such as the United States and Canada, as well as to the UK. For instance, in one study, it was found that 49.7% of Malagasy medical students and 25.2% of Tanzanian medical students expressed their intention to emigrate to practise (18). Similarly, in another study, it was revealed that 44.6% of Ugandan medical students planned to emigrate (19). It is interesting that the observed trends in these low- and middle-income countries align with those in the UK, despite the latter's significantly larger economy.

Our results indicate that 2.89% of the medical students participating in our study expressed intentions to quit medicine. A study conducted in Kazakhstan identified a similar trend, with 4% of the participants expressing a desire to leave the medical profession altogether (20). Additionally, again similar to our results, the study reported a pattern in which medical students in junior years were less inclined to express a desire to leave the profession compared to students in senior years (20).

In addition to the 35.24% of sampled medical students intending to quit the NHS within two years of graduating, a considerable proportion of participating students (21.11%, CI: 20.27%, 21.98%) intended to assume a non-training clinical position in the UK after completing the Foundation Programme. Participants reported motivations for working in a non-training clinical post in keeping with existing literature surrounding the ‘F3’ year, with burnout, the ability to choose work location, travel and a greater earning potential evidently being the most compelling reasons to do so (21, 22). Furthermore, in this aspect, we report an increase in intention to not take up specialty posts immediately after the Foundation Programme, with an increase from 6.75% (CI: 5.62%, 8.08%) of first-year students to 35.98% (CI: 33.45%, 38.59%) of final year students. A contributing factor to this scenario could be a significant increase in competition ratios for specialty training posts, partly due to increasing medical student places and no corresponding increase in the number of training

For peer review only

posts available (for example, neurosurgery ST1 competition ratio was 3.9 in 2013 vs 15.94 in 2022) (23). Without corresponding increases to specialist training posts, increases in medical school places may be ineffective in doctor retention.

Historically, the vast majority of medical graduates pursued specialty training immediately after completing their Foundation Programme; for instance, in 2010, 83.1% of doctors entered specialty training after completing FY2. However, after steadily decreasing year-on-year, this percentage was only 34.9% of doctors in 2019 (7). The UK Foundation Programme Office has not repeated the survey since then, so surmising how these statistics may have changed in the interim is difficult. Our findings indicate that less than half of the medical students surveyed intended to enter specialty training after the Foundation Programme, with a negative correlation between medical student seniority and intention to enter specialty training with no break, or at all. Only 25.80% of participating final-year students intended to do so. In the UKFPO survey, those doctors had experienced the negative aspects of the profession. As such, it is concerning to observe this decline in interest among medical students, who have yet to formally begin their careers in medicine.

The findings of our study also align with existing literature on the factors influencing junior doctors' career decisions. Consistently, previous research emphasises the significance of working conditions, location, and earnings in shaping these decisions (1, 2, 21, 22, 24-27). Challenging work environments, long hours, and inadequate support contribute to disillusionment, burnout, and a desire to pursue alternative career paths (28). Similarly, the autonomy to choose work location emerges as a key factor in medical students, echoing findings among junior doctors. Earnings have consistently been identified as an influential factor for both junior doctors and medical students (1, 2, 16, 17, 21-27). Financial considerations impact their quality of life, student loan repayments, and long-term financial stability. The allure of higher salaries and better earning potential in other healthcare systems or professions can attract medical graduates away from NHS training programs. Addressing working conditions, providing career advancement opportunities, ensuring internationally competitive salaries, and considering location preferences can improve the ability to attract and retain talented professionals. Our study contributes to the growing body of literature by including medical students and supports the notion that working conditions, location, and earnings are significant factors influencing junior doctors' decisions to enter or remain in training. These findings underscore the importance of addressing these factors to create a supportive and appealing environment for junior doctors, ultimately promoting better retention rates within the NHS.

Furthermore, our results suggest that the recent calls for dramatic increases in medical school places are unlikely to resolve the NHS staffing shortages. The MSC responded to the original call to increase places by 5,000 students by stating multiple barriers, including cost, clinical placement capacity and the lack of a strategic approach to growth. It is estimated that to increase medical schools' capacity by just 5,000 places, approximately £1 billion per annum would be required (29). Additionally, the training of medical students

For peer review only - http://bmjopen.bmj.com/site/about/guidelines.xhtml

heavily relies on clinical exposure, which in turn is dependent on availability of clinical teaching staff, facilities for training and opportunities (6). Without a corresponding increase in clinical placement capacity, an increase in medical student places may lead to a decline in the standard of medical education. Our results indicate that increases in medical student places via expansion of existing medical schools or the establishment of new medical schools may not result in proportionate increases in doctors wishing to remain in the NHS. Any attempts to reverse the NHS workforce challenge may benefit from prioritising doctor retention. In this paper, we have highlighted the reasons driving medical students to plan for careers outside of the NHS; addressing these problems is likely to result in improved retention rates.

While there have been studies that i) explore which specialties junior doctors or medical students intend on pursuing, and exploring factors attracting them to said specialties (30-52); ii) focus on reasons why doctors are leaving the UK (1, 2, 24, 53); iii) explore how medical students and junior doctors feel about specific aspects of working within the NHS (25-27, 54); and iv) investigate the desire for a career break post-FY2 (21, 22), there have been no recent, high-powered studies explicitly aimed at medical students, irrespective of current career ambitions or seniority, investigating overall career intentions and correlating it with demographic factors and medical student seniority. Any statistically significant differences in career intentions between demographic subgroups should be considered carefully and discussed within the correct context. Further studies are required to fully elucidate the reasons behind these disparities.

Limitations

When interpreting this study's results, there are important limitations to consider. Firstly, the study's cross-sectional nature means we are unable to gauge how students' career intentions may have changed or will change. To address this, we have asked all participants for consent to participate in an anticipated follow-up study, which will enable validation of responses and measurement of change over time; for this, we obtained a 71.29% consent rate.

While this study represents the largest ever study of UK medical students, it is worth noting that approximately 25.50% of the total population of medical students participated. Consequently, we cannot exclude the possibility of selection bias, both from students not seeing the study invitation and others electing not to participate. It may be that this survey appealed to those already intending to leave the NHS or who were interested in the topic. In the context of the UK's medical student population, females were seemingly overrepresented in our study despite concerted efforts to ensure equitable outreach during our study advertising phase (57.05% vs 66.50%, respectively) (11). However, the availability of recent demographic data for comparison is limited, with the most recent available data pertaining to the 2018 cohort of medical students (11).

For peer review only - http://bmjopen.bmj.com/site/about/guidelines.xhtml

Additionally, the questions in our survey instruct students to be definitive even when they might not yet have an idea of their career plans, particularly for those in the younger years of medical school. For purposes of brevity and mitigation of survey fatigue, the survey did not provide exhaustive response options. As a result, some decision-making factors may have been omitted. To address this, a free-entry text box was available for participants to supplement their answers. Finally, it should be emphasised that the respondents were medical students who may have limited knowledge of the realities of working in the NHS. Their current reported perceptions may change once they begin their careers in the NHS.

Conclusion

This study highlights that an alarming proportion of surveyed medical students intend to leave the profession or emigrate to practise medicine. The proportion of students in our sample who plan to leave the NHS within two years of graduating is considerable, representing a potential loss of valuable medical talent. Alarmingly, the majority of participating medical students were either not at all satisfied or not satisfied with the prospect of working in the NHS. Additionally, an increasing proportion of the surveyed students intended to take up non-training clinical positions, which could reduce the availability of highly skilled doctors in the NHS. The findings of this study emphasise the urgency of addressing the factors that are driving the exodus of doctors from the NHS and suggest that increased recruitment of medical students may not provide an adequate solution to staffing challenges. The causes of the problem are complex, and finding a solution will require a multifaceted approach. Steps could include improving work-life balance, increasing salaries, addressing the growing competition for specialty training posts, and promoting greater flexibility in career pathways. Undoubtedly, the continued loss of skilled professionals from the NHS represents a significant concern, so it is critical to consider means of reversing this trend.

For peer review only

Contributors

TF responsible for conceptualisation. TF responsible for obtaining funding and ethical approval. TF responsible for collaborator recruitment and management. TF responsible for project administration. TF responsible for writing the manuscript. TF and AMC responsible for qualitative analysis. OF and RJS responsible for statistical quantitative analysis. All authors responsible for editing and revising the manuscript. RH responsible for supervision. TF is the guarantor. All authors have read and approved the manuscript.

Collaborators

Mario K Teo, Crispin C Wigfield, Dania Al-Hashimi, Maeve K Mulchrone, Alisha Pervaiz, Heather A Lewis, Anson Wong, Buzz Gilks, Charlotte Casteleyn, Sara Kidher, Erin Fitzsimons-West, Tanzil Rujeedawa, Meghna Sreekumar, Eliza Wade, Juel Choppy-Madeleine, Yasemin Durmus, Olivia King, Yu Ning Ooi, Malvi Shah, Tan Jih Yih, Samantha Burley, Basma R Khan, Emma Slack, Rishik S Pilla, Jenny Yang, Vaishvi Dalal, Brennan L Gibson, Emma Westwood, Brandon S H Low, Sara R Sabur , Wentin Chen, Maryam A Malik, Safa Razzaq, Amardeep Sidki, Giulia Cianci, Felicity Greenfield, Sajad Hussain, Alexandra Thomas, Annie

For peer review only - http://bmjopen.bmj.com/site/about/guidelines.xhtml

For peer review only

Harrison, Hugo Bernie, Luke Dcaccia, Linnuel J Pregil, Olivia Rowe, Ananya Jain, Gregory K Anyaegbunam, Syed Z Jafri, Arthur Handscomb, Sudhanvita Arun, Alfaiya Hashmi, Ankith Pandian, Joseph R Nicholson, Hannah Layton-Joyce, Kouther Mohsin, Matilda Gardener, Eunice C Y Kwan, Emily R Finbow, Sakshi Roy, Zoe M Constantinou, Mackenzie Garlick, Clare L Carney, Samantha Gold, Bilal Qureshi, Daniel Magee, Grace Annetts, Khyatee Shah, Kholood T Munir, Timothy Neill, Gurpreet K Atwal, Anesu Kusosa, Anthony Vijayanathan, Mia Mäntylä, Momina Iqbal, Sara Raja, Tushar Rakhecha, Muhammad H Shah, Pranjil Pokharel, Ashna Anil, Kate Stenning, Katie Appleton, Keerthana Uthayakumar, Rajan Panacer, Yasmin Owadally, Dilaxiha Rajendran, Harsh S Modalavalasa, Marta M Komosa, Morea Turjaka, Sruthi Saravanan, Amelia Dickson, Jack M Read, Georgina Cooper, Wing Chi Do, Chiamaka Anthony-Okeke, Daria M Bageac, David C W Loh, Rida Khan, Ruth Omenyo, Aidan Baker, Imogen Milner, Kavyesh Vivek, Manon Everard, Wajiha Rahman, Denis Chen, Michael E. Bryan, Shama Maliha, Vera Onongaya, Amber Dhoot, Catherine L Otoibhi, Harry Donkin-Everton, Mia K Whelan, Claudia S F Hobson, Anthony Haynes, Joshua Bayes-Green, Mariam S Malik, Subanki Srisakthivel, Sophie Kidd, Alan Saji, Govind Dhillon, Muhammed Asif, Riya Patel, Jessica L Marshall, Nain T Raja, Tawfique Rizwan, Aleksandra Dunin-Borkowska, James Brawn, Karthig Thillaivasan, Zainah Sindhoo, Ayeza Akhtar, Emma Hitchcock, Kelly Fletcher, Lok Pong Cheng, Medha Pillaai, Sakshi Garg, Wajahat Khan, Ben Sweeney, Ria Bhatt, Madison Slight, Adan M I Chew, Cameron Thurlow, Kriti Yadav, Niranjan Rajesh, Nathan-Dhruv Mistry, Alyssa Weissman, Juan F E Jaramillo, William Thompson, Gregor W Abercromby, Emily Gaskin, Chloe Milton, Matthew Kokkat, Momina Hussain, Nana A. Ohene-Darkoh, Syeda T Islam, Anushruti Yadav, Eve Richings, Samuel Foxcroft, Sukhdev Singh, Vivek Sivadev, Guilherme Movio, Ellena Leigh, Harriet Charlton, James A Cairn, Julia Shaaban , Leah Njenje, Mark J Bishop, Humairaa Ismail, Sarah L Henderson, Daniel C Chalk, Daniel J Mckenna, Fizah Hasan, Kanishka Saxena, Iona E Gibson & Saad Dosani.

Transparency declaration

TF, the lead author (the manuscript's guarantor), affirms that the manuscript is an honest, accurate, and transparent account of the study being reported; that no important aspects of the study have been omitted; and that any discrepancies from the study have been explained.

Ethics approval and consent to participate.

Ethical approval was granted by the University of Cambridge Research Ethics Committee (reference PRE.2022.124) on January 5th, 2023. Prior to completing the survey, all participants provided informed consent.

Competing interests

All authors have completed the Unified Competing Interest form (available on request from the corresponding author) and declare: no support from any organisation for the submitted work; no financial relationships with any organisations that might have an interest in the submitted work in the previous three years; and no other relationships or activities that could appear to have influenced the submitted work.

Funding

For peer review only

Queens' College, University of Cambridge. The institution has had no role in the design of the study, nor collection, analysis, and interpretation of data and in writing the manuscript.

Acknowledgements

We would like to thank all students that participated in this study. We would also like to thank Mr Mario K Teo and Mr Crispin C Wigfield for their advice in the earlier stages of the study.

Data availability statement

No additional data available.

For peer review only

References

1. Lambert TW, Smith F, Goldacre MJ. Why doctors consider leaving UK medicine: qualitative analysis of comments from questionnaire surveys three years after graduation. Journal of the Royal Society of Medicine. 2018;111(1):18-30.
2. Surman G, Goldacre MJ, Lambert TW. UK-trained junior doctor'' intentions to work in UK medicine: questionnaire surveys, three years after graduation. Journal of the Royal Society of Medicine. 2017;110(12):493-500.
3. OECD (2023), Doctors (indicator). doi: 10.1787/4355e1ec-en (Accessed on 15 April 2023), Available from: https://data.oecd.org/healthres/doctors.htm
4. Rimmer A. Five medical schools are created in England in bid to increase home grown doctors. BMJ: British Medical Journal (Online). 2018;360.
5. General Medical Council. The state of medical education and practice in the UK. The workforce report 2022.
6. Phillips S MI. A roadmap to double medical school places. Dec 2022.
7. UKFPO. UK Foundation Programme 2019 F2 Career UKFPO 2019 F2 Career Destinations Survey. 2019.
8. De Souza B. Choosing your specialty Foundation training. BMJ. 2007;334(7601):s172-s3.
9. Ferreira T, Collins AM, Horvath R. Ascertaining the Career Intentions of Medical Students (AIMS) in the United Kingdom Post Graduation: Protocol for a Mixed Methods Study. JMIR Research Protocols. 2023 Jun 19;12(1):e45992.
10. Elm Ev. The Strengthening the Reporting of Observational Studies in Epidemiology (STROBE) statement: guidelines for reporting observational studies. Ann Inter Med. 2007;147:573-7.
11. General Medical Council. (2020). State of medical education and practice in the UK: 2020 reference tables about medical students. https://www.gmc-uk.org/-/media/documents/gmc-somep-2020-reference-tables-about-medical-students_pdf-84718237.pdf [Accessed July 26, 2023]
12. NHS Digital Health. NHS Workforce Statistics, December 2021 Doctors by Grade and Specialty. 2022.
13. BMA. Catastrophic crisis facing NHS as nearly half of hospital consultants plan to leave in next year, warns BMA2022. Available from: https://www.bma.org.uk/bma-media-centre/catastrophic-crisis-facing-nhs-as-nearly-half-of-hospital-consultants-plan-to-leave-in-next-year-warns-bma.
14. Waters A. A third of junior doctors plan to leave NHS to work abroad in next 12 months. British Medical Journal Publishing Group; 2022.
15. General Medical Council. Migration and the medical workforce 2022 [Available from: https://www.gmc-uk.org/news/news-archive/migration-and-the-medical-workforce.
16. Grant P. Physician job satisfaction in New Zealand versus the United Kingdom. The New Zealand Medical Journal (Online). 2004;117(1204).
17. Sharma A, Lambert TW, Goldacre MJ. Why UK-trained doctors leave the UK: cross-sectional survey of doctors in New Zealand. Journal of the royal society of medicine. 2012;105(1):25-34.
18. Chaet A, Fessehaie N, Rajaguru PR, Alavi Jusabani M, Randaoharison P, Samison L, Anderson U, Ramanantoanina P, Zafimar M, Numfor A, Hardaker WM. Comparing the drivers of medical student emigration intention across two African nations. Medical Education. 2021 Oct;55(10):1194-204.

For peer review only - http://bmjopen.bmj.com/site/about/guidelines.xhtml

19. Kizito S, Mukunya D, Nakitende J, Nambasa S, Nampogo A, Kalyesubula R, Katamba A, Sewankambo N. Career intentions of final year medical students in Uganda after graduating: the burden of brain drain. BMC medical education. 2015 Dec;15:1-7.
20. Faizullina K, Kausova G, Kalmataeva Z, Nurbakyt A, Buzdaeva S. Career intentions and dropout causes among medical students in Kazakhstan. Medicina. 2013 Jun;49(6):45.
21. Church HR, Agius SJ. The F3 phenomenon: Early-career training breaks in medical training. A scoping review. Medical Education. 2021;55(9):1033-46.
22. Hollis AC, Streeter J, Van Hamel C, Milburn L, Alberti H. The new cultural norm: reasons why UK foundation doctors are choosing not to go straight into speciality training. BMC Medical Education. 2020;20(1):1-9.
23. NHS Health Education England. Competition Ratios [Available from: https://medical.hee.nhs.uk/medical-training-recruitment/medical-specialty-training/competition-ratios].
24. Milner A, Nielsen R, Verdery AM. Brexit and the European National Health Service England Workforce: A Quantitative Analysis of Doctors' Perceived Professional Impact and Intentions to Leave the United Kingdom. Annals of global health. 2021;87(1).
25. Scanlan GM, Cleland J, Johnston P, Walker K, Krucien N, Skåtun D. What factors are critical to attracting NHS foundation doctors into specialty or core training? A discrete choice experiment. BMJ open. 2018;8(3):e019911.
26. Ryan C, Ward E, Jones M. Recruitment and retention of trainee physicians: a retrospective analysis of the motivations and influences on career choice of trainee physicians. QJM: An International Journal of Medicine. 2018;111(5):313-8.
27. Cleland JA, Johnston P, Watson V, Krucien N, Skåtun D. What do UK medical students value most in their careers? A discrete choice experiment. Medical Education. 2017;51(8):839-51.
28. House of Commons Health and Social Care Committee. (2021). Workforce burnout and resilience in the NHS and social care: Second Report of Session 2021–22 (Publication No. HC 22). [Available from: https://committees.parliament.uk/publications/6158/documents/68766/default/] Accessed July 8, 2023.
29. Medical Schools Council. The expansion of medical student numbers in the United Kingdom. 2021.
30. Singh A, Alberti H. Why UK medical students change career preferences: an interview study. Perspectives on medical education. 2021;10(1):41-9.
31. Misky AT, Shah RJ, Fung CY, Sam AH, Meeran K, Kingsbury M, et al. Understanding concepts of generalism and specialism amongst medical students at a research-intensive London medical school. BMC Medical Education. 2022;22(1):1-11.
32. Lambert TW, Smith F, Goldacre MJ. Career specialty choices of UK medical graduates of 2015 compared with earlier cohorts: questionnaire surveys. Postgraduate Medical Journal. 2018;94(1110):191-7.
33. Surman G, Lambert TW, Goldacre MJ. Trends in junior doctors' certainty about their career choice of eventual clinical specialty: UK surveys. Postgraduate medical journal. 2013;89(1057):632-7.
34. Svirko E, Goldacre MJ, Lambert T. Career choices of the United Kingdom medical graduates of 2005, 2008 and 2009: questionnaire surveys. Medical teacher. 2013;35(5):365-75.
35. Ibrahim M, Fanshawe A, Patel V, Goswami K, Chilvers G, Ting M, et al. What factors influence British medical students' career intentions? Medical teacher. 2014;36(12):1064-72.
36. Reid K, Alberti H. Medical students' perceptions of general practice as a career; a phenomenological study using socialisation theory. Education for Primary Care. 2018;29(4):208-14.
37. Rehman U, Sarwar MS, Brennan PA. Attitude of clinical medical students to Oral and Maxillofacial Surgery as a career: a perspective from two English Medical Schools. British Journal of Oral and Maxillofacial Surgery. 2022;60(4):448-53.
38. Barber S, Brettell R, Perera-Salazar R, Greenhalgh T, Harrington R. UK medical students' attitudes towards their future careers and general practice: a cross-sectional survey and qualitative analysis of an Oxford cohort. BMC medical education. 2018;18(1):1-9.
39. Oliver H, Hudson B, Oliver C, Oliver M. UK undergraduate aspirations and attitudes survey: do we have a perception problem in clinical radiology? Clinical Radiology. 2020;75(2):158. e15-. e24.
40. Emmanouil B, Goldacre MJ, Lambert TW. Aspirations to become an anaesthetist: longitudinal study of historical trends and trajectories of UK-qualified doctors' early career choices and of factors that have influenced their choices. BMC anesthesiology. 2017;17(1):1-9.
41. Barat A, Goldacre MJ, Lambert TW. Junior doctors' early career choices do not predict career destination in neurology: 40 years of surveys of UK medical graduates. BMC medical education. 2019;19(1):1-9.
42. Robinson T, Lefroy J. How do medical students' experiences inform their opinions of general practice and its potential as a future career choice? Education for Primary Care. 2022:1-9.
43. Tambyraja AL, McCrea CA, Parks RW, Garden OJ. Attitudes of medical students toward careers in general surgery. World journal of surgery. 2008;32(6):960-3.

For peer review only

44. Thomas A. What about forensic psychiatry as a career? Undergraduate and early post-graduate medical perspectives. Criminal Behaviour and Mental Health. 2012;22(4):247-51.
45. Smith F, Lambert TW, Pitcher A, Goldacre MJ. Career choices for cardiology: cohort studies of UK medical graduates. BMC medical education. 2013;13(1):1-8.
46. Maisonneuve JJ, Pulford C, Lambert TW, Goldacre MJ. Career choices for geriatric medicine: national surveys of graduates of 1974–2009 from all UK medical schools. Age and ageing. 2014;43(4):535-41.
47. Goodson AM, Payne KF, Tahim A, Cabot L, Fan K. Awareness of oral and maxillofacial surgery as a specialty and potential career pathway amongst UK medical undergraduates. The Surgeon. 2013;11(2):92-5.
48. Halder N, Hadjidemetriou C, Pearson R, Farooq K, Lydall GJ, Malik A, et al. Student career choice in psychiatry: findings from 18 UK medical schools. International Review of Psychiatry. 2013;25(4):438-44.
49. Goldacre MJ, Fazel S, Smith F, Lambert T. Choice and rejection of psychiatry as a career: surveys of UK medical graduates from 1974 to 2009. The British Journal of Psychiatry. 2013;202(3):228-34.
50. Pakpoor J, Handel AE, Disanto G, Davenport RJ, Giovannoni G, Ramagopalan SV. National survey of UK medical students on the perception of neurology. BMC medical education. 2014;14(1):1-5.
51. Sutton PA, Mason J, Vimalachandran D, McNally S. Attitudes, motivators, and barriers to a career in surgery: a national study of UK undergraduate medical students. Journal of surgical education. 2014;71(5):662-7.
52. Moore J, McDiarmid A, Johnston P, Cleland J. Identifying and exploring factors influencing career choice, recruitment and retention of anaesthesia trainees in the UK. Postgraduate medical journal. 2017;93(1096):61-6.
53. Wilson HC, Abrams S, Simpkin Begin A. Drexit: Understanding why junior doctors leave their training programs to train overseas: An observational study of UK physicians. Health Science Reports. 2021;4(4):e419.
54. Lachish S, Goldacre MJ, Lambert T. Associations between perceived institutional support, job enjoyment, and intentions to work in the United Kingdom: national questionnaire survey of first year doctors. BMC medical education. 2016;16(1):1-8.

Figures legends

Figure 1. - **a)** Importance of factors influencing medical students' intention to emigrate and practise medicine; **b)** Importance of factors influencing medical students' intention to leave the medical profession entirely and seek an alternative career; **c)** Locations cited as potential destinations by students who intend to emigrate to practise medicine; **d)** Preferred industries to work in by those intending to leave medicine. *Several respondents cited the Middle East or Gulf region rather than specifying which country; these responses were grouped with individual destinations in the region

Figures 2. Proportions of students by year of study (with 95% confidence intervals) intending to **a)** directly enter specialty training after FY2; **b)** emigrate to practise medicine after FY2; **c)** enter a non-training clinical post after FY2, for example as a locum doctor or clinical fellow; **d)** leave medicine permanently after FY2 to pursue an alternative career. "Year 4" represents students in their fourth year of study, but not their penultimate year. Percentages in figures reflect the proportion of students in each year group for each intention.

Figure 3.- Medical students' satisfaction levels regarding aspects of working as a doctor in the NHS

For peer review only - http://bmjopen.bmj.com/site/about/guidelines.xhtml

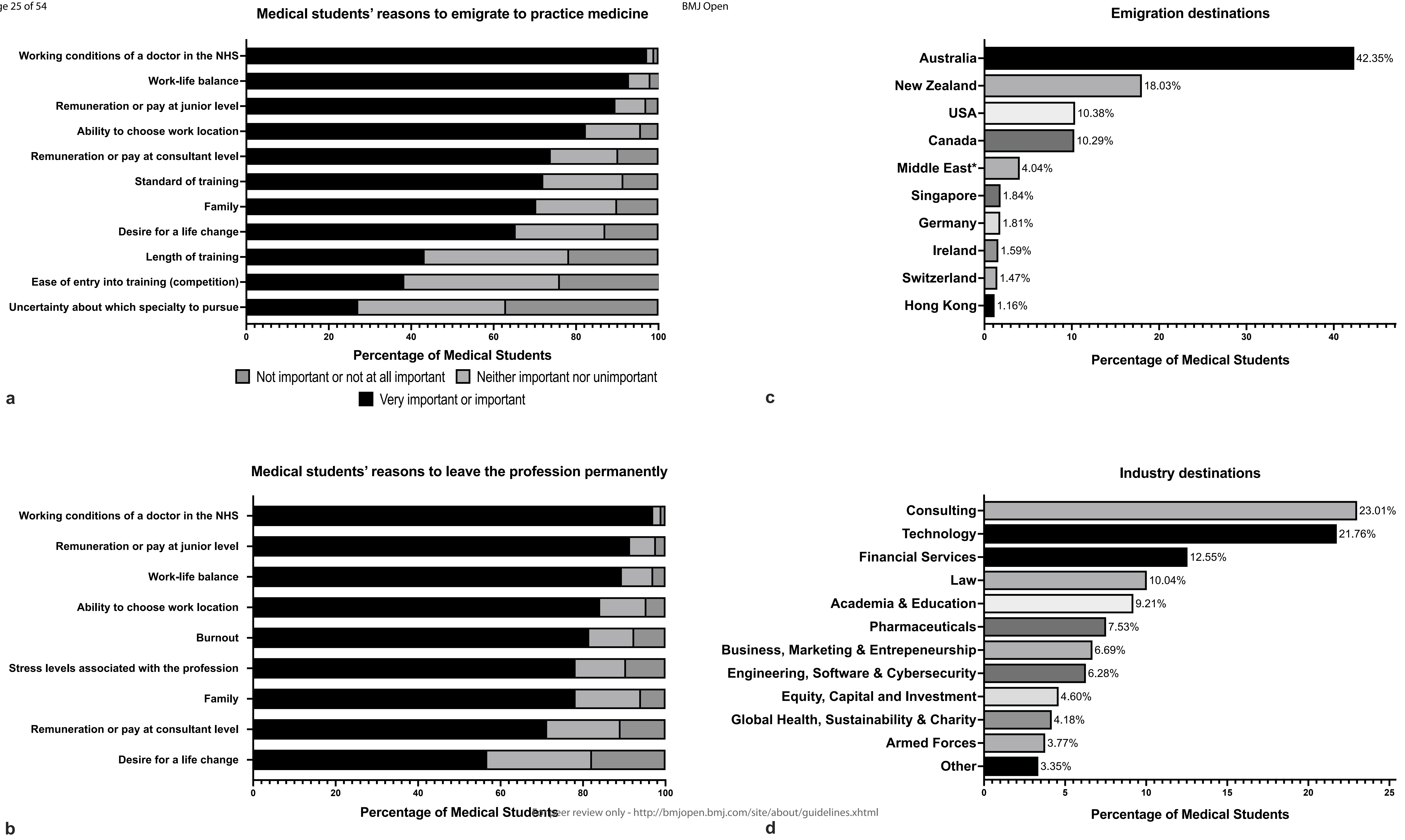

For peer review only - http://bmjopen.bmj.com/site/about/guidelines.xhtml

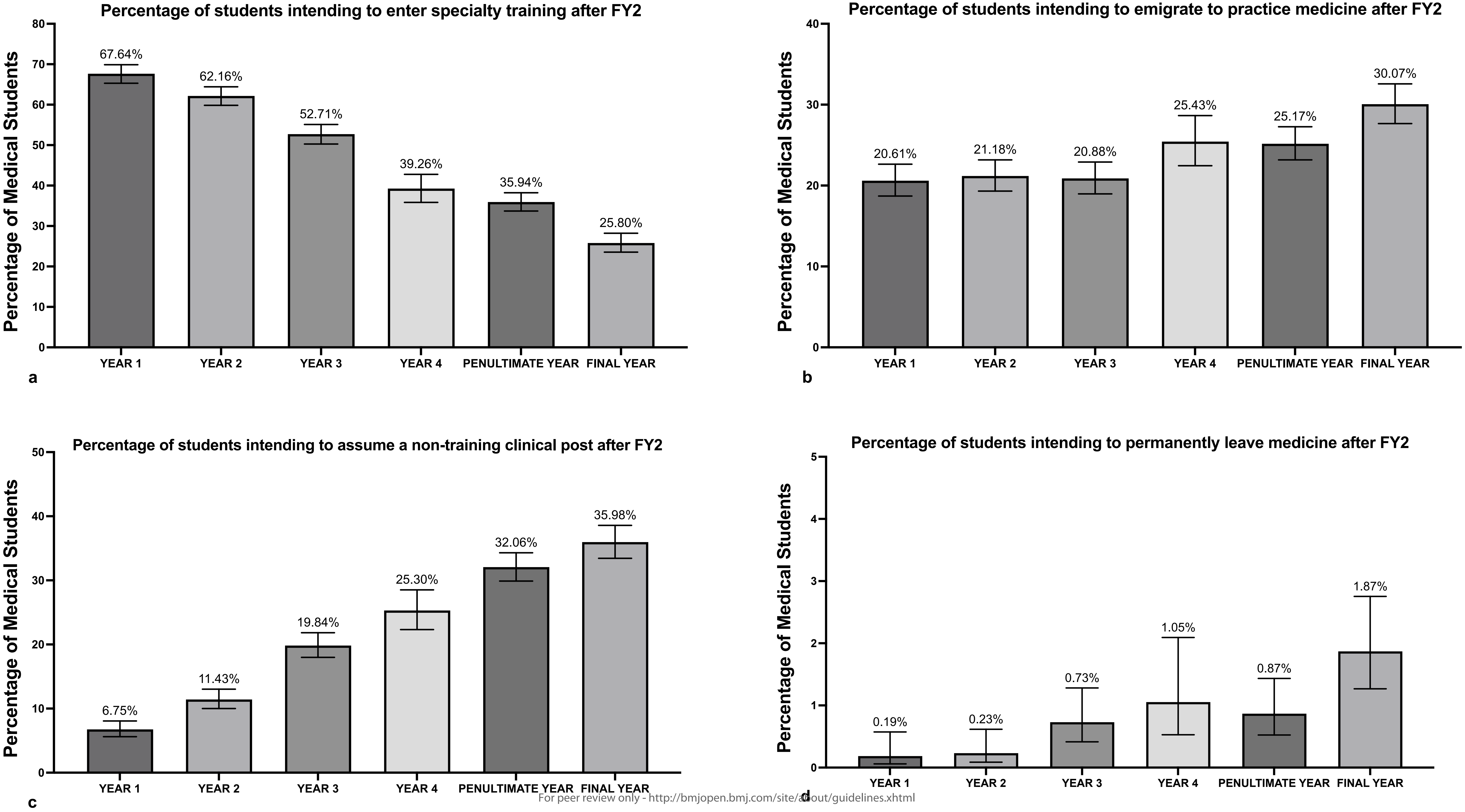
Percentage of students intending to enter specialty training after FY2
Percentage of Medical Students
80
70
60
50
40
30
20
10
0
67.64%
62.16%
52.71%
39.26%
35.94%
25.80%
YEAR 1
YEAR 2
YEAR 3
YEAR 4
PENULTIMATE YEAR
FINAL YEAR
a
Percentage of students intending to emigrate to practice medicine after FY2
Percentage of Medical Students
40
30
20
10
0
20.61%
21.18%
20.88%
25.43%
25.17%
30.07%
YEAR 1
YEAR 2
YEAR 3
YEAR 4
PENULTIMATE YEAR
FINAL YEAR
b
Percentage of students intending to assume a non-training clinical post after FY2
Percentage of Medical Students
50
40
30
20
10
0
6.75%
11.43%
19.84%
25.30%
32.06%
35.98%
YEAR 1
YEAR 2
YEAR 3
YEAR 4
PENULTIMATE YEAR
FINAL YEAR
c
Percentage of students intending to permanently leave medicine after FY2
Percentage of Medical Students
5
4
3
2
1
0
0.19%
0.23%
0.73%
1.05%
0.87%
1.87%
YEAR 1
YEAR 2
YEAR 3
YEAR 4
PENULTIMATE YEAR
FINAL YEAR
d

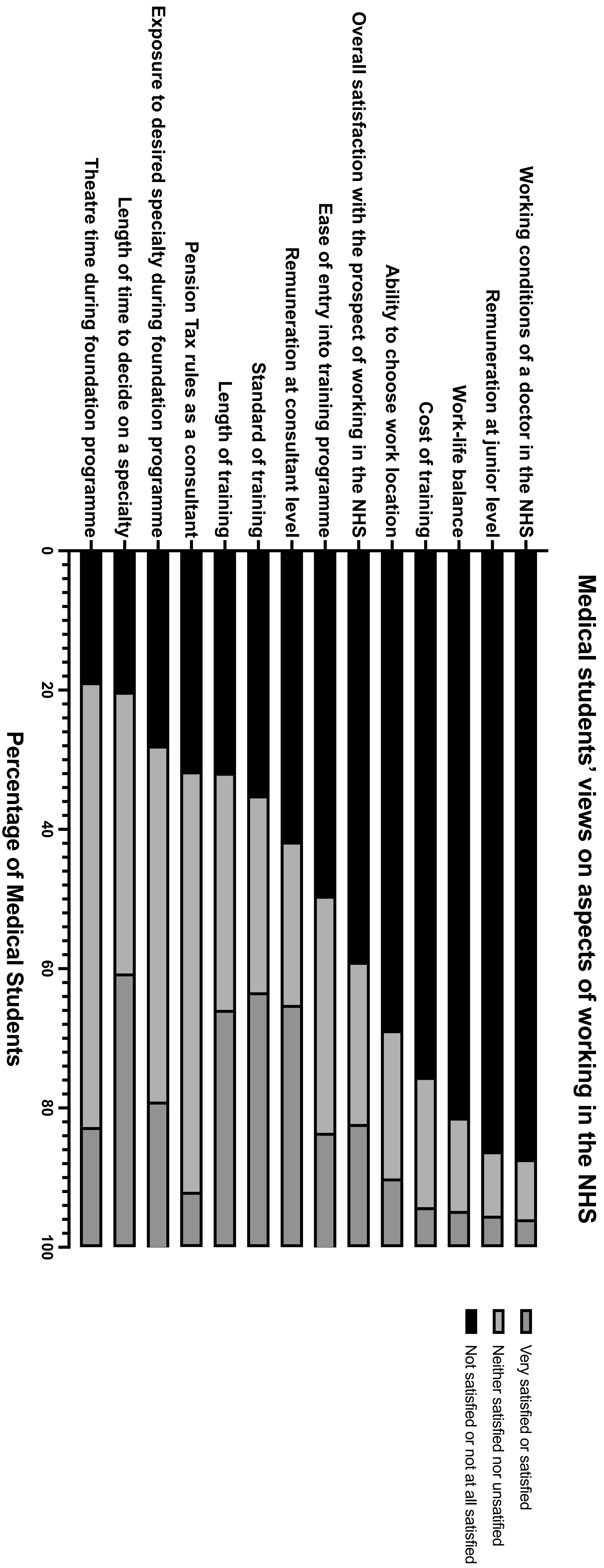
Medical students' views on aspects of working in the NHS
Working conditions of a doctor in the NHS
Remuneration at junior level
Work-life balance
Cost of training
Ability to choose work location
Overall satisfaction with the prospect of working in the NHS
Ease of entry into training programme
Remuneration at consultant level
Standard of training
Length of training
Pension Tax rules as a consultant
Exposure to desired specialty during foundation programme
Length of time to decide on a specialty
Theatre time during foundation programme
0
20
40
60
80
100
Percentage of Medical Students
Very satisfied or satisfied
Neither satisfied nor unsatified
Not satisfied or not at all satisfied

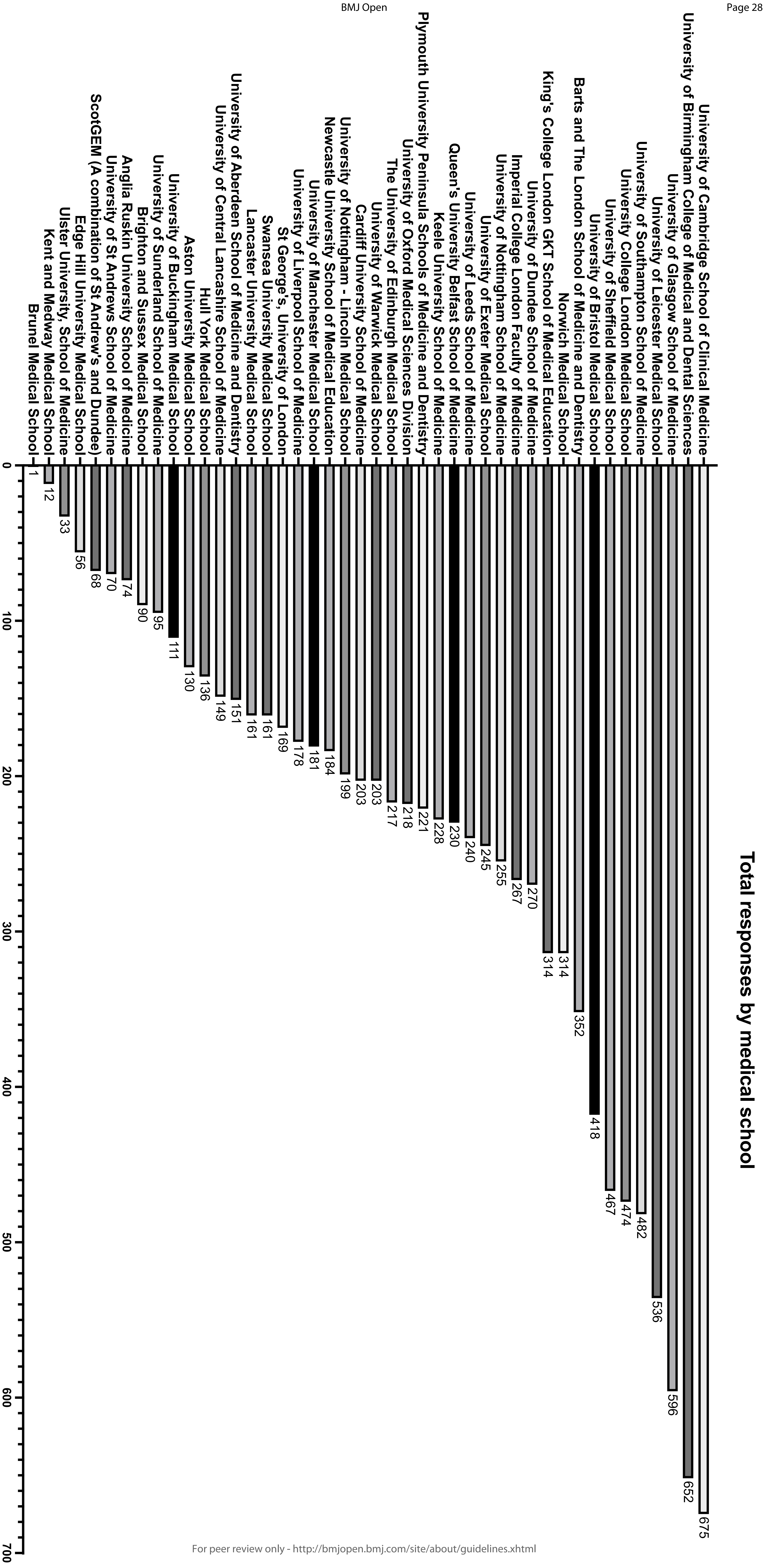
Total responses by medical school
University of Cambridge School of Clinical Medicine 675
University of Birmingham College of Medical and Dental Sciences 652
University of Glasgow School of Medicine 596
University of Leicester Medical School 536
University of Southampton School of Medicine 482
University College London Medical School 474
University of Sheffield Medical School 467
University of Bristol Medical School 418
Barts and The London School of Medicine and Dentistry 352
Norwich Medical School 314
King's College London GKT School of Medical Education 314
University of Dundee School of Medicine 270
Imperial College London Faculty of Medicine 267
University of Nottingham School of Medicine 255
University of Exeter Medical School 245
University of Leeds School of Medicine 240
Queen's University Belfast School of Medicine 230
Keele University School of Medicine 228
Plymouth University Peninsula Schools of Medicine and Dentistry 221
University of Oxford Medical Sciences Division 218
The University of Edinburgh Medical School 217
University of Warwick Medical School 203
Cardiff University School of Medicine 203
University of Nottingham - Lincoln Medical School 199
Newcastle University School of Medical Education 184
University of Manchester Medical School 181
University of Liverpool School of Medicine 178
St George's, University of London 169
Swansea University Medical School 161
Lancaster University Medical School 161
University of Aberdeen School of Medicine and Dentistry 151
University of Central Lancashire School of Medicine 149
Hull York Medical School 136
Aston University Medical School 130
University of Buckingham Medical School 111
University of Sunderland School of Medicine 95
Brighton and Sussex Medical School 90
Anglia Ruskin University School of Medicine 74
University of St Andrews School of Medicine 70
ScotGEM (A combination of St Andrew's and Dundee) 68
Edge Hill University Medical School 56
Ulster University, School of Medicine 33
Kent and Medway Medical School 12
Brunel Medical School 1
0
100
200
300
400
500
600
700
Total Responses

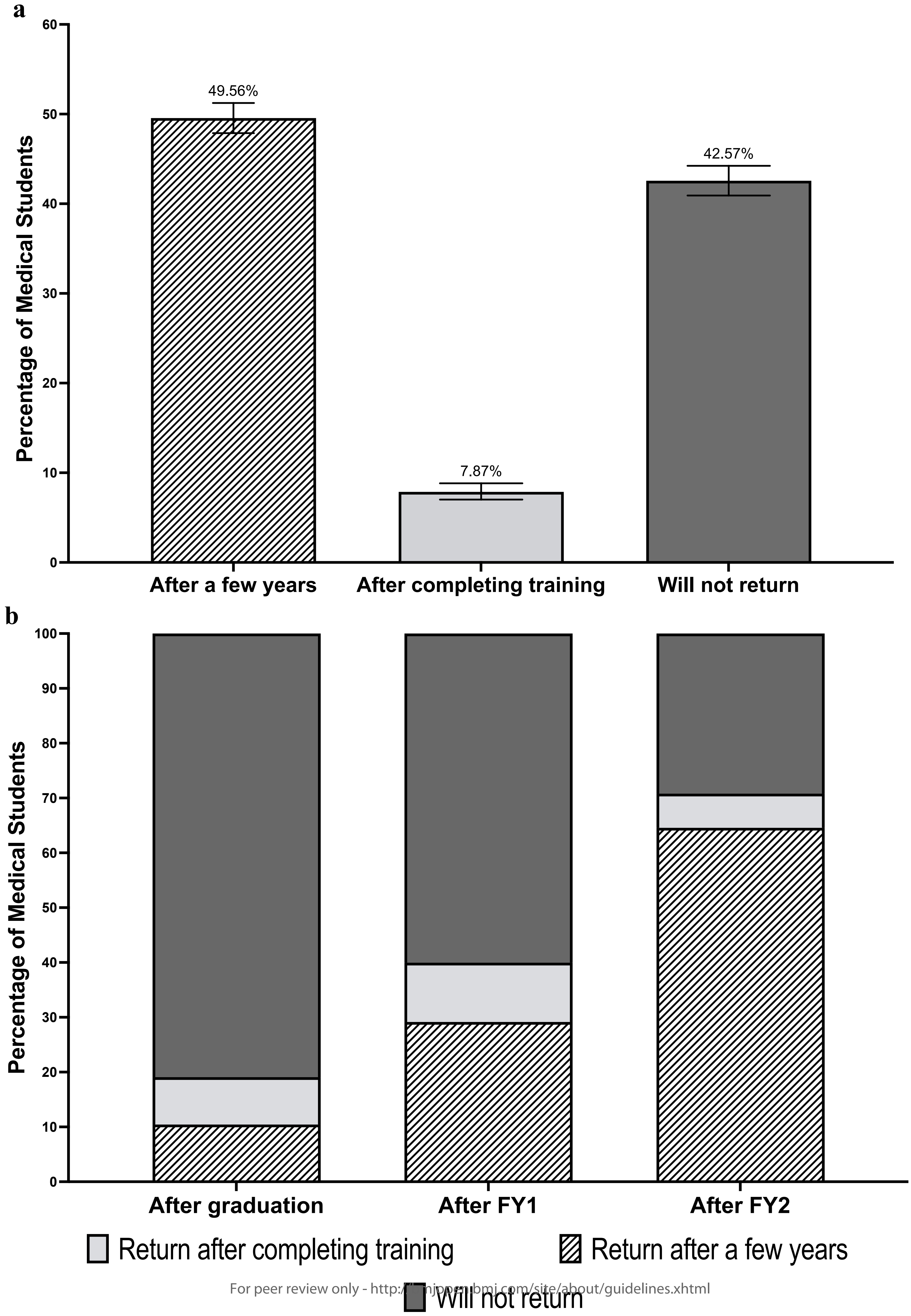
a
Percentage of Medical Students
60
50
40
30
20
10
0
49.56%
7.87%
42.57%
After a few years
After completing training
Will not return
b
Percentage of Medical Students
100
90
80
70
60
50
40
30
20
10
0
After graduation
After FY1
After FY2
Return after completing training
Return after a few years
Will not return

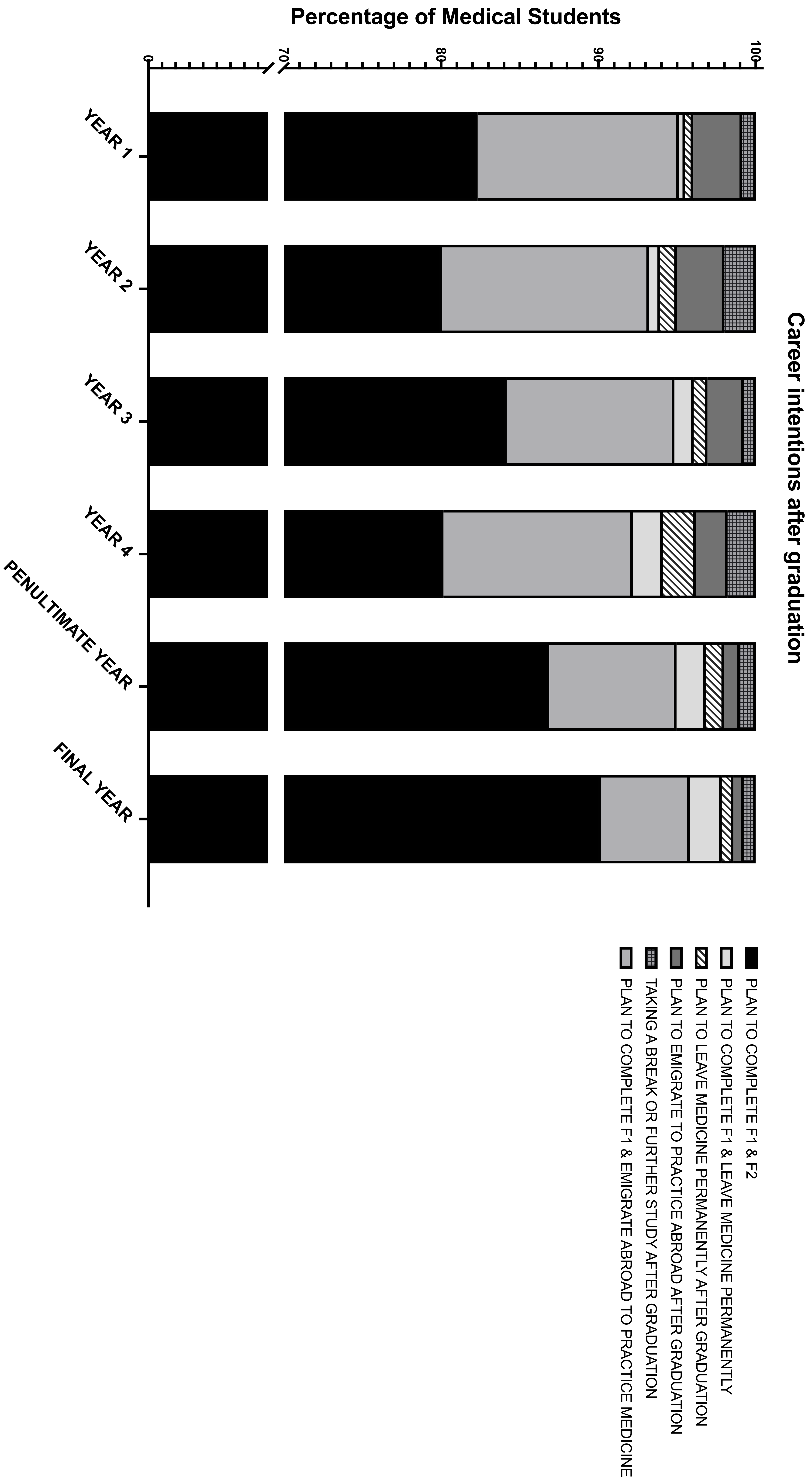
Career intentions after graduation
Percentage of Medical Students
0
70
80
90
100
YEAR 1
YEAR 2
YEAR 3
YEAR 4
PENULTIMATE YEAR
FINAL YEAR
PLAN TO COMPLETE F1 & F2
PLAN TO COMPLETE F1 & LEAVE MEDICINE PERMANENTLY
PLAN TO LEAVE MEDICINE PERMANENTLY AFTER GRADUATION
PLAN TO EMIGRATE TO PRACTICE ABROAD AFTER GRADUATION
TAKING A BREAK OR FURTHER STUDY AFTER GRADUATION
PLAN TO COMPLETE F1 & EMIGRATE ABROAD TO PRACTICE MEDICINE

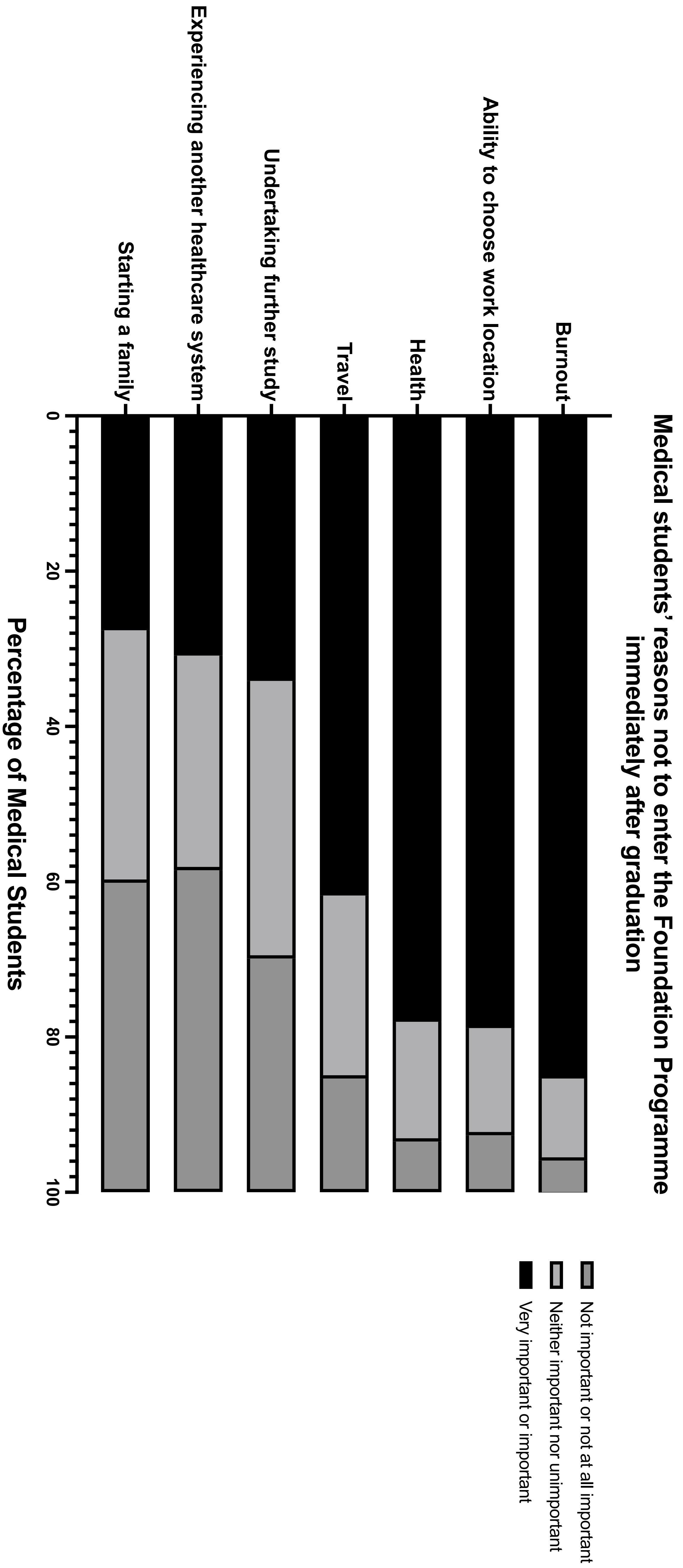
Medical students' reasons not to enter the Foundation Programme immediately after graduation
Burnout
Ability to choose work location
Health
Travel
Undertaking further study
Experiencing another healthcare system
Starting a family
0
20
40
60
80
100
Percentage of Medical Students
Not important or not at all important
Neither important nor unimportant
Very important or important

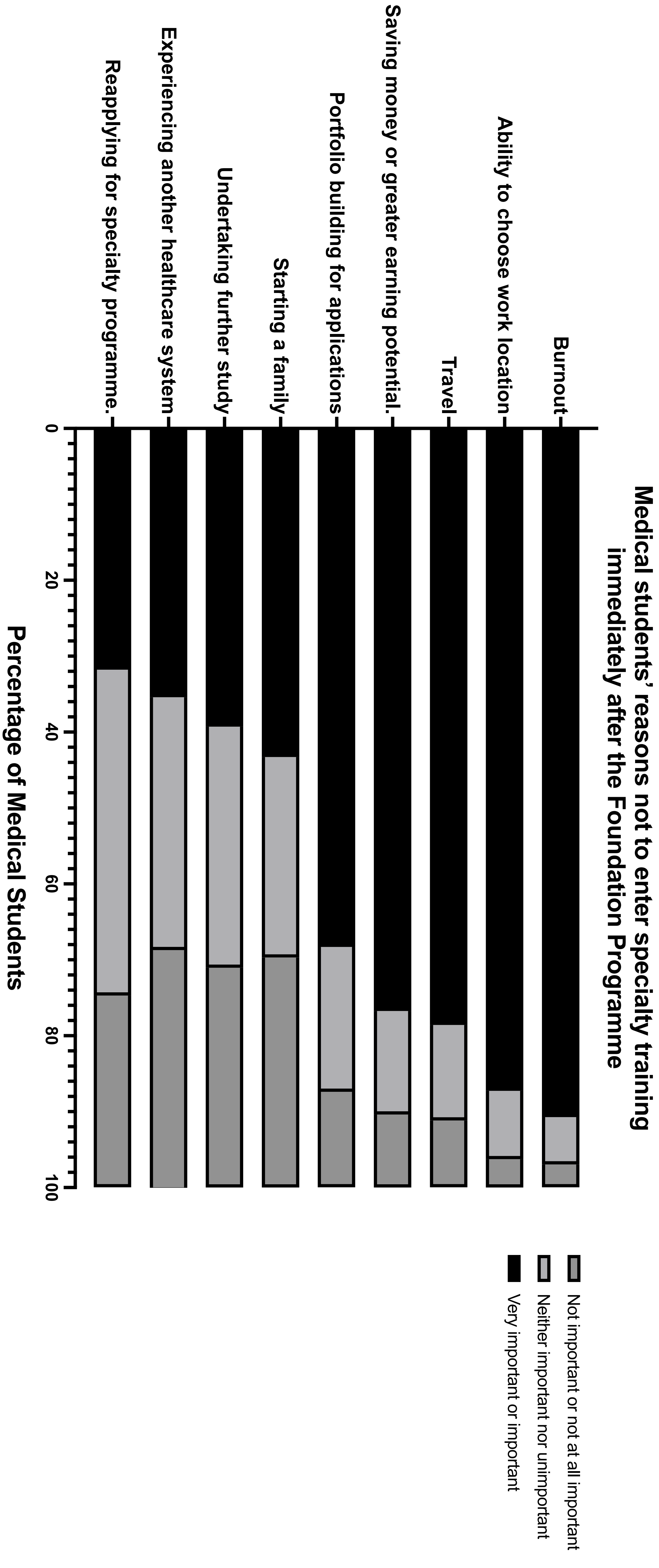
Medical students' reasons not to enter specialty training immediately after the Foundation Programme
Burnout
Ability to choose work location
Travel
Saving money or greater earning potential.
Portfolio building for applications
Starting a family
Undertaking further study
Experiencing another healthcare system
Reapplying for specialty programme.
0
20
40
60
80
100
Percentage of Medical Students
Not important or not at all important
Neither important nor unimportant
Very important or important

Students' intention after graduation	Number (%)	Confidence Interval
Complete both FY1 and FY2	8,806 (83.98)	[83.26, 84.67]
Complete FY1 and emigrate to practice medicine	1,101 (10.50)	[9.93,11.10]
Complete FY1 and leave medicine permanently	132 (1.26)	[1.06, 1.49]
Leave medicine permanently	104 (0.99)	[0.82, 1.20]
Emigrate to practice medicine	220 (2.10)	[1.84, 2.39]
Take a break or undertake further study	123 (1.17)	[0.98, 1.40]

For peer review only - http://bmjopen.bmj.com/site/about/guidelines.xhtml

Students' intention after the Foundation Programme	Number (%)	Confidence Interval
Enter specialty training in the UK	4,294 (48.76)	[47.72, 49.81]
Assume a non-training clinical job in the UK	1,859 (21.11)	[20.27, 21.98]
Emigrate to practice medicine abroad (including temporarily)	2,071 (23.52)	[22.64, 24.42]
Take a break or undertake further study	515 (5.85)	[5.38, 6.36]
Leave medicine permanently	67 (0.76)	[0.60, 0.97]

review only

Students' intention after graduation	Year 1	Year 2	Year 3	Year 4 (not penultimate year)	Penultimate Year	Final Year
Complete both FY1 and FY2	1616 (82.32)	1723 (80.07)	1643 (84.17)	759 (80.15)	1728 (86.88)	1337 (90.16)
Complete FY1 and emigrate to practice medicine	251 (12.79)	283 (13.15)	208 (10.66)	114 (12.04)	161 (8.09)	84 (5.66)
Complete FY1 and leave medicine permanently	8 (0.41)	15 (0.70)	24 (1.23)	18 (1.90)	37 (1.86)	30 (2.02)
Leave medicine permanently	10 (0.51)	23 (1.07)	17 (0.87)	20 (2.11)	23 (1.16)	11 (0.74)
Emigrate to practice medicine	61 (3.11)	65 (3.02)	45 (2.31)	19 (2.01)	20 (1.01)	10 (0.67)
Take a break or undertake further study	17 (0.87)	43 (2.00)	15 (0.77)	17 (1.80)	20 (1.01)	11 (0.74)

For peer review only - http://bmjopen.bmj.com/site/about/guidelines.xhtml

Students' intention after the Foundation Programme	Year 1	Year 2	Year 3	Year 4 (not penultimate year)	Penultimate Year	Final Year
Enter specialty training in the UK	1093 (67.64)	1071 (62.16)	866 (52.71)	298 (39.26)	621 (35.94)	345 (25.80)
Assume a non-training clinical job in the UK	109 (6.75)	197 (11.43)	326 (19.84)	192 (25.30)	554 (32.06)	481 (35.98)
Emigrate to practice medicine abroad (including temporarily)	333 (20.61)	365 (21.18)	343 (20.88)	193 (25.43)	435 (25.17)	402 (30.07)
Take a break or undertake further study	78 (4.83)	86 (4.99)	96 (5.84)	68 (8.96)	103 (5.96)	84 (6.28)
Leave medicine permanently	3 (0.19)	4 (0.23)	12 (0.73)	8 (1.05)	15 (0.87)	25 (1.87)

For peer review only - http://bmjopen.bmj.com/site/about/guidelines.xhtml

Demographic subgroup	Return prospects		
	After a few years	After completing training	Will not return
Ethnicity			
White	1,133 (58.46)	131 (6.76)	674 (34.78)
Asian or Asian British	334 (36.66)	78 (8.56)	499 (54.77)
Black, Black British, Caribbean or African	79 (44.89)	15 (8.52)	82 (46.59)
Mixed or multiple ethnic groups	88 (46.07)	23 (12.04)	80 (41.88)
Other	40 (28.37)	18 (12.77)	83 (58.87)
Prefer not to say	7 (20.00)	2 (5.71)	26 (74.29)
Gender			
Female	1,165 (53.37)	175 (8.02)	843 (38.62)
Male	512 (42.99)	92 (7.72)	587 (49.29)
Non-binary	3 (25.00)	0 (0)	9 (75.00)
Prefer not to say	1 (16.67)	0 (0)	5 (83.33)
Level of education			
Postgraduate	311 (46.49)	51 (7.62)	307 (45.89)

Undergraduate	1,370 (50.31)	216 (7.93)	1137 (41.76)
Previous schooling			
Private education	578 (44.91)	113 (8.78)	596 (46.31)
State education	1,072 (52.96)	143 (7.07)	809 (39.97)
Prefer not to say	31 (38.27)	11 (13.58)	39 (48.15)
Fee status			
Home	1,572 (56.67)	221 (7.97)	981 (35.36)
EU	45 (20.74)	17 (7.83)	155 (71.43)
International (Non-EU)	64 (15.96)	29 (7.23)	308 (76.81)
Current year of study			
Year 1	297 (46.05)	65 (10.08)	283 (43.88)
Year 2	346 (48.53)	55 (7.71)	312 (43.76)
Year 3	281 (47.15)	55 (9.23)	260 (43.62)
Year 4 (not penultimate year)	163 (50.00)	25 (7.67)	138 (42.33)
Penultimate year	313 (50.81)	47 (7.63)	256 (41.56)
Final year	281 (56.65)	20 (4.03)	195 (39.31)

Total	1681 (49.56)	267 (7.87)	1444 (42.57)

For peer review only

AIMS UNIVERSITY OF CAMBRIDGE School of Clinical Medicine

Participant Information Sheet

Ascertaining the career Intentions of Medical Students in the UK post-graduation: a cross-sectional study

Thank you for your interest in participating in this study. Please take a moment to read the following information. If you have any questions or concerns, please contact the principal researcher, Tomas Ferreira at tf385@cam.ac.uk.

What is the aim of this study? This study aims to determine current medical students' career intentions post-graduation and post-foundation training, to identify factors involved in decision making for students' career choices and to analyse medical students' views on how the prospect of working in the NHS could be improved.

Why have I been selected to take part? All medical students currently studying at UK medical schools recognised by the General Medical Council (GMC) are being invited to take part in the questionnaire.

What do I have to do? If you decide to participate in this study, you will be asked to complete a questionnaire about your background, your career intentions after graduation and after foundation training, and your motivations for these answers. This study is voluntary. If you choose to participate, you will be asked to complete the survey by clicking on the link found at the end of this document. This survey is expected to take about 4-7 minutes to complete, but there is no time limit. No background knowledge is required. By submitting the survey, you consent to the collection and storage of data in accordance with the UK General Data Protection Regulation (GDPR) within the survey. For more information on GDPR please click on the following link: https://gdpr-info.eu.

Do I have to participate? Participation is entirely voluntary. You may withdraw at any point during the questionnaire for any reason, before submitting your answers, by closing the browser. In cases of withdrawal from the study prior to submission of the survey, no data is recorded. If you have already submitted data and wish to withdraw from the study, please contact tf385@cam.ac.uk by 31st March 2023.

Who has approved this study? This study has been reviewed and approved by the University of Cambridge's Research Ethics Committee on the 5th of January 2023, reference PRE.2022.124.

How will my data be used? All answers will be anonymous, and we will take all reasonable precautions to ensure that they remain confidential. Data will be stored in a password-protected file and may be used in academic publications. Your IP address will not be stored. After completion of data collection, no email addresses will be stored unless you consent to being followed up via the survey's final question. Prior to completion of data collection, we will store your institutional email address to confirm your student status. Research data will be stored for a minimum of ten years after publication or public release.

Who will have access to my data? Qualtrics is the data controller of the personal data held about you and, as such, will determine how your personal data are used. Their privacy notice can be found here: https://www.qualtrics.com/privacy-statement. Qualtrics will share any email address you provide and your anonymised responses with the University of Cambridge, for the purposes of research as highlighted above. Researchers involved in the project will have access to this anonymised data.

Are there any benefits to taking part? Although there are no immediate individual benefits to participating in this survey, you are given the opportunity to contribute to research which may impact you. You may find this survey an opportunity to self-reflect on your career plans after you graduate. Additionally, all participants will be entered into a prize draw for a chance to win £300!

Will the research be published? The findings of this study may be published in peer-reviewed journals, presented at conferences and a summary of the findings will be made available on social media.

For peer review only

UNIVERSITY OF CAMBRIDGE
School of Clinical Medicine
AIMS

Are there any possible risks involved with my participation? There are no anticipated disadvantages, side effects, risks, and/or discomforts of taking part in this study. If participating in the study leads to distress, you may stop the survey at any time. If your distress continues after leaving the survey, we have provided a list of supportive services nationwide that can be helpful and that you might consider contacting (appears at the close of survey).

Who do I contact if I have a concern about the study or I wish to complain? If you have a concern about any aspect of this project, please speak to the principal researcher tf385@cam.ac.uk. If you remain unhappy or wish to make a formal complaint, please contact the Research and Information Governance, School of Clinical Medicine, University of Cambridge: Research.Governance@medschl.cam.ac.uk.

How do I find out what was learned in this study? This study is expected to be completed by April 2023. If you would like a brief summary of the results, please write to us by email to request information

Who to contact for further details? For any further questions or more information on the study, please contact us on the following email address: tf385@cam.ac.uk.

Link to the survey: https://cambridge.eu.qualtrics.com/jfe/form/SV_cx55RTspDLTIzWK

Kind Regards,

Tomas Ferreira
AIMS Study Lead

Dr. Rita Horvath
Supervisor, Director of Research, Horvath Laboratory, Department of Clinical Neurosciences, University of Cambridge

For peer review only

Demographics

AIMS - Ascertaining the career Intentions of UK Medical Students' post-graduation: a cross-sectional survey

Thank you for taking part in the study. Please note that participating in this survey is entirely optional.

In 2010, 83.1% of Foundation Year 2 (F2) doctors went into further training. In 2019, this number was only 34.9%. This represents a significant change in the makeup of doctors in the UK on a backdrop of a wider NHS staffing challenge. AIMS endeavours to understand the factors involved in medical students' decision-making around their future career. Specifically, we are interested in what students' current career plans are, and why they may, or may not choose to pursue specialty training, or a medical career more broadly, in the UK. We are also hoping to understand current views on the prospect of working in the NHS.

All responses will remain confidential. Your email address will only be visible to the study leads and will be deleted from our records once all data has been collected (unless you consent to being followed up at the end of the survey) and there is no need for further communication. You may withdraw from the study at any point by contacting Tomas Ferreira, tf385@cam.ac.uk.

By submitting your answers to the survey, you consent to us collecting this data and acknowledging that anonymised data may be published and used for purposes beyond this study. Ethical approval was granted by the University of Cambridge Research Ethics Committee (PRE.2022.124) on 5 January 2023.

All participants will be entered into a prize draw for the chance to win £300!

I understand that my participation is voluntary and that I am free to withdraw at any time without giving a reason and I consent to participate in this study.

○ Yes

Email Address

Please enter your institutional email address (ending in 'ac.uk'. We will use this to verify your student status and we may contact you to notify you of a prize win or for clarification of responses). Please ensure there are no spaces at the end of your email.

Age

Gender

Which of the below options best describes your ethnicity?

University

Year of study - Please read description.

(as of September 2022)

- If you are in your fourth year of study and it is your final year, please select final year (i.e., GEM)

- If you are in your fourth year of study but it is your penultimate year, please select penultimate year.

- If you are currently intercalating, please select your current year of study (e.g., intercalating between 3rd and 4th year on a 5 year course please select Year 4).

- Treat the first year of a GEM course as still equivalent to first year.

- If you are in a "Foundation" or "Gateway" year (also known as Y0), please select Year 1.

- ◯ Year 1
- ◯ Year 2
- ◯ Year 3
- ◯ Year 4 (not penultimate year)
- ◯ Penultimate year
- ◯ Final year

What is your expected graduation year?

Do you have a previous or intercalated degree?

- ◯ Yes, prior to studying Medicine.
- ◯ Yes, an intercalated degree.
- ◯ Yes, both.
- ◯ Not yet, but intend on intercalating.

- ◯ Not yet, but currently intercalating.
- ◯ No.

What is your student fee status?

- ◯ Home
- ◯ EU
- ◯ International (Non-EU)

Did you, at any point in your education, attend a fee-paying independent school?

E.g., private school.

- ◯ Yes
- ◯ No
- ◯ Prefer not to say

Intentions

Do you intend to join the NHS Foundation Programme after graduation?

- ◯ Yes - plan to complete F1 & F2
- ◯ Yes - plan to complete F1 & emigrate to practice abroad
- ◯ Yes - plan to complete F1 & leave medicine permanently.
- ◯ No - plan on leaving medicine permanently.
- ◯ No - plan on emigrating
- ◯ No - plan on taking a break or undertaking further study.

What do you intend to do after completing the NHS Foundation Programme?

- ◯ Enter specialty training in the UK
- ◯ Non-training clinical job in the UK, e.g. 'F3 year', JCF or CTF
- ◯ Emigrating to practice medicine abroad (including temporarily)
- ◯ Taking a break or undertaking further study

○ Leaving medicine permanently

You have indicated your intention to leave medicine permanently. In which industry do you plan to work after leaving medicine? If unsure, please enter N/A"

In which country do you intend to practice?

If you are unsure, please enter N/A.

Reasons for emigrating to practice abroad

In your previous answers, you have indicated your intentions to practice medicine abroad.

Please indicate the level of importance of the below factors in your decision making

	Very important	Important	Neither important nor unimportant	Not important	Not at all important
Remuneration or pay at junior level	○	○	○	○	○
Remuneration or pay at consultant level	○	○	○	○	○
Work-life balance	○	○	○	○	○
Family	○	○	○	○	○
Desire for a life change	○	○	○	○	○
Ease of entry into training (competition)	○	○	○	○	○
Length of training	○	○	○	○	○
Standard of training	○	○	○	○	○
Ability to choose work location	○	○	○	○	○

Working conditions of a doctor in the NHS	○	○	○	○	○
Uncertainty about which specialty to pursue	○	○	○	○	○

You have indicated that you intend to emigrate to practice medicine, do you intend on returning to the UK?

- ○ Yes - after a few years
- ○ Yes - after I complete my training
- ○ No

Reasons for leaving medicine permanently

In your previous answers, you have indicated your intentions to leave medicine permanently.

Please indicate the level of importance of the below factors in your decision making.

	Very important	Important	Neither important nor unimportant	Not important	Not at all important
Remuneration or pay at junior level	○	○	○	○	○
Remuneration or pay at consultant level	○	○	○	○	○
Work-life balance	○	○	○	○	○
Family	○	○	○	○	○
Desire for a life change	○	○	○	○	○
Ability to choose work location	○	○	○	○	○
Working conditions of a doctor in the NHS	○	○	○	○	○
Stress levels associated with profession	○	○	○	○	○
Burnout	○	○	○	○	○

Reasons for not entering specialty training immediately after F2

In your previous answers, you have indicated your intentions to not enter specialty training immediately after completing your F2 year. Please indicate the level of importance of the below factors in your decision making.

	Very important	Important	Neither important nor unimportant	Not important	Not at all important
Saving money or greater earning potential.	○	○	○	○	○
Undertaking further study	○	○	○	○	○
Portfolio building for applications	○	○	○	○	○
Travel	○	○	○	○	○
Experiencing another healthcare system	○	○	○	○	○
Starting a family	○	○	○	○	○
Reapplying for specialty programme.	○	○	○	○	○
Ability to choose work location	○	○	○	○	○
Burnout	○	○	○	○	○
Uncertainty about which specialty to pursue	○	○	○	○	○

Reasons for not entering foundation training immediately after graduation

In your previous answers, you have indicated your intentions to not enter foundation training immediately after graduation. Please indicate the level of importance of the below factors in your decision making.

	Very important	Important	Neither important nor unimportant	Not important	Not at all important

Undertaking further study	○	○	○	○	○
Travel	○	○	○	○	○
Experiencing another healthcare system	○	○	○	○	○
Starting a family	○	○	○	○	○
Ability to choose work location	○	○	○	○	○
Health	○	○	○	○	○
Burnout	○	○	○	○	○

Views on a career in the NHS

For each of the points below, how would you describe your level of satisfaction regarding their current status in the NHS?

	Very satisfied	Satisfied	Neither satisfied nor unsatisfied	Not satisfied	Not at all satisfied
Remuneration or pay at junior level	○	○	○	○	○
Remuneration or pay at consultant level	○	○	○	○	○
Work-life balance	○	○	○	○	○
Ability to choose work location	○	○	○	○	○
Ease of entry into training (competition)	○	○	○	○	○
Length of training	○	○	○	○	○
Standard of training	○	○	○	○	○
Working conditions of a doctor in the NHS	○	○	○	○	○
Exposure to desired specialty during foundation programme	○	○	○	○	○
Theatre time during foundation programme	○	○	○	○	○

Cost of training (i.e., mandatory exams, courses, memberships)	○	○	○	○	○
Length of time to decide on a specialty	○	○	○	○	○
Pension Tax rules as a consultant	○	○	○	○	○
Overall satisfaction with the prospect of working in the NHS	○	○	○	○	○

Are you certain about which specialty you wish to pursue?

- ○ Very certain
- ○ Somewhat certain
- ○ Neither certain nor uncertain
- ○ Somewhat uncertain
- ○ Very uncertain

Which specialty (or specialties) most interest you?

Select **up to** a maximum of 3 options (if you are certain, please select only one)

- ☐ Acute internal medicine
- ☐ Allergy
- ☐ Anaesthetics
- ☐ Audio vestibular medicine
- ☐ Cardio-thoracic surgery
- ☐ Cardiology
- ☐ Clinical genetics
- ☐ Clinical neurophysiology
- ☐ Clinical oncology
- ☐ Community sexual and reproductive health
- ☐ Dermatology
- ☐ Emergency medicine
- ☐ Endocrinology and diabetes mellitus
- ☐ Gastro-enterology
- ☐ General practice
- ☐ General surgery

- ☐ Genito-urinary medicine
- ☐ Geriatric medicine
- ☐ Haematology
- ☐ Histopathology
- ☐ Immunology
- ☐ Infectious diseases
- ☐ Intensive care medicine
- ☐ Medical microbiology
- ☐ Medical oncology
- ☐ Neurology
- ☐ Neurosurgery
- ☐ Nuclear medicine
- ☐ Obstetrics and gynaecology
- ☐ Occupational medicine
- ☐ Ophthalmology
- ☐ Oral and maxillo-facial surgery
- ☐ Otolaryngology (ENT)
- ☐ Paediatric surgery
- ☐ Paediatrics
- ☐ Palliative medicine
- ☐ Pathology
- ☐ Plastic surgery
- ☐ Psychiatry
- ☐ Public health medicine
- ☐ Radiology
- ☐ Rehabilitation medicine
- ☐ Renal medicine
- ☐ Respiratory medicine
- ☐ Rheumatology
- ☐ Sport and exercise medicine
- ☐ Trauma and orthopaedic surgery
- ☐ Tropical medicine
- ☐ Urology
- ☐ Vascular surgery

What steps could be taken to improve the prospect of working in the NHS?

(Optional)

Do you consent to being contacted by us for potential follow-up studies regarding your career intentions?

We will store your email address to contact you in the future.

- Yes
- No

Powered by Qualtrics

For peer review only

Eligible Medical Schools and Approved Programmes

A combination of the universities of Dundee and St. Andrews (ScotGEM)
A combination of the University of Brighton and the University of Sussex
A combination of the University of Hull and the University of York
Anglia Ruskin School of Medicine
Aston Medical School
Brunel University London Medical School
Cardiff University
Edge Hill University Medical School
Imperial College London
Keele University
Kent and Medway Medical School
King's College London
Lancaster University
Queen Mary University of London
St George's University of London
Swansea University
The Queen's University of Belfast
The University of Aberdeen
The University of Birmingham
The University of Bristol
The University of Buckingham
The University of Cambridge
The University of Central Lancashire
The University of Dundee
The University of Dundee
The University of East Anglia
The University of Edinburgh
The University of Exeter
The University of Glasgow
The University of Leeds
The University of Leicester
The University of Liverpool
The University of Manchester
The University of Newcastle
The University of Nottingham
The University of Oxford
The University of Plymouth
The University of Sheffield
The University of Southampton
The University of St Andrew's
The University of Warwick
Ulster University School of Medicine
University College London
University of Sunderland School of Medicine

Excluded for lack of cohort at time of recruitment:

- University of Chester Medical School
- Three Counties Medical School

For peer review only

STROBE Statement—Checklist of items that should be included in reports of ***cross-sectional studies***

	Item No	Recommendation
**Title and abstract**	1	(*a*) Indicate the study's design with a commonly used term in the title or the abstract
		(*b*) Provide in the abstract an informative and balanced summary of what was done and what was found
**Introduction**		
Background/rationale	2	Explain the scientific background and rationale for the investigation being reported
Objectives	3	State specific objectives, including any prespecified hypotheses
**Methods**		
Study design	4	Present key elements of study design early in the paper
Setting	5	Describe the setting, locations, and relevant dates, including periods of recruitment, exposure, follow-up, and data collection
Participants	6	(*a*) Give the eligibility criteria, and the sources and methods of selection of participants
Variables	7	Clearly define all outcomes, exposures, predictors, potential confounders, and effect modifiers. Give diagnostic criteria, if applicable
Data sources/ measurement	8*	For each variable of interest, give sources of data and details of methods of assessment (measurement). Describe comparability of assessment methods if there is more than one group
Bias	9	Describe any efforts to address potential sources of bias
Study size	10	Explain how the study size was arrived at
Quantitative variables	11	Explain how quantitative variables were handled in the analyses. If applicable, describe which groupings were chosen and why
Statistical methods	12	(*a*) Describe all statistical methods, including those used to control for confounding
		(*b*) Describe any methods used to examine subgroups and interactions
		(*c*) Explain how missing data were addressed
		(*d*) If applicable, describe analytical methods taking account of sampling strategy
		(*e*) Describe any sensitivity analyses
**Results**		
Participants	13*	(a) Report numbers of individuals at each stage of study—eg numbers potentially eligible, examined for eligibility, confirmed eligible, included in the study, completing follow-up, and analysed
		(b) Give reasons for non-participation at each stage
		(c) Consider use of a flow diagram
Descriptive data	14*	(a) Give characteristics of study participants (eg demographic, clinical, social) and information on exposures and potential confounders
		(b) Indicate number of participants with missing data for each variable of interest
Outcome data	15*	Report numbers of outcome events or summary measures
Main results	16	(*a*) Give unadjusted estimates and, if applicable, confounder-adjusted estimates and their precision (eg, 95% confidence interval). Make clear which confounders were adjusted for and why they were included
		(*b*) Report category boundaries when continuous variables were categorized
		(*c*) If relevant, consider translating estimates of relative risk into absolute risk for a meaningful time period
Other analyses	17	Report other analyses done—eg analyses of subgroups and interactions, and sensitivity analyses

For peer review only

For peer review only - http://bmjopen.bmj.com/site/about/guidelines.xhtml

**Discussion**		
Key results	18	Summarise key results with reference to study objectives
Limitations	19	Discuss limitations of the study, taking into account sources of potential bias or imprecision. Discuss both direction and magnitude of any potential bias
Interpretation	20	Give a cautious overall interpretation of results considering objectives, limitations, multiplicity of analyses, results from similar studies, and other relevant evidence
Generalisability	21	Discuss the generalisability (external validity) of the study results
**Other information**		
Funding	22	Give the source of funding and the role of the funders for the present study and, if applicable, for the original study on which the present article is based

*Give information separately for exposed and unexposed groups.

Note: An Explanation and Elaboration article discusses each checklist item and gives methodological background and published examples of transparent reporting. The STROBE checklist is best used in conjunction with this article (freely available on the Web sites of PLoS Medicine at http://www.plosmedicine.org/, Annals of Internal Medicine at http://www.annals.org/, and Epidemiology at http://www.epidem.com/). Information on the STROBE Initiative is available at www.strobe-statement.org.

1. a) "Career intentions of medical students in the United Kingdom: a national, cross-sectional study (AIMS Study)"
1. b) This has been done (Page 2).
2. The scientific background and rationale for the investigation can be found in page 3.
3. This can be found in the last paragraph of the introduction section in page 3.
4. Page 3 and Page 4
5. Page 4
6. Page 4
7. Page 3 and 4
8. Page 4
9. Page 4
10. Protocol "A sample size calculation was performed, and it was determined that a minimum of 8,026 participants are needed to have a confidence level of 95% that the results of the survey are within 1% representation of the total medical student population. This calculation used a population size for UK medical students acquired via a Freedom of Information request to the GMC." (https://www.researchprotocols.org/2023/1/e45992). In reality, 10,486 responses received.
11. Page 4
12. Page 4
13. Pages 5-12. All questions were mandatory other than qualitative thematic analysis for which response proportion was given (page 12)
14. Pages 5 and 6. No missing data.
15. Pages 6-12.
16. Pages 6-12.
17. Pages 9-12.
18. Pages 13-15.
19. Page 2 and Page 15.
20. Pages 13-15.
21. Pages 13-15.
22. Page 17.

For peer review only - http://bmjopen.bmj.com/site/about/guidelines.xhtml
